# Long intergenic non-coding RNA 271 is predictive of a poorer prognosis of papillary thyroid cancer

**DOI:** 10.1038/srep36973

**Published:** 2016-11-11

**Authors:** Ben Ma, Tian Liao, Duo Wen, Chuanpeng Dong, Li Zhou, Shuwen Yang, Yu Wang, Qinghai Ji

**Affiliations:** 1 Department of Head and Neck Surgery, Fudan University Shanghai Cancer Center, Shanghai, People’s Republic of China; 2 Department of Oncology, Shanghai Medical College, Fudan University, Shanghai, People’s Republic of China; 3 Institute of Biomedical Science, Shanghai Medical College, Fudan University, Shanghai, People’s Republic of China

## Abstract

A number of long non-coding RNAs (lncRNAs) have been found to play critical roles in oncogenesis and tumor progression. We aimed to investigate whether lncRNAs could act as prognostic biomarkers for papillary thyroid cancer (PTC) that may assist us in evaluating disease status and prognosis for patients. We found 220 lncRNAs with expression alteration from the annotated 2773 lncRNAs approved by the HUGO gene nomenclature committee in The Cancer Genome Atlas (TCGA) dataset, of which FAM41C, CTBP1-AS2, LINC00271, HAR1A, LINC00310 and HAS2-AS1 were associated with recurrence. After adjusting classical clinicopathogical factors and *BRAF*^*V600E*^ mutation, LINC00271 was found to be an independent risk factor for extrathyroidal extension, lymph node metastasis, advanced tumor stage III/IV and recurrence in multivariate analyses. Additionally, LINC00271 expression was significantly downregulated in PTCs versus adjacent normal tissues (P < 0.001). The Gene Set Enrichment Analysis (GSEA) revealed that genes associated with cell adhesion molecules, cell cycle, P53 signaling pathway and JAK/STAT signaling pathway were remarkably enriched in lower-LINC00271 versus higher-LINC00271 tumors. In conclusion, LINC00271 was identified as a possible suppressor gene in PTC in our study, and it may serve as a potential predictor of poor prognoses in PTC.

Differentiated thyroid cancer (DTC), arising from thyroid follicular epithelial cells, accounts for the vast majority of thyroid cancers. Of the DTCs, papillary thyroid cancer (PTC) is the most common histological type^1^. The yearly incidence of thyroid cancer in the United States has nearly tripled from 4.9 per 100,000 in 1975 to 14.3 per 100,000 in 2009, and almost the entire change has been attributed to an increase in the incidence of PTC^2^,^3^. In general, most PTCs are indolent in biological processes and can be cured with thyroidectomy and radioiodine therapy. However, approximately 5–20% of patients can suffer disease recurrence^4^, and occasionally some progress to aggressive and lethal outcomes. The wide spectrum of PTC behaviors may attribute to variable genetic backgrounds and molecular events.

Recent efforts on DNA and RNA sequencing data have mainly focused on protein coding exons and transcripts to provide deep insights into genomic characteristics of PTC^5^. Interestingly, areas of non-coding transcripts that account for ~50% of all transcribed RNAs^6^,^7^ are poorly explored. Of the non-coding transcripts, long non-coding RNAs (lncRNAs) refer to the ones that vary from 200 bp to tens of kilobases in length. So far, an overwhelming number of lncRNAs have been identified^8^,^9^, and a certain subset of lncRNAs have been found to play critical roles in oncogenesis and tumor progression^10^,^11^,^12^,^13^. Increasing evidences indicate that, in many types of cancers, lncRNAs are aberrantly expressed and are involved in regulation of diverse biological functions^14^,^15^,^16^,^17^,^18^, suggesting their potential as biomarkers for cancer. Moreover, recent reports^18^,^19^,^20^ have uncovered the potential of lncRNAs as biomarkers for clinical outcomes in many cancers. However, the possible roles and prognostic values of lncRNA signature and specific lncRNAs have not been elucidated clearly in PTC. Older age at diagnosis, poor histological subtypes, extrathyroidal extension (ETE), lymph node metastasis (LNM), and advanced tumor stage are conventional clinicopathological parameters considered as prognostic factors for poor clinical outcomes. The present study aimed to identify novel markers from the annotated lncRNAs for prediction of poor prognoses that may assist us in evaluating disease status and prognosis for PTC patients.

## Results

### LncRNAs with expression alteration in PTC

The annotated 2773 lncRNAs were searched for expression data in PTC at The cBioPortal for Cancer Genomics (Supplementary Table S1). Of the 2773 lncRNAs, 220 lncRNAs with over-expression or under-expression alteration in PTC patients were selected based on the Onco Query Language criteria “EXP < = −2, EXP > = 2” from The Cancer Genome Atlas (TCGA) dataset, and the alteration frequencies were listed in Supplementary Figure S1. According to the cBioPortal for Cancer Genomics, the expression alteration frequency of the 220 lncRNAs ranged from 1% to 7% in 486 PTC patients with RNA sequencing data.

### Clinicopathological data of patients in the TCGA and FUSCC cohorts

A total of 471 PTC patients (126 males and 345 females; mean age: 47.02 ± 15.89 years, range: 15–89 years) with detailed clinicopathological data and expression data of lncRNAs from the TCGA cohort were enrolled in this study. We also included the Fudan University Shanghai Cancer Center (FUSCC) cohort comprising 185 PTC patients (142 females and 43 males) with mean age of 43.30 ± 11.09 years (range: 16–75 years). Tumor characteristics including multifocality, histological subtypes, coexistence of Hashimoto’s thyroiditis (HT), ETE, tumor-node-metastasis (TNM) stage and *BRAF*^*V600E*^ mutation were summarized in Table 1.

### Association of LINC00271 with aggressive PTC

**Figure 1:**
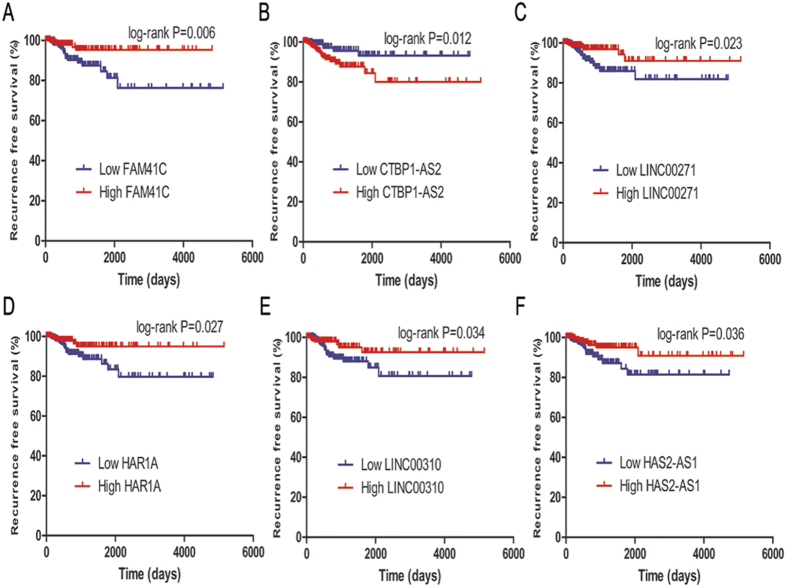
Identification of the annotated lncRNAs associated with recurrence of PTC. (**A–F**) Kaplan–Meier plots of recurrence free survival in the TCGA cohort are shown according to FAM41C, CTBP1-AS2, LINC00271, HAR1A, LINC00310 and HAS2-AS1 expression, respectively.

**Figure 2:**
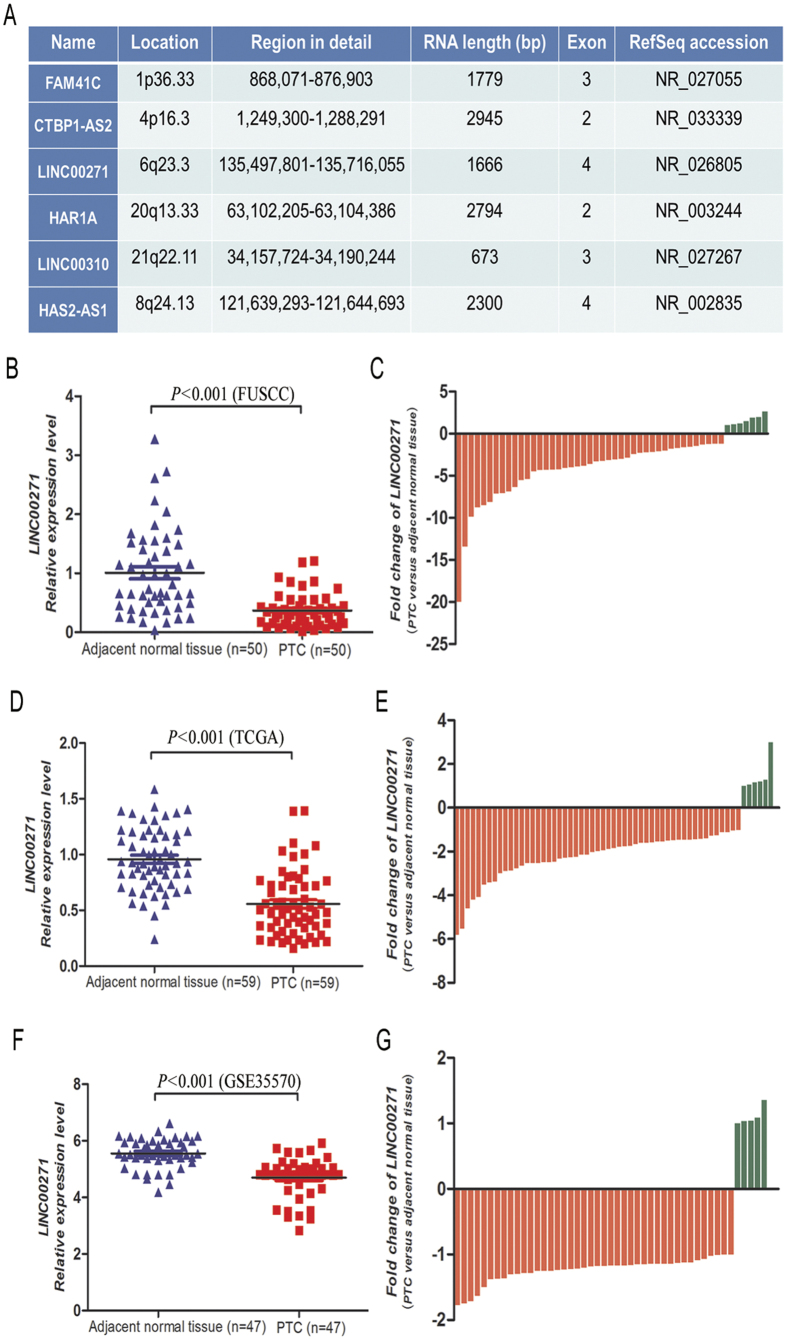
Annotations of the six identified lncRNAs and comparison of LINC00271 expression between PTC and adjacent normal tissues. (**A**) Organizations of the six lncRNAs and chromosome locations. (**B–G**) (**B,D,F**) showed LINC00271 expression was significantly downregulated in PTC compared with the level in the adjacent normal tissues in the FUSCC, TCGA and GSE35570 cohorts. (**C,E,G**) indicated 86.0% (43/50), 89.8% (53/59) and 89.4% (42/47) of the cases with negative fold changes of LINC00271 expression in PTCs compared with adjacent normal tissues in the FUSCC, TCGA and GSE35570 cohorts, respectively.

To investigate whether lncRNAs can act as potential biomarkers for recurrence in PTC, we performed kaplan-Meier analyses on the 220 lncRNAs and recurrence free survival (RFS) in the TCGA cohort. As shown in Fig. 1, six lncRNAs including FAM41C (Fig. 1A, *P* = 0.006), CTBP1-AS2 (Fig. 1B, *P* = 0.012), LINC00271 (Fig. 1C, *P* = 0.023), HAR1A (Fig. 1D, *P* = 0.027), LINC00310 (Fig. 1E, *P* = 0.034) and HAS2-AS1 (Fig. 1F, *P* = 0.036) were screened out to show significant correlations with RFS. The signatures and annotations of the six lncRNAs were summarized in Fig. 2A. Table 2 showed, in univariate hazards proportional analysis calculated by hazard ratio (HR) and 95% confidence interval (CI), FAM41C (HR = 3.394, 95%CI:1.354–8.507, *P* = 0.009), LINC00271 (HR = 2.655, 95%CI:1.109–6.359, *P* = 0.028), HAR1A (HR = 2.699, 95%CI:1.077–6.761, *P* = 0.034), LINC00310 (HR = 2.494, 95%CI: 1.041–5.972, *P* = 0.040) and HAS2-AS1 (HR = 2.389, 95%CI: 1.030–5.541, *P* = 0.043) at low expression levels were risk factors for poor RFS, and overexpression of CTBP1-AS2 (HR = 3.045, 95%CI: 1.215–7.633, *P* = 0.018) increased the risk of recurrence.

In addition, we conducted a further analysis for associations of the six lncRNAs with ETE, LNM, T3-T4 and III-IV stage. As shown in Table 2, among the six lncRNAs, decreased expression of LINC00271 significantly increased risks of ETE (odds ratio (OR) = 2.523, 95%CI: 1.673–3.805, *P* < 0.001), LNM (OR = 1.753, 95%CI: 1.193–2.574, *P* = 0.004), T3/T4 stage (OR = 2.377, 95%CI: 1.628–3.472, *P* < 0.001) and advanced TNM stage III/IV (OR = 2.464, 95%CI: 1.656–3.665, *P* < 0.001) in univariate logistic regression analysis, suggesting it as an optimal biomarker for aggressive behaviors of PTC. To validate the correlation of LINC00271 with the clinicopathological characteristics of PTC patients, LINC00271 expression in the FUSCC cohort was analyzed. Low LINC00271 expression was found to correlate with male gender (*P* = 0.006), larger tumor size (*P* = 0.038) and LNM (*P* = 0.024), showing a lower expression level in PTC with aggressive behaviors (Supplementary Table S2).

### LINC00271 was downregulated in PTC compared with adjacent normal tissues

To determine the role of LINC00271 in PTC, we initially detected LINC00271 expression in the carcinoma specimens and the paired normal tissues in 50 PTC patients, and the results suggested LINC00271 was significantly downregulated in PTC compared with the level in the adjacent normal tissues (*P* < 0.001, Fig. 2B,C). While confirmed in the validation cohorts including the TCGA and GSE35570 cohorts, LINC00271 expression was also found to be suppressed in tumor tissues in comparison with normal tissues (Fig. 2D–G). Additionally, significantly repressed expression of LINC00271 was observed in breast invasive carcinoma (BRCA, Supplementary Figure S2A), lung adenocarcinoma (LUAD, Supplementary Figure S2B), kidney renal papillary cell carcinoma (KIRP, Supplementary Figure S2C) and head and neck squamous cell carcinoma (HNSCC, Supplementary Figure S2>D) when we performed an analysis of LINC00271 expression in BRCA, LUAD, KIRP, HNSCC, liver hepatocellular carcinoma (LIHC) and prostate adenocarcinoma (PRAD) from the TCGA database (Supplementary Figure S2).

### LINC00271 as an independent risk factor for poor clinical outcomes of PTC

Multivariate analyses were performed to confirm whether associations of LINC00271 with high-risk pathological outcomes (ETE, LNM and III/IV stage) and recurrence were independent of classical clinicopathogical factors and *BRAF*^*V600E*^ mutation. Table 3 showed that LINC00271 < cutoff remained significantly correlated with ETE, LNM and III/IV stage in the TCGA cohort in multivariate logistic regression analyses though LINC00271 < cutoff was only found to statistically increase the risk of LNM in the FUSCC cohort. In Table 4, the TCGA cohort indicated advanced T stage (T3-T4, HR = 2.294, 95%CI: 1.010–5.211, *P* = 0.047) and LINC00271 < cutoff (HR = 3.688, 95%CI: 1.384–9.829, *P* = 0.009) were risk factors for poor RFS in univariate cox proportional hazards analysis, and female gender was a protective factor for improved RFS. Multivariate analysis adjusted by age, gender, histological subtypes, T stage, TNM stage, showed female gender (HR = 0.386, 95%CI: 0.172–0.868, *P* = 0.021) and LINC00271 (HR = 3.182, 95%CI: 1.160–8.726, *P* = 0.025) were still statistically significant. Then, we performed a ROC analysis to evaluate the predictive values of LINC00271, gender and TNM stage for PTC recurrence. After combining LINC00271, the AUC values of gender and TNM stage for predicting recurrence were significantly elevated from 0.611 to 0.697 (*P* = 0.020) and 0.597 to 0.677 (*P* = 0.016), respectively (Supplementary Figure S3).

Furthermore, to investigate independent factors that might affect LINC00271 expression in PTC, we conducted a multivariate logistic regression analysis in the TCGA and FUSCC cohorts (Supplementary Table S3). LNM was shown to be significantly correlated with low expression of LINC00271 in both TCGA and FUSCC cohorts.

### Identification of LINC00271-associated biological pathways by GSEA

**Figure 3:**
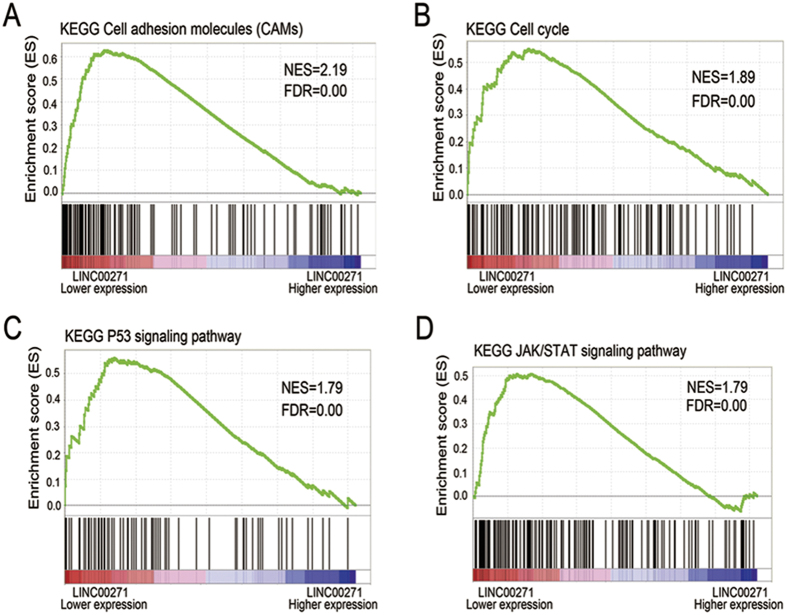
LINC00271-associated biological signaling pathways. (**A–D**) Based on the TCGA dataset, GSEA showed genes associated with CAMs, cell cycle, P53 signaling pathway and JAK/STAT signaling pathway were significantly enriched in lower-LINC00271 vs higher-LINC00271 tumors.

To identify LINC00271-associated biological signaling pathways on an unbiased basis, we performed Gene Set Enrichment Analysis (GSEA) using high throughput RNA-sequencing data of the TCGA cohort. The expression level of LINC00271 was used as the phenotype label. Among all the predefined KEGG pathways gene sets, cell adhesion molecules (CAMs), cell cycle, P53 signaling pathway and JAK/STAT signaling pathway were found to be significantly associated with LINC00271 expression in the TCGA cohort (Fig. 3), suggesting that LINC00271 may be involved in PTC development and progression through the above cancer-associated signaling pathways.

## Discussion

Large numbers of lncRNAs have been identified through genome-wide transcriptome analyses^21^,^22^. A series of studies have revealed that lncRNAs can act as regulators of diverse biological functions including X-chromosome silencing^23^, transcription regulation^24^, P53 function^25^, and cell growth control^26^. Recently, roles and functions of more annotated specific lncRNAs in cancer have been characterized. HOTAIR is shown to promote breast cancer metastasis by reprograming chromatin state, and HOTAIR expression level has prognostic value for metastasis and survival^27^. Leukemia-induced non-coding activator RNA-1 (LUNAR1) is revealed as a novel regulator of IGF1 signaling and T-ALL cell growth and may be a potential biomarker and therapeutic target in acute leukemia^15^. Ewing sarcoma-associated transcript 1 (EWSAT1) is found to facilitate Ewing sarcoma oncogenesis by mediating EWS-FLI1 suppression pathways^16^. *BRAF*-regulated lncRNA (BANCR) induced by *BRAF*^*V600E*^ gene can regulate carcinoma cell migration in melanoma^14^. Furthermore, the potential of lncRNAs as biomarkers for cancer have been confirmed in many cancers. Wang GH *et al*.^28^ revealed that LINC01207 overexpression was associated with advanced TNM stage and shorter survival in lung adenocarcinoma patients. Zhang EB *et al*.^19^ found that ANRIL, as a growth regulator, may serve as a candidate prognostic biomarker and target for new therapies in human gastric cancer. Jin Meng *et al*.^29^ reported that a four-long non-coding RNA signature can be predictive of breast cancer survival, and Liu HR^18^ also identified several lncRNAs with prognostic values for breast cancer. Though recent efforts on protein coding exons and transcripts provided deep insights into genomic characteristics of PTC^5^, the possible roles and prognostic values of lncRNA signature and specific lncRNAs have been poorly characterized in PTC.

As reported in the previous study^18^, the cancer dataset of TCGA at cBioPortal were available for investigation of expression and clinical significance of lncRNAs. In the present study, the TCGA dataset was used to screen out the potential annotated lncRNAs with prognostic values for PTC. Initially, we performed an associative analysis of the annotated lncRNAs with patients’ clinicopathological parameters by using the PTC data of TCGA, and then validated the preliminary findings through our own data analysis at FUSCC. As shown in the study, the 220 lncRNAs were found to be altered at expression levels in PTC patients at cBioportal. Of the 220 lncRNAs, FAM41 C, CTBP1-AS2, LINC00271, HAR1A, LINC00310 and HAS2-AS1 were associated with RFS for PTC patients, and decreased LINC00271 expression also significantly increased risks of ETE, LNM, advanced T stage and TNM stage while the other five lncRNAs failed to correlate with these poor outcomes of PTC accordantly, which indicated LINC00271 as the optimal biomarker for aggressive behaviors of PTC. The associative analysis of LINC00271 expression with clinicopathological factors in the FUSCC cohort confirmed the role of enhancing aggressiveness of LINC00271 in PTC though there was no recurrent case in our cohort due to the limited follow-up time.

PTC is usually curable with thyroidectomy followed by radioiodine therapy, but many patients suffer disease recurrence, and some cases even advance to be incurable and fatal^4^,^30^. Therefore, specific biomarkers for risk stratification are helpful to identify patients at high recurrence so active treatment and careful monitoring can be provided. As we know, the conventional risk stratification is based on patient age, gender, tumor size, histological subtypes, the presence or absence of ETE and LNM and tumor stage finally defined by pathology. However, these criteria are always dependent on postoperative pathology and histological outcomes are not defined before surgery, and there exist heterogeneity and uncertainty in the risk evaluation based on these criteria.

As a prognostic marker, *BRAF*^*V600E*^ mutation has received great attention in the past decades for its potential utility in the risk stratification and management of PTC^31^. However, as mentioned in the 2015 American Thyroid Association Management Guidelines for Adult Patients with Thyroid Nodules and Differentiated Thyroid Cancer^2^, *BRAF*^*V600E*^ has limited values in predicting recurrence of PTC and should not impact on the decision for prophylactic central neck dissection in primary tumor^2^,^31^. Likewise, in our study, *BRAF*^*V600E*^ had no predictive values for recurrence and LNM in PTC. Our multivariate analyses showed that associations of LINC00271 with ETE, LNM, advanced tumor stage and recurrence were independent of classical clinicopathogical factors and *BRAF*^*V600E*^ mutation in the TCGA cohort, which suggested that it could act as a potential prognostic predictor for PTC. We had to admit that the limited number of cases and follow-up time and the difference in sample distribution in the FUSCC cohort were responsible for the negative statistical effects of LINC00271 on ETE and advanced tumor stage although the ORs of LINC00271 for the two parameters revealed positive trends.

Furthermore, the comparison of LINC00271 expression between tumor and adjacent normal tissues in the FUSCC, TCGA and GSE35570 cohorts suggested that LINC00271 could act as a suppressor gene in PTC. Likewise, the suppressive role of LINC00271 is observed in many common cancer types including BRCA, LUAD, KIRP and HNSCC. Diverse signaling pathways including cell cycle, P53 signaling and cell adhesion signaling cooperate to initiate and sustain oncogenesis and progression through maintaining proliferative signaling, evading growth suppressors and activating invasion and metastasis^32^. The pathways that LINC00271 may mediate in PTC remain unclear, so we performed GSEA to identify LINC00271-associated biological signaling pathways. GSEA showed low LINC00271 expression was positively associated with CAMs, cell cycle, P53 signaling pathway and JAK/STAT signaling pathway, which may suggest that LINC00271 is involved in PTC development and progression through these cancer-associated pathways.

In spite of observing the role of decreased LINC00271 expression firstly in PTC in our study, the possible mechanism mediated by LINC00271 has not been illuminated. Basic experiments *in vitro* and *in vivo* and large samples of patients with long-term follow-up outcomes are needed to confirm the effects of LINC00271 in PTC in the future.

In conclusion, LINC00271 was identified as a possible suppressor gene in PTC in our study, and it was an independent risk factor for LNM and recurrence of PTC. LINC00271 may serve as a potential predictor for poor clinical outcomes in PTC.

## Materials and Methods

### Annotated lncRNA database search

We performed a primary search for annotated lncRNAs at the website of HUGO gene nomenclature committee (HGNC) (http://www.Genenames.org/) according to a previous study^18^, and a total of 2773 lncRNAs were obtained from the HGNC database (http://www.genenames.org/cgi-bin/statistics). The 2773 lncRNAs were searched for expression data in PTC at The cBioPortal for Cancer Genomics^34^,^35^ (http://www.cbioportal.org/), of which 220 known lncRNAs with upregulation or downregulation were found finally by using Onco Query Language “EXP < = −2 EXP > = 2” from the TCGA data. Expression data of the 220 lncRNAs and the corresponding clinicopathological data from TCGA dataset were downloaded from the website of The cBioPortal for Cancer Genomics (http://www.cbioportal.org/) and Cancer Genomics Browser of University of California Santa Cruz (https://genome-cancer. ucsc. edu/). Paired LINC00271 expression data in tumor and normal tissues were retrieved from TCGA RNA-sequencing data of PTC, BRCA, LUAD, KIRP, HNSCC, LIHC and PRAD by employing The Atlas of Noncoding RNAs in Cancer (TANRIC)^33^ (http://ibl.mdanderson.org/tanric/design/basic/index.html) and Gene Expression Omnibus data with accession number GSE35570^36^ (https://www.ncbi.nlm.nih.gov/geo/).

### Patients and clinicopathological data

A total of 471 primary PTC patients with clinicopthogical data, detailed expression of the 220 lncRNAs and *BRAF*^*V600E*^ mutation were collected from the updated TCGA database according to the methods in the previous studies^37^,^38^. In addition, consecutive samples were selected from 185 patients diagnosed as PTC by pathology at the FUSCC from Mar 2012 to Jan 2015. The data on patients’ clinicopathological features including gender, age at diagnosis, maximum size of tumor, multifocality, HT, histological subtypes, ETE and cervical LNM were retrospectively abstracted from patient records. All the patients were staged using the 2009 TNM classification of American Joint Committee on Cancer/International Union Against Cancer. The selected samples were subjected to repeated evaluation to confirm diagnosis of the above histological characteristics.

### Ethics statement

Each patient provided a written informed consent for his/her specimens and information to be used for research and stored in the hospital database, and this study was approved by the Ethical Committee of the FUSCC (Reference number: 050432-4-1212B). All procedures performed in our study were in accordance with the ethical standards of our institutional research committee and with the 1964 Helsinki declaration and its later amendments or comparable ethical standards.

### *BRAF* ^*V600E*^ mutation detection

According to the manufacturer’s instructions, genomic DNA was extracted from the resected specimens using the QIAamp DNA Mini Kit (QIAGEN, Chatsworth, California). DNA templates were amplified for analysis of mutations in exon 15 of *BRAF* gene using the polymerase chain reaction (PCR) protocol as previously mentioned^39^, followed by a Big Dye (Applied Biosystems, Foster City, CA) reaction for Sanger sequencing. We recognized *BRAF*^*V600E*^ mutation on sequencing electropherograms.

### RNA extraction and real-time qRT-PCR analysis

We extracted total RNA from the specimens using the Trizol reagent (Invitrogen, Carlsbad, CA, USA) according to the manufacturer’s protocol. The extracted RNA was reversely transcribed for cDNA, followed by real-time quantitative reverse transcription-polymerase chain reaction (qRT-PCR) as previously described^20^. The primers for LINC00271 were as follows: GCTATTGGTGGGAGGCTTCAG (Sense), TGGGCTGGACTTAATGACTTGC (Antisense). GAPDH was used as a housekeeping gene. qRT-PCR assays were performed in triplicate for each sample, and the mean value was used for calculation of mRNA expression levels. The relative mRNA expression levels of LINC00271 were determined by the comparative Ct (2^−∆Ct^) method. The amount of target gene expression levels was given as ratios to GAPDH mRNA level.

### Gene Set Enrichment Analysis

GSEA was performed using GSEA software, Version 2.0.1, which was obtained from the Broad Institute (http://www.broad.mit.edu/gsea), as previously described^40^,^41^. Enrichment map was used for visualization of the GSEA results. False discovery rate (FDR) value and normalized enrichment score (NES) were used to sort the pathways enriched in each phenotype after gene set permutations were performed 1000 times for each analysis.

### Statistical Analysis

Categorical data were summarized with frequencies and percentages. The continuous results were expressed as the mean ± standard deviation. Paired-t and independent-t test was used to compare continuous variables in two groups. Associations between continuous variables and categorical variables were evaluated using Mann-Whitney U tests for two groups and Kruskal-Wallis tests for more than two groups. χ2 and Fisher’s exact test were used for categorical variables. The Kaplan-Meier method was used to construct RFS curves, and the univariate survival difference was determined by the log-rank test. To analyze the association between lncRNAs and clinicopathological parameters, patients were divided into two subgroups (Low expression and High expression) according to the median value of lncRNA expression levels. Nonparametric receiver operating characteristic (ROC) analyses were performed to calculate the best cutoff value for LINC00271 expression level that would be predictive of LNM and recurrence. Moreover, univariate and multivariate analysis were performed to determine risk factors for recurrence in PTC using cox proportional hazards models calculated by HR and 95% CI. OR with 95% CI was calculated by logistic regression analysis. A p-value < 0.05 was considered significant. Statistical analyses were performed using the GraphPad Prism 6.0 and SPSS for Windows (SPSS Inc., Chicago, IL).

## Additional Information

**How to cite this article**: Ma, B. *et al*. Long intergenic non-coding RNA 271 is predictive of a poorer prognosis of papillary thyroid cancer. *Sci. Rep*. **6**, 36973; doi: 10.1038/srep36973 (2016).

**Publisher’s note:** Springer Nature remains neutral with regard to jurisdictional claims in published maps and institutional affiliations.

## Supplementary Material

Supplementary Information

## Figures and Tables

**Table 1:** Clinicopathological characteristics of PTC patients in the FUSCC and TCGA cohorts.

Variables	TCGA (*N* = 471)		FUSCC (*N* = 185)		*P* value
	*N*	%	*N*	%	
Age (years)					0.099
<45	217	46.1	99	53.5	
≥45	254	53.9	86	46.5	
Gender					0.374
Male	126	26.8	43	23.2	
Female	345	73.2	142	76.8	
Multifocality					<*0.001*
Unifocal	250	54.2	135	73.0	
Multifocal	211	45.8	50	27.0	
Histological type					<*0.001*
Classical PTC	329	70.9	183	98.9	
Follicular PTC	99	21.3	2	1.1	
Tall-cell PTC	36	7.8	0	0	
Coexistent HT					*0.004*
Yes	66	14.0	44	23.8	
No	405	86.0	141	76.2	
ETE					<*0.001*
Yes	313	66.5	168	90.8	
No	144	30.6	17	9.2	
NA	14	3.0	0	0	
T Stage					<*0.001*
T1-T2	282	59.9	166	89.7	
T3-T4	188	39.9	19	10.3	
NA	1	0.2	0	0	
LNM					<*0.001*
N0	215	45.6	85	45.9	
N1	210	44.6	100	54.1	
NA	46	9.8	0	0	
M					<*0.001*
M0	263	55.8	185	100	
M1	8	1.7	0	0	
NA	200	42.5	0	0	
TNM Stage					<*0.001*
I-II	312	66.2	152	82.1	
III-IV	157	33.3	33	17.9	
NA	2	0.4	0	0	
*BRAF*^*V600E*^					<*0.001*
Mutation	222	37.6	103	55.7	
Wild-type	177	47.1	79	42.7	
NA	72	15.3	3	1.6	

Notes: Italic and bold type indicates statistical significance.

Abbreviations: PTC, papillary thyroid cancer; FUSCC, Fudan University Shanghai Cancer Center; TCGA, The Cancer Genomics Atlas; HT, Hashimoto’s thyroiditis; ETE, extrathyroidal extension; LNM, lymph node metastasis; M, metastasis; TNM, tumor-node-metastas.

**Table 2:** Association of the six lncRNAs with recurrence and high-risk clinicopathological factors of PTC in the TCGA cohort.

lncRNA	RFS		ETE		LNM		T3-T4 stage		TNM stage: III–IV	
	HR (95%CI)	*P* value	OR (95%CI)	*P* value	OR (95%CI)	*P* value	OR (95%CI)	*P* value	OR (95%CI)	*P* value
FAM41C (low *vs* high)	3.394 (1.354–8.507)	***0.009***	1.078 (0.726–1.599)	0.711	0.836 (0.571–1.223)	0.357	0.572 (0.621–1.301)	0.572	0.938 (0.639–1.377)	0.744
CTBP1-AS2 (high *vs* low)	3.045 (1.215–7.633)	***0.018***	1.287 (0.866–1.913)	0.211	1.267 (0.865–1.854)	0.224	1.264 (0.873–1.830)	0.214	1.488 (1.011–2.189)	***0.044***
LINC00271 (low *vs* high)	2.655 (1.109–6.359)	***0.028***	2.523 (1.673–3.805)	**<*****0.001***	1.753 (1.193–2.574)	***0.004***	2.377 (1.628–3.472)	**<*****0.001***	2.464 (1.656–3.665)	**<*****0.001***
HAR1A (low *vs* high)	2.699 (1.077–6.761)	***0.034***	1.627 (1.091–2.426)	***0.017***	1.682 (1.146–2.468)	***0.008***	1.377 (0.951–1.994)	0.090	1.17 (00.890–1.922)	0.171
LINC00310 (low *vs* high)	2.494 (1.041–5.972)	***0.040***	1.898 (1.274–2.830)	***0.002***	1.552 (1.077–2.236)	***0.018***	1.587 (1.092–2.308)	***0.016***	1.471 (0.996–2.173)	0.052
HAS2-AS1 (low *vs* high)	2.389 (1.030–5.541)	***0.043***	0.633 (0.425–0.942)	***0.024***	0.560 (0.381–0.822)	***0.003***	0.726 (0.501–1.052)	0.090	0.67 (0.457–0.989)	***0.044***

Notes: Italic and bold type indicates statistical significance; Abbreviations: lncRNA, long non-coding RNA; PTC, papillary thyroid cancer; TCGA, The Cancer Genomics Atlas; RFS, recurrence free survival; HR, hazards ratio; OR, odds ratio; CI, confidence interval; ETE, extrathyroidal extension; LNM, lymph node metastasis; TNM, tumor-node-metastasis.

**Table 3:** Multivariate analyses of associations of LINC00271 with high-risk clinicopathogical factors in the TCGA and FUSCC cohorts.

Variables	TCGA			FUSCC		
	*P* value^a^	OR	95.0% CI for OR	*P* value^a^	OR	95.0% CI for OR
ETE^b^						
LINC00271 ≥cutoff		1			1	
<cutoff^c^	***0.003***	2.299	1.324–3.992	0.107	2.506	0.820–7.663
LNM^d^						
LINC00271 ≥cutoff		1			1	
<cutoff^e^	***0.037***	1.713	1.033–2.841	***0.007***	2.554	1.300–5.018
III/IV stage^f^						
LINC00271 ≥cutoff		1			1	
<cutoff^g^	***0.011***	2.180	1.200–3.959	0.608	1.297	0.480–3.502

Notes: ^a^Italic and bold type indicates statistical significance; ^b^adjusted by age, gender, multifocality, histological subtypes, HT, LNM and *BRAF*^*V600E*^ mutation in multivariate analysis; ^c^LINC00271 expression level less than the best cutoff value for prediction of ETE; ^d^adjusted by age, gender, multifocality, histological subtypes, HT, ETE, T stage and *BRAF*^*V600E*^ mutation in multivariate analysis; ^f^LINC00271 expression level less than the best cutoff value for prediction of LNM; ^f^adjusted by gender, multifocality, histological subtypes, HT, ETE, LNM, T stage and *BRAF*^*V600E*^ mutation in multivariate analysis; ^g^LINC00271 expression level less than the best cutoff value for prediction of III/IV stage; Abbreviations: PTC, papillary thyroid cancer; TCGA, The Cancer Genomics Atlas; FUSCC, Fudan University Shanghai Cancer Center; OR, odds ratio; CI, confidence interval; HT, Hashimoto’s thyroiditis; ETE, extrathyroidal extension; LNM, lymph node metastasis; TNM, tumor-node-metastasis.

**Table 4:** Cox proportional hazards analysis of factors associated with RFS for PTC patients in TCGA cohort.

Variables	Univariate analysis			Multivariate analysis		
	*P* value^a^	HR	95.0% CI for HR	*P* value^a^	HR	95.0% CI for HR
Age^b^	*0.195*	1.016	0.992–1.040	0.977	1.000	0.968–1.034
Gender^b^						
Male		1			1	
Female	*0.009*	0.350	0.159–0.769	*0.021*	0.386	0.172–0.868
Multifocality						
Unifocal		1				
Multifocal	0.106	1.933	0.868–4.300			
Histological subtypes^b^						
Classical PTC		1			1	
Follicular PTC	0.913	0.941	0.318–2.786	0.871	1.095	0.367–3.269
Tall-cell PTC	0.202	2.229	0.650–7.641	0.886	1.098	0.306–3.949
Coexistent HT						
No		1				
Yes	0.740	1.729	0.648–4.616			
ETE						
Yes		1				
No	0.182	1.712	0.777–3.773			
T stage^b^						
T1-T2		1			1	
T3-T4	*0.047*	2.294	1.010–5.211	0.508	1.401	0.516–3.806
LNM						
No		1				
Yes	0.111	2.077	0.846–5.100			
TNM stage^b^						
Stage I-II		1			1	
Stage III-IV	0.052	2.176	0.992–4.773	0.664	1.326	0.371–4.738
*BRAF*^*V600E*^						
Wild-type		1				
Mutation	0.365	1.557	0.597–4.057			
LINC00271^b^						
≥cutoff		1			1	
<cutoff^c^	*0.009*	3.688	1.384–9.829	*0.025*	3.182	1.160–8.726

Notes: ^a^Italic and bold type indicates statistical significance; ^b^adjusted by age, gender, histological subtypes, T stage, TNM stage and LINC00271 in multivariate analysis; c, LINC00271 expression level less than the best cutoff value for prediction of recurrence; Abbreviations: PTC, papillary thyroid cancer; TCGA, The Cancer Genomics Atlas; OR, odds ratio; CI, confidence interval; HT, Hashimoto’s thyroiditis; ETE, extrathyroidal extension; LNM, lymph node metastasis; TNM, tumor-node-metastasis.
