# Corrigendum: Long intergenic non-coding RNA 271 is predictive of a poorer prognosis of papillary thyroid cancer

**DOI:** 10.1038/srep42321

**Published:** 2017-02-09

**Authors:** Ben Ma, Tian Liao, Duo Wen, Chuanpeng Dong, Li Zhou, Shuwen Yang, Yu Wang, Qinghai Ji

Scientific Reports 6: Article number: 3697310.1038/srep36973; published online: 11 11 2016; updated: 02 09 2017

This Article contains errors in Table 1 and Table 4. The correct Table 1 and Table 4 appear below as Table 1 and Table 2 respectively.

## Figures and Tables

**Table 1:** Clinicopathological characteristics of PTC patients in the FUSCC and TCGA cohorts.

Variables	TCGA (*N* = 471)		FUSCC (*N* = 185)		*P* value
	*N*	%	*N*	%	
Age (years)					0.099
<45	217	46.1	99	53.5	
≥45	254	53.9	86	46.5	
Gender					0.374
Male	126	26.8	43	23.2	
Female	345	73.2	142	76.8	
Multifocality					**<*****0.001***
Unifocal	250	54.2	135	73.0	
Multifocal	211	45.8	50	27.0	
Histological type					**<*****0.001***
Classical PTC	329	70.9	183	98.9	
Follicular PTC	99	21.3	2	1.1	
Tall-cell PTC	36	7.8	0	0	
Coexistent HT					***0.004***
Yes	66	14.0	44	23.8	
No	405	86.0	141	76.2	
ETE					**<*****0.001***
No	313	66.5	168	90.8	
Yes	144	30.6	17	9.2	
NA	14	3.0	0	0	
T Stage					**<*****0.001***
T1-T2	282	59.9	166	89.7	
T3-T4	188	39.9	19	10.3	
NA	1	0.2	0	0	
LNM					**<*****0.001***
N0	215	45.6	85	45.9	
N1	210	44.6	100	54.1	
NA	46	9.8	0	0	
M					**<*****0.001***
M0	263	55.8	185	100	
M1	8	1.7	0	0	
NA	200	42.5	0	0	
TNM Stage					**<*****0.001***
I-II	312	66.2	152	82.1	
III-IV	157	33.3	33	17.9	
NA	2	0.4	0	0	
*BRAF*^***V600E***^					**<*****0.001***
Mutation	222	47.1	103	55.7	
Wild-type	177	37.6	79	42.7	
NA	72	15.3	3	1.6	

Notes: Italic and bold type indicates statistical significance. Abbreviations: PTC, papillary thyroid cancer; FUSCC, Fudan University Shanghai Cancer Center; TCGA, The Cancer Genomics Atlas; HT, Hashimoto’s thyroiditis; ETE, extrathyroidal extension; LNM, lymph node metastasis; M, metastasis; TNM, tumor-node-metastasis.

**Table 2:** Cox proportional hazards analysis of factors associated with RFS for PTC patients in TCGA cohort.

Variables	Univariate analysis			Multivariate analysis		
	*P* value^a^	HR	95.0% CI for HR	*P* value^a^	HR	95.0% CI for HR
Age^b^	*0.195*	1.016	0.992–1.040	0.977	1.000	0.968–1.034
Gender^b^						
Male		1			1	
Female	***0.009***	0.350	0.159–0.769	***0.021***	0.386	0.172–0.868
Multifocality						
Unifocal		1				
Multifocal	0.106	1.933	0.868–4.300			
Histological subtypes^b^						
Classical PTC		1			1	
Follicular PTC	0.913	0.941	0.318–2.786	0.871	1.095	0.367–3.269
Tall-cell PTC	0.202	2.229	0.650–7.641	0.886	1.098	0.306–3.949
Coexistent HT						
No		1				
Yes	0.740	1.729	0.648–4.616			
ETE						
No		1				
Yes	0.182	1.712	0.777–3.773			
T stage^b^						
T1-T2		1			1	
T3-T4	***0.047***	2.294	1.010–5.211	0.508	1.401	0.516–3.806
LNM						
No		1				
Yes	0.111	2.077	0.846–5.100			
TNM stage^b^						
Stage I-II		1			1	
Stage III-IV	0.052	2.176	0.992–4.773	0.664	1.326	0.371–4.738
*BRAF*^*V600E*^						
Wild-type		1				
Mutation	0.365	1.557	0.597–4.057			
LINC00271^b^						
≥cutoff		1			1	
<cutoff^c^	***0.009***	3.688	1.384–9.829	*0.025*	3.182	1.160–8.726

Notes: ^a^Italic and bold type indicates statistical significance; ^b^adjusted by age, gender, histological subtypes, T stage, TNM stage and LINC00271 in multivariate analysis; ^c^, LINC00271 expression level less than the best cutoff value for prediction of recurrence; Abbreviations: PTC, papillary thyroid cancer; TCGA, The Cancer Genomics Atlas; OR, odds ratio; CI, confidence interval; HT, Hashimoto’s thyroiditis; ETE, extrathyroidal extension; LNM, lymph node metastasis; TNM, tumor-node-metastasis.

