# Supplementary material for: Long intergenic non-coding RNA 271 is predictive of a poorer prognosis of papillary thyroid cancer
Source: Sci Rep. 2016 Nov 11;6:36973. doi: 10.1038/srep36973 (PMC5105055; doi:10.1038/srep36973)
Supplement: Supplementary Information [file srep36973-s1.pdf]

Title：Long intergenic non-coding RNA 271 is predictive of a poorer prognosis of papillary thyroid cancer

Author names: Ben Ma[1,2], Tian Liao[1,2], Duo Wen[1,2], Chuanpeng Dong[3], Li Zhou[1,2], Shuwen Yang[1,2], Yu Wang[1,2], Qinghai Ji[1,2]

Table S1 The annotated 2773 lncRNAs from the website of HGNC

HGNC ID	Approved Symbol	Chromosome	Accession Numbers	Entrez Gene ID	Ensembl Gene ID	Pubmed IDs	RefSeq IDs
HGNC:37133	A1BG-AS1	19q13.43	BC040926	503538	ENSG00000268895		NR_015380
HGNC:27057	A2M-AS1	12p13.31		144571	ENSG00000245105		NR_026971
HGNC:41022	A2ML1-AS1	12p13.31			ENSG00000256661		
HGNC:41523	A2ML1-AS2	12p13.31			ENSG00000256904		
HGNC:50301	AADACL2-AS1	3q25.1		101928142	ENSG00000242908		
HGNC:51526	AATBC	21q22.3		284837	ENSG00000215458	25473900	NR_026961
HGNC:40053	AATK-AS1	17q25.3		388428	ENSG00000225180		
HGNC:49667	ABALON	20q11.21	KC505631	103021294		24992962	NR_131907
HGNC:39983	ABCA9-AS1	17q24.2			ENSG00000231749		
HGNC:40055	ABCC5-AS1	3q27.1			ENSG00000223882		
HGNC:18289	ABHD11-AS1	7q11.23	BU595969	171022	ENSG00000225969	24984296, 25619660	NR_026690
HGNC:49685	ABHD15-AS1	17q11.2	CN275323		ENSG00000264031	24504737	
HGNC:41426	ACAP2-IT1	3q29			ENSG00000229325		
HGNC:45169	ACTA2-AS1	10q23.31	AK093340, AX748062	100132116	ENSG00000180139	24721325	
HGNC:20131	ACTN1-AS1	14q24.1	DB029126, AI147349	161159	ENSG00000259062		
HGNC:44161	ACVR2B-AS1	3p22.2	AK025619	100128640	ENSG00000229589		NR_028389
HGNC:40625	ADAMTS9-AS1	3p14.1		101929335	ENSG00000241158		
HGNC:42435	ADAMTS9-AS2	3p14.1		100507098	ENSG00000241684	24833086	
HGNC:40797	ADAMTS19-AS1	5q23.3	DA728271		ENSG00000249421		
HGNC:32041	ADAMTSL4-AS1	1q21.2	AK127688		ENSG00000203804		
HGNC:23299	ADARB2-AS1	10p15.3		642394	ENSG00000205696		NR_033387
HGNC:48682	ADD3-AS1	10q25.1		100505933			
HGNC:49543	ADGRA1-AS1	10q26.3			ENSG00000256925		
HGNC:50604	ADGRL3-AS1	4q13.1		101927186	ENSG00000248692		
HGNC:40648	ADIPOQ-AS1	3q27.3		100874095	ENSG00000226482		NR_046662
HGNC:45127	ADIRF-AS1	10q23.2	AK128041	100133190	ENSG00000272734		
HGNC:51227	ADNP-AS1	20q13.13		101927631	ENSG00000259456		NR_110009
HGNC:37122	ADORA2A-AS1	22q11.23	AK091970	646023		19726446	NR_028483
HGNC:44144	ADPGK-AS1	15q24.1	AK127877	100287559	ENSG00000260898		NR_040107
HGNC:28141	AFAP1-AS1	4p16.1	BC004397	84740		23333711	NM_032654
HGNC:41334	AFF2-IT1	Xq28			ENSG00000223516		
HGNC:41427	AGAP1-IT1	2q37.2		100506749	ENSG00000235529		
HGNC:48633	AGAP2-AS1	12q14.1	BC039697, BC069024	100130776	ENSG00000255737	25392693	NR_027032
HGNC:48617	AGBL1-AS1	15q25.3		727915	ENSG00000260125		
HGNC:41482	AGBL4-IT1	1p33			ENSG00000225623		
HGNC:41133	AGBL5-AS1	2p23.3			ENSG00000231636		
HGNC:41484	AGBL5-IT1	2p23.3			ENSG00000229122		
HGNC:20988	AGPAT4-IT1	6q25.3	AK026765	79992			NR_024277
HGNC:34515	AIRN	6q25.3		100271873	ENSG00000268257	18789384, 19008856	
HGNC:41304	AKT3-IT1	1q44			ENSG00000228939		
HGNC:40244	ALDH1L1-AS1	3q21.3			ENSG00000250218		
HGNC:42446	ALDH1L1-AS2	3q21.3			ENSG00000246022		
HGNC:41409	ALG9-IT1	11q23.1			ENSG00000254450		
HGNC:41277	ALG13-AS1	Xq23			ENSG00000229487		
HGNC:48670	ALKBH3-AS1	11p11.2		100507300	ENSG00000244926		
HGNC:41305	ALMS1-IT1	2p13.1	BC041487	100874291	ENSG00000230002		NR_046762
HGNC:51342	ALOX12-AS1	17p13.1	AK074580	100506713	ENSG00000215067		NR_040089
HGNC:41306	AMMECR1-IT1	Xq23			ENSG00000224142		
HGNC:39891	ANKRD10-IT1	13q34		100505494	ENSG00000229152		
HGNC:41298	ANKRD33B-AS1	5p15.2			ENSG00000250106		
HGNC:48618	ANKRD34C-AS1	15q25.1	DA336675, DA364073	729911	ENSG00000259234		NR_038997

HGNC:41477	ANKRD44-IT1	2q33.1		101927547	ENSG00000236977		
HGNC:40016	ANO1-AS1	11q13.3			ENSG00000254902		
HGNC:33275	ANO1-AS2	11q13.3	AW592641, EF419887	100009613	ENSG00000254417		
HGNC:25672	ANP32A-IT1	15q23	AK021784	80035		14702039	NR_026808
HGNC:41307	AOAH-IT1	7p14.2		100874264	ENSG00000230539		
HGNC:44114	AP4B1-AS1	1p13.2	AK123199	100287722	ENSG00000226167		NR_037864
HGNC:27152	APCDD1L-AS1	20q13.32	AI077602, AK054637	149773	ENSG00000231290		NR_034147
HGNC:40079	APOA1-AS	11q23.3	DA327409		ENSG00000235910	24388749	
HGNC:43836	APOBEC3B-AS1	22q13.1	CX754295		ENSG00000249310		
HGNC:44173	APTR	7q11.23	AF290475	100505854	ENSG00000214293	24748121	NR_038361
HGNC:26399	AQP4-AS1	18q12.1	AK055069	147429	ENSG00000260372		
HGNC:39993	ARAP1-AS1	11q13.4			ENSG00000256007		
HGNC:39994	ARAP1-AS2	11q13.4		100506020	ENSG00000245148		
HGNC:51593	ARF4-AS1	3p14.3	BF793425, AI127075		ENSG00000272146		
HGNC:20279	ARHGAP5-AS1	14q12	BC007251	84837	ENSG00000258655		NR_027263
HGNC:42944	ARHGAP22-IT1	10q11.22	AK093071	100689073	ENSG00000248682		
HGNC:40792	ARHGAP26-AS1	5q31.3			ENSG00000226272		
HGNC:41429	ARHGAP26-IT1	5q31.3			ENSG00000230789		
HGNC:41235	ARHGAP31-AS1	3q13.33			ENSG00000241155		
HGNC:40083	ARHGEF3-AS1	3p14.3		100874200	ENSG00000240198		
HGNC:39816	ARHGEF7-AS1	13q34			ENSG00000227352		
HGNC:40717	ARHGEF7-AS2	13q34			ENSG00000235875		
HGNC:41408	ARHGEF7-IT1	13q34			ENSG00000233644		
HGNC:41401	ARHGEF9-IT1	Xq11.1			ENSG00000231729		
HGNC:39996	ARHGEF19-AS1	1p36.13			ENSG00000234166		
HGNC:41048	ARHGEF26-AS1	3q25.2		100507524	ENSG00000243069		
HGNC:41483	ARHGEF38-IT1	4q24			ENSG00000249885		
HGNC:41407	ARID4B-IT1	1q42.3					
HGNC:41008	ARMC2-AS1	6q21	BG504478		ENSG00000230290		
HGNC:41038	ARMCX3-AS1	Xq22.1		100128574	ENSG00000228275		
HGNC:49892	ARNTL2-AS1	12p11.23		101928646	ENSG00000245311		
HGNC:40785	ARPP21-AS1	3p22.3			ENSG00000230830		
HGNC:23395	ARRDC1-AS1	9q34.3	AF267857	85026	ENSG00000203993		NR_122035
HGNC:44145	ARRDC3-AS1	5q14.3	BC043415	100129716			NR_027435
HGNC:40085	ARSD-AS1	Xp22.33		100506356	ENSG00000229851		
HGNC:24998	ASAP1-IT1	8q24.21	AF161539	29065		11042152	NR_002765
HGNC:48917	ASAP1-IT2	8q24.21		100507117			NR_045385
HGNC:25442	ASB16-AS1	17q21.31	AL833953	339201	ENSG00000267080		NR_049729
HGNC:44146	ASH1L-AS1	1q22	BC017347	645676	ENSG00000235919		NR_027023
HGNC:41449	ASH1L-IT1	1q22			ENSG00000227773		
HGNC:25811	ASMTL-AS1	Xp22.33 and	AK023392	80161	ENSG00000236017	14702039	XR_040863
HGNC:51175	ASTN2-AS1	9q33.1	AK097822	100128505	ENSG00000229105		NR_033973
HGNC:49496	ATE1-AS1	10q26.13		100130887	ENSG00000226864		NR_120495
HGNC:40914	ATG10-AS1	5q14.1			ENSG00000248192		
HGNC:41455	ATG10-IT1	5q14.1			ENSG00000236447		
HGNC:28262	ATP1A1-AS1	1p13.1	BC007373	84852	ENSG00000203865	12477932	NR_024124
HGNC:40088	ATP1B3-AS1	3q23			ENSG00000244124		
HGNC:51370	ATP2A1-AS1	16p11.2	BX099641, AI015924	100289092	ENSG00000260442		NR_046287
HGNC:41309	ATP2B2-IT1	3p25.3			ENSG00000236999		
HGNC:41310	ATP2B2-IT2	3p25.3			ENSG00000224771		
HGNC:51118	ATP6V1B1-AS1	2p13.3		101927750	ENSG00000239322		
HGNC:44180	ATP6V0E2-AS1	7q36.1	BC028735	401431	ENSG00000204934		NR_027040
HGNC:40645	ATP11A-AS1	13q34			ENSG00000232684		
HGNC:41095	ATP13A4-AS1	3q29		101929198	ENSG00000225473		

HGNC:41281	ATP13A5-AS1	3q29			ENSG00000236508		
HGNC:51838	ATXN2-AS	12q24.12		102723619	ENSG00000258099		
HGNC:10561	ATXN8OS	13q21	AF126749	6315	ENSG00000230223	10192387, 16804541	NR_002717
HGNC:51558	AZIN1-AS1	8q22.3		100506753	ENSG00000253320		NR_126338
HGNC:16424	B3GALT5-AS1	21q22.2	AF426266	114041	ENSG00000184809	12036297	NR_026542
HGNC:49910	B4GALT1-AS1	9p21.1		101929639	ENSG00000233554	26315939	NR_108108
HGNC:40090	B4GALT4-AS1	3q13.33			ENSG00000240254		
HGNC:50461	BAALC-AS1	8q22.3		100499183	ENSG00000247081		
HGNC:28595	BAALC-AS2	8q22.3	BC029562	157556	ENSG00000236939	12477932	NR_027071
HGNC:37125	BACE1-AS	11q23.3		100379571	ENSG00000278768	18587408, 21785702	NR_037803
HGNC:16024	BACE2-IT1	21q22.2		282569	ENSG00000224388		
HGNC:40008	BACH1-AS1	21q21.3			ENSG00000232118		
HGNC:40006	BACH1-IT1	21q21.3	AF317902		ENSG00000248476		
HGNC:40007	BACH1-IT2	21q21.3			ENSG00000228817		
HGNC:16455	BACH1-IT3	21q21.3	BX100223	54147	ENSG00000234293		
HGNC:44342	BAIAP2-AS1	17q25.3	AK027350, AK056555,	440465	ENSG00000226137		NR_026857
HGNC:43877	BANCR	9q21.11	AA442164	100885775		22581800, 22673151,	NR_047671
HGNC:50673	BARX1-AS1	9q22.32		101928040	ENSG00000235601		
HGNC:50700	BBOX1-AS1	11p14.2	DA865512		ENSG00000254560		
HGNC:22170	BCAR4	16p13.13		400500	ENSG00000262117	16778085, 23202989	
HGNC:44113	BCDIN3D-AS1	12q13.12	BC014925	100286844	ENSG00000258057		NR_027499
HGNC:1022	BCYRN1	2p16	AF020057	618	ENSG00000236824	9858834, 8265590, 17	NR_001568
HGNC:20608	BDNF-AS	11p14.1	AY054391	497258	ENSG00000245573	15666411, 22446693	NR_002832
HGNC:51114	BEAN1-AS1	16q21		101927726	ENSG00000261656		
HGNC:28425	BFSP2-AS1	3q22.1		85003	ENSG00000249993	12477932	
HGNC:49033	BGLT3	11p15.4	AY034471, KF110790	103344929	ENSG00000260629	24837367	
HGNC:44471	BHLHE40-AS1	3p26.1	AK056892, AK311646	100507582	ENSG00000235831		
HGNC:25868	BIN3-IT1	8p21.3	AK024169, DN996902	80094		12477932	
HGNC:40641	BIRC6-AS1	2p22.3			ENSG00000230046	22196729	
HGNC:50490	BIRC6-AS2	2p22.3	AA883483, BX093813			22196729	
HGNC:51290	BISPR	19p13.11		105221694	ENSG00000269640	25620967	NR_130765
HGNC:48597	BLACAT1	1q32.1	BX640973	101669762		23688781	
HGNC:40096	BMP7-AS1	20q13.31		102723590	ENSG00000235032		NR_110631
HGNC:50864	BMPR1B-AS1	4q22.3		100507012	ENSG00000249599		
HGNC:35125	BOK-AS1	2q37.3		100379249	ENSG00000234235	19287972	NR_033346
HGNC:42922	BOLA3-AS1	2p13.1	BC030102, AK123453	100507171	ENSG00000225439		NR_045634
HGNC:13228	BPESC1	3q23	AF196865	60467	ENSG00000232416	10995571	NR_026783
HGNC:41311	BRD2-IT1	6p21.32			ENSG00000223837		
HGNC:44171	BRE-AS1	2p23.2	BC014088	100302650			NR_028308
HGNC:40614	BRWD1-AS1	21q22.2			ENSG00000238141		
HGNC:16423	BRWD1-AS2	21q22.2	AF426265	103091865	ENSG00000255568	12036297	
HGNC:41920	BRWD1-IT1	21q22.2			ENSG00000237373		
HGNC:40102	BSN-AS1	3p21.31			ENSG00000235120		
HGNC:42445	BSN-AS2	3p21.31		100132677	ENSG00000226913		NR_038866
HGNC:40959	BTBD9-AS1	6p21.2		100505567	ENSG00000226533		
HGNC:21223	BVES-AS1	6q21	AL137368	154442	ENSG00000203808		NR_037157
HGNC:44148	BZRAP1-AS1	17q22	AK090885	100506779			NR_038410
HGNC:44351	C1QTNF1-AS1	17q25.3		100507410	ENSG00000265096		
HGNC:39906	C1QTNF9-AS1	13q12.12			ENSG00000240868		
HGNC:39839	C1QTNF9B-AS1	13q12.12		542767	ENSG00000205861	15930275	
HGNC:27461	C1RL-AS1	12p13.31	AL833364	283314	ENSG00000205885		NR_026947
HGNC:49464	C2-AS1	6p21.3	BX100594	102060414			NR_104191
HGNC:41063	C3orf67-AS1	3p14.2	AK090895	101929238	ENSG00000242428		
HGNC:39753	C4A-AS1	6p21.33			ENSG00000233627		

HGNC:39752	C4B-AS1	6p21.33			ENSG00000229776		
HGNC:49679	C5orf66-AS1	5q31.1		101927953	ENSG00000249082	26158411	NR_105049
HGNC:27964	C5orf66-AS2	5q31.1		340073	ENSG00000249647	12477932	
HGNC:39767	C6orf47-AS1	6p21.33			ENSG00000227198		
HGNC:27840	C8orf34-AS1	8q13.2	BC041480	286189	ENSG00000248801	12477932	NR_038877
HGNC:50444	C8orf37-AS1	8q22.1		100616530	ENSG00000253773		
HGNC:51188	C9orf41-AS1	9q21.13	BC043649	101927380	ENSG00000203321		NR_121183
HGNC:48713	C9orf135-AS1	9q21.12		494558	ENSG00000225626		
HGNC:51176	C9orf173-AS1	9q34.3	AK097764	100129722	ENSG00000275549		NR_038389
HGNC:45007	C10orf71-AS1	10q11.23	AL598801, AL598689,	100506769	ENSG00000236208		
HGNC:51422	C15orf59-AS1	15q24.1		101929221	ENSG00000260469		NR_120352
HGNC:26393	C20orf166-AS1	20q13.33	AK054875	253868	ENSG00000174403		NR_033263
HGNC:1290	C21orf62-AS1	21q22.11		54067	ENSG00000205930		NR_024622
HGNC:16729	C21orf91-OT1	21q21.1	AY063452, AY063453	246312	ENSG00000240770	12036297	
HGNC:51657	CA3-AS1	8q21.2		100996348	ENSG00000253549		NR_121630
HGNC:40119	CACNA1C-AS1	12p13.33			ENSG00000246627		
HGNC:40118	CACNA1C-AS2	12p13.33			ENSG00000256271		
HGNC:40117	CACNA1C-AS3	12p13.33			ENSG00000256769		
HGNC:40116	CACNA1C-AS4	12p13.33			ENSG00000256025		
HGNC:41312	CACNA1C-IT1	12p13.33			ENSG00000256837		
HGNC:41313	CACNA1C-IT2	12p13.33			ENSG00000256257		
HGNC:41314	CACNA1C-IT3	12p13.33			ENSG00000256721		
HGNC:27377	CACNA1G-AS1	17q21.33		253962	ENSG00000250107	26397149	NR_038439
HGNC:40702	CACNA2D3-AS1	3p14.3			ENSG00000243715		
HGNC:31391	CACTIN-AS1	19p13.3	AK097999	404665	ENSG00000226800		NR_038865
HGNC:41248	CADM2-AS1	3p12.1			ENSG00000239519		
HGNC:41247	CADM2-AS2	3p12.1			ENSG00000241648		
HGNC:40812	CADM3-AS1	1q23.2		100131825	ENSG00000225670		
HGNC:42860	CAHM	6q26	BG168587	100526820		24799664	NR_037593
HGNC:44682	CALML3-AS1	10p15.1	DA220455, DA770774	100132159	ENSG00000205488		
HGNC:41446	CAMTA1-IT1	1p36.23			ENSG00000237402		
HGNC:48839	CAPN10-AS1	2q37.3	AK024254	101752400	ENSG00000260942	23382218	
HGNC:51408	CARD8-AS1	19q13.33		100505812	ENSG00000268001	25392693	NR_040599
HGNC:42872	CARMN	5q32		728264	ENSG00000249669	26423156	NR_105059
HGNC:40125	CARS-AS1	11p15.4	AK093033		ENSG00000247473		
HGNC:22933	CASC2	10q26.12	AJ535620	255082	ENSG00000177640		
HGNC:49076	CASC6	6q15	BC037927		ENSG00000224944		
HGNC:48905	CASC7	8q24.3	AK092172, BC033700	286109	ENSG00000259758		
HGNC:45129	CASC8	8q24.21		727677	ENSG00000246228	24594601	NR_024393
HGNC:48906	CASC9	8q21.11	BC062758	101805492	ENSG00000249395	25885227	
HGNC:48939	CASC11	8q24.21	BC042052	100270680	ENSG00000249375	24594601	
HGNC:28245	CASC15	6p22.3	AK026189	401237	ENSG00000272168	26016895, 26100672	
HGNC:48608	CASC16	16q12.1	BC034767, DB462404,	643714	ENSG00000249231		NR_033920
HGNC:43911	CASC17	17q24.3	BC039327		ENSG00000260785		
HGNC:49463	CASC18	12q23.3	AK001551	101929110	ENSG00000257859		
HGNC:49476	CASC19	8q24.21	BI223175, JX003871, D	103021165	ENSG00000254166	24594601	
HGNC:49477	CASC20	20p12.3	AY007089	101929244	ENSG00000229876		
HGNC:49836	CASC21	8q24.21	JX003867, CB104826	103021164	ENSG00000253929	24594601	
HGNC:50627	CASC22	16q12.1	AK093211	283854		24879036	
HGNC:50865	CASC23	11p15.4	BC027619	103581031	ENSG00000255420		
HGNC:40126	CASK-AS1	Xp11.4			ENSG00000233033		
HGNC:41080	CATIP-AS1	2q35	BC038211	101928513	ENSG00000225062		
HGNC:41079	CATIP-AS2	2q35	CD672221		ENSG00000237281		
HGNC:43664	CBR3-AS1	21q22.12	BC038671	100506428	ENSG00000236830	22264502, 24337686	NR_038892

HGNC:45128	CCAT1	8q24.21	HM358356, AK125310	100507056	ENSG00000247844	23416875, 23143645, 21547902, 245	
HGNC:47044	CCAT2	8q24.21	GQ911591	101805488		23796952, 24504682	
HGNC:41142	CCDC13-AS1	3p22.1			ENSG00000173811		
HGNC:52262	CCDC18-AS1	1p22.1		100131564	ENSG00000223745	26421281	NR_034089
HGNC:28416	CCDC26	8q24.21	BC026098	137196	ENSG00000229140	16449964, 23399484	
HGNC:49641	CCDC37-AS1	3q21.3		100506907	ENSG00000249833		
HGNC:41089	CCDC39-AS1	3q26.33			ENSG00000243187		
HGNC:51340	CCDC144NL-AS	17p11.2	AK057473	440416	ENSG00000233098		NR_104185
HGNC:44134	CCDC148-AS1	2q24.1	BC021739	554201	ENSG00000227480		NR_038850
HGNC:44105	CCDC183-AS1	9q34.3	AK055547	100131193	ENSG00000228544		NR_024580
HGNC:51693	CCEPR	10q21.1		105682749		25921283	NR_131782
HGNC:49398	CCND2-AS1	12p13.32	BG413373		ENSG00000256164		
HGNC:49399	CCND2-AS2	12p13.32	DA223008		ENSG00000255920		
HGNC:40130	CCNT2-AS1	2q21.3		100129961	ENSG00000224043		
HGNC:43896	CD27-AS1	12p13.31	AK056500	678655	ENSG00000215039		NR_015382
HGNC:49384	CD81-AS1	11p15.5	BC042822	101927682	ENSG00000238184		
HGNC:49735	CDC37L1-AS1	9p24.1	CK820219	101929351	ENSG00000273061		
HGNC:41317	CDC42-IT1	1p36.12			ENSG00000230068		
HGNC:31433	CDH23-AS1	10q22.1			ENSG00000223817		
HGNC:48609	CDIPT-AS1	16p11.2	AK131031	440356	ENSG00000214725		NR_015396
HGNC:42399	CDKN1A-AS1	6p21.1	BX332409	100861512		19008947	
HGNC:23831	CDKN2A-AS1	9p21.3	AF211119	51198	ENSG00000224854		NR_024274
HGNC:34341	CDKN2B-AS1	9p21.3	BC038540	100048912	ENSG00000240498	18048406, 17440112,	NR_003529
HGNC:48926	CDR1-AS	Xq27.1	AK054921	103611090		23446346, 23446348, 21964070	
HGNC:14386	CDRT7	17p12	BX093265, AI218510	94150	ENSG00000259944	11381029	
HGNC:14387	CDRT8	17p12	AI150443, CR737651, A	94151	ENSG00000265163	11381029	
HGNC:25710	CEBPA-AS1	19q13.11	AK022417, BC027959	80054	ENSG00000267296		NR_026887
HGNC:51226	CEBPB-AS1	20q13.13	BX463443	101927559	ENSG00000277449		NR_125739
HGNC:1841	CECR3	22q11.2	AF277398	27442	ENSG00000241832	11381032	
HGNC:1842	CECR5-AS1	22q11.2	AF307448	100130717	ENSG00000185837	11381032	NR_024483
HGNC:1845	CECR7	22q11.2	BC043198	100130418	ENSG00000237438	11381032	NR_015352
HGNC:1847	CECR9	22q11.2	AF307449	30847	ENSG00000231004	11381032	
HGNC:23515	CELF2-AS1	10p14	AK097813	414196	ENSG00000181800		XR_109007
HGNC:44703	CELF2-AS2	10p14		439950	ENSG00000237986		NR_046108
HGNC:50302	CELSR3-AS1	3p21.31	BM714397	102724368	ENSG00000228350		
HGNC:27055	CEP83-AS1	12q22		144486		12477932	NR_027035
HGNC:51431	CERS3-AS1	15q26.3	AL708976, AI160488	102723320	ENSG00000259430		NR_120374
HGNC:44485	CERS6-AS1	2q24.3		100861402	ENSG00000227617		NR_045786
HGNC:41113	CFAP44-AS1	3q13.2			ENSG00000243849		
HGNC:45243	CFAP58-AS1	10q25.1	BI551201, BX093559, [illegible]	100505869	ENSG00000231233		
HGNC:14437	CFLAR-AS1	2q33	AB053312	65072	ENSG00000226312		
HGNC:40146	CHKB-AS1	22q13.33		100144603	ENSG00000205559		
HGNC:40148	CHL1-AS1	3p26.3		101927193	ENSG00000234661		
HGNC:40147	CHL1-AS2	3p26.3		642891	ENSG00000224318		
HGNC:1279	CHODL-AS1	21q21.1		54075	ENSG00000231755		
HGNC:40150	CHRM3-AS1	1q43	BX116264, AI672277		ENSG00000234601		
HGNC:43725	CHRM3-AS2	1q43	AL832284, BI836697	100506915	ENSG00000233355		
HGNC:28588	CIRBP-AS1	19p13.3	BC026041	148046		12477932	NR_027271
HGNC:49426	CISTR	12q13.13	DA125942, HY042141	102216268		23093776	NR_104332
HGNC:48997	CKMT2-AS1	5q14.1		100131067	ENSG00000247572		
HGNC:39907	CLDN10-AS1	13q32.1			ENSG00000223392		
HGNC:48586	CLIP1-AS1	12q24.31		100507066	ENSG00000257097		
HGNC:30895	CLRN1-AS1	3q25.1		116933	ENSG00000239265	11524702	
HGNC:49095	CLSTN2-AS1	3q23	AL133103	101927808	ENSG00000250433		

HGNC:39893	CLYBL-AS1	13q32.3		101927465	ENSG00000234303		
HGNC:39894	CLYBL-AS2	13q32.3			ENSG00000227659		
HGNC:41031	CNOT10-AS1	3p22.3			ENSG00000251224		
HGNC:48712	CNTFR-AS1	9p13.3	BC038740, BC042806	415056	ENSG00000237159		
HGNC:39985	CNTN4-AS1	3p26.2			ENSG00000237990		
HGNC:39986	CNTN4-AS2	3p26.3			ENSG00000227588		
HGNC:40156	COL4A2-AS1	13q34			ENSG00000232814		
HGNC:39849	COL4A2-AS2	13q34		100129836	ENSG00000224821		
HGNC:31368	COL5A1-AS1	9q34.3	AI798815, CR748640		ENSG00000204011		
HGNC:23132	COL18A1-AS1	21q22.3	AK096071	378832	ENSG00000183535		NR_027498
HGNC:40155	COL18A1-AS2	21q22.3			ENSG00000224574		
HGNC:2238	COPG2IT1	7q32	AF038190	53844		10995575	NR_024086
HGNC:38873	COX10-AS1	17p12		100874058	ENSG00000236088		
HGNC:39898	CPB2-AS1	13q14.13		100509894	ENSG00000235903		
HGNC:27523	CPEB1-AS1	15q25.2	BC035360	283692	ENSG00000259462		NR_046096
HGNC:49082	CPEB2-AS1	4p15.33		441009	ENSG00000247624		
HGNC:30102	CPS1-IT1	2q34		29034		12477932	NR_002763
HGNC:26327	CRHR1-IT1	17q21.31	AK057897	147081			NR_026905
HGNC:37078	CRNDE	16q12.2		643911	ENSG00000245694	23226159, 24381249,	NR_034105
HGNC:51433	CRTC3-AS1	15q26.1	BG472948	101926895	ENSG00000259736		NR_120372
HGNC:34405	CRYM-AS1	16p12.2		400508	ENSG00000189149		NR_026675
HGNC:51232	CSE1L-AS1	20q13.13		102723483	ENSG00000227431		NR_110624
HGNC:40882	CSMD2-AS1	1p35.1		402779	ENSG00000231163		
HGNC:28604	CSNK1G2-AS1	19p13.3	BC029585	255193	ENSG00000180846	12477932	NR_033400
HGNC:27882	CSTF3-AS1	11p13		338739	ENSG00000247151	12477932	NR_034027
HGNC:48337	CTBP1-AS	4p16.3	AX747592, AK092548	285463		23644382, 25552498	
HGNC:28307	CTBP1-AS2	4p16.3	AK056133	92070	ENSG00000196810	12477932	NR_033339
HGNC:28543	CYP1B1-AS1	2p22.2	BC031410	285154	ENSG00000232973	12477932	
HGNC:43715	CYP4A22-AS1	1p33	BQ688560		ENSG00000225506	20887892	
HGNC:31671	CYP17A1-AS1	10q24.32		102724307	ENSG00000203886		
HGNC:50694	CYP51A1-AS1	7q21.2	BC042829	613126	ENSG00000188693	24905231	
HGNC:39560	CYYR1-AS1	21q21.3	AK125338		ENSG00000197934	24981926	
HGNC:49443	DAB1-AS1	1p32.2		101926890	ENSG00000226759		
HGNC:51902	DACOR1	15q22.33		100131796		26307088	
HGNC:44120	DACT3-AS1	19q13.32	BC043434	100506068	ENSG00000245598		NR_040041
HGNC:51429	DALIR	2q12	BE439524, DA337013, EB386236			25415054	
HGNC:28964	DANCR	4q12	BC015222	57291	ENSG00000226950	7788527, 22302877, 1	NR_024031
HGNC:50863	DANT1	Xq23	CD173052	106029310	ENSG00000229335	26188586	
HGNC:50862	DANT2	Xq23		642776	ENSG00000235244	26188586	NR_132337
HGNC:30243	DAOA-AS1	13q33.2	AY138548	282706	ENSG00000232307	12364586, 12647258	
HGNC:43591	DAPK1-IT1	9q21.33	BQ025444		ENSG00000236709	22078386	
HGNC:40170	DARS-AS1	2q21.3		101928243	ENSG00000231890		
HGNC:43904	DBET	4q35.2	JQ639078	100419743	ENSG00000281591	22541069	
HGNC:24155	DBH-AS1	9q34.2	AF129263	138948	ENSG00000225756	10686491, 26393879	NR_002783
HGNC:44151	DCTN1-AS1	2p13.1	BC042946	100189589	ENSG00000237737		NR_024463
HGNC:39889	DCUN1D2-AS	13q34	AI203167		ENSG00000233613		
HGNC:40171	DDC-AS1	7p12.1		100129427	ENSG00000226122		
HGNC:28694	DDR1-AS1	6p21.3	AL137624	414771	ENSG00000237775		
HGNC:44176	DDX11-AS1	12p11.21	AK096360	100506660	ENSG00000245614		NR_038927
HGNC:39771	DDX39B-AS1	6p21.33			ENSG00000234006		
HGNC:42517	DENND5B-AS1	12p11.21			ENSG00000255867		
HGNC:41152	DENND6A-AS1	3p14.3	AW183704		ENSG00000239801		
HGNC:50592	DEPDC1-AS1	1p31.2		101927220	ENSG00000234264		
HGNC:16757	DGCR5	22q11	X91348	26220	ENSG00000237517	8659529	NR_002733

HGNC:17227	DGCR9	22q11.21	L77571	25787		8776594	
HGNC:17864	DGCR10	22q11	L77559	26222		8776594	
HGNC:17226	DGCR11	22q11.21	L77561	25786		8776594	
HGNC:17225	DGCR12	22q11.21	L77562	25784		8776594	
HGNC:43441	DGUOK-AS1	2p13.1			ENSG00000237883		
HGNC:23175	DHRS4-AS1	14q11.2	AF116636	55449	ENSG00000215256	22891334	NR_023921
HGNC:38713	DHRSX-IT1	Xp22.33 and Yp11			ENSG00000223571		
HGNC:16972	DIAPH2-AS1	Xq31.33			ENSG00000236256	8133036, 7736777	
HGNC:39915	DIAPH3-AS1	13q21.2			ENSG00000227528		
HGNC:39916	DIAPH3-AS2	13q21.2			ENSG00000223815		
HGNC:43017	DICER1-AS1	14q32.13	BC040596	400242	ENSG00000235706		NR_015415
HGNC:44153	DIO2-AS1	14q31.1	BC039670	100628307	ENSG00000258766		NR_038355
HGNC:20348	DIO3OS	14q32.31	AF305836	64150	ENSG00000258498	14962667	NR_002770
HGNC:41430	DIP2A-IT1	21q22.3		100862692	ENSG00000223692		
HGNC:50636	DIRC3-AS1	2q35	CO245227		ENSG00000233143		
HGNC:41325	DISC1-IT1	1q42.2			ENSG00000226758		
HGNC:33625	DISC1FP1	11q14.3	AK130626, EU302123	101929222	ENSG00000261645		NR_104190
HGNC:2889	DISC2	1q42.1	AF222981	27184		10814723, 15478311	NR_002227
HGNC:13747	DLEU1	13q14.3	Y15227	10301	ENSG00000176124	9395242, 11406609	NR_002605
HGNC:50496	DLEU1-AS1	13q14.3	BM264234		ENSG00000229323		
HGNC:13748	DLEU2	13q14	Y15228	8847	ENSG00000231607	9395242, 11072235	NR_002612
HGNC:39966	DLEU7-AS1	13q14.3			ENSG00000237152		
HGNC:44154	DLG1-AS1	3q29	BC039329	100507086	ENSG00000227375		NR_038289
HGNC:37132	DLG2-AS1	11q14.1	AF525782	100302690		13130513	
HGNC:40182	DLG3-AS1	Xq13.1			ENSG00000231651		
HGNC:45109	DLG5-AS1	10q22.3		100128292	ENSG00000233871		NR_024585
HGNC:31676	DLGAP1-AS1	18p11.31		649446	ENSG00000177337		
HGNC:28146	DLGAP1-AS2	18p11.31	BC005130	84777	ENSG00000262001		
HGNC:27317	DLGAP1-AS3	18p11.31		201477	ENSG00000263724	12477932	
HGNC:44333	DLGAP1-AS4	18p11.31	BI755422, CK005798		ENSG00000263878		
HGNC:27586	DLGAP1-AS5	18p11.31		284215	ENSG00000261520		
HGNC:50467	DLGAP2-AS1	8p23.3		100507435	ENSG00000253267		
HGNC:51223	DLGAP4-AS1	20q11.23	BC039668	101926987	ENSG00000232907		NR_109939
HGNC:50638	DLX2-AS1	2q31.1	DB036501, AA625909		ENSG00000236651		
HGNC:37151	DLX6-AS1	7q21.3		285987	ENSG00000231764	16705037	NR_015448
HGNC:40183	DLX6-AS2	7q21.3		100873931			
HGNC:40185	DMD-AS3	Xp21.1			ENSG00000236828		
HGNC:48594	DNAH17-AS1	17q25.3		100996295	ENSG00000267432		
HGNC:49846	DNAJB5-AS1	9p13.3	DA679664	101926900			
HGNC:41029	DNAJB8-AS1	3q21.3		285224	ENSG00000242049		
HGNC:39808	DNAJC3-AS1	13q32.1		100289274	ENSG00000247400		
HGNC:31432	DNAJC9-AS1	10q22.2	BC043233	414245	ENSG00000236756		
HGNC:42943	DNAJC27-AS1	2p23.3		729723	ENSG00000224165		NR_034113
HGNC:41494	DNM3-IT1	1q24.3			ENSG00000233540		
HGNC:41228	DNM3OS	1q24.3		100628315	ENSG00000230630	19029138, 20400975	NR_038397
HGNC:20431	DNMBP-AS1	10q24.2	AK098111	100188954	ENSG00000227695		
HGNC:40876	DOCK4-AS1	7q31.1		100506413	ENSG00000225572		
HGNC:40672	DOCK9-AS1	13q32.3	AI027206		ENSG00000229918		
HGNC:43696	DOCK9-AS2	13q32.3	AF339771		ENSG00000260992		
HGNC:44147	DPH6-AS1	15q14	BC025664	100507466	ENSG00000248079		NR_038251
HGNC:50706	DPP9-AS1	19p13.3		100131094	ENSG00000205790		
HGNC:40941	DPP10-AS1	2q14.1		389023	ENSG00000235026		
HGNC:40940	DPP10-AS2	2q14.1			ENSG00000235717		
HGNC:40939	DPP10-AS3	2q14.1		101927591	ENSG00000231538		

HGNC:40195	DPYD-AS1	1p21.3			ENSG00000232878		
HGNC:40196	DPYD-AS2	1p21.3			ENSG00000235777		
HGNC:41326	DPYD-IT1	1p21.3			ENSG00000232542		
HGNC:27082	DRAIC	15q23	BC104435	145837	ENSG00000245750	25700553	NR_026979
HGNC:40197	DSCAM-AS1	21q22.2		100506492	ENSG00000235123		
HGNC:41327	DSCAM-IT1	21q22.2			ENSG00000233756		
HGNC:51116	DSCAS	18q12.1		101927698	ENSG00000265888		
HGNC:41328	DSCR4-IT1	21q22.13			ENSG00000223608		
HGNC:16301	DSCR9	21q22	AB066100	257203	ENSG00000230366	12168953	NR_026719
HGNC:16302	DSCR10	21q22.13	AB066291	259234	ENSG00000233316	12168953	NR_027695.1
HGNC:51115	DSG1-AS1	18q12.1		101927718	ENSG00000266729		
HGNC:51311	DSG2-AS1	18q12.1	BC042986	100652770	ENSG00000264859		NR_045216
HGNC:48569	DUBR	3q13.12		344595	ENSG00000243701	24886442, 25686699	NR_028301
HGNC:41329	E2F3-IT1	6p22.3			ENSG00000224707		
HGNC:42328	EAF1-AS1	3p25.1			ENSG00000249786		
HGNC:49045	EDNRB-AS1	13q22.3	BC031243	100505518	ENSG00000225579		NR_103853
HGNC:49501	EDRF1-AS1	10q26.13	AA868453, BX091673	101927983	ENSG00000236991		XR_246200
HGNC:39999	EFCAB6-AS1	22q13.2			ENSG00000223843		
HGNC:44108	EFCAB14-AS1	1p33	BX113937	100130197	ENSG00000228237		NR_038827
HGNC:41169	EGFLAM-AS1	5p13.1			ENSG00000249491		
HGNC:41168	EGFLAM-AS2	5p13.2		100506475	ENSG00000248572		
HGNC:41167	EGFLAM-AS3	5p13.2			ENSG00000249071		
HGNC:41166	EGFLAM-AS4	5p13.2	BC014585		ENSG00000248730		
HGNC:40207	EGFR-AS1	7p11.2		100507500	ENSG00000224057	26271667	NR_047551
HGNC:49077	EGLN3-AS1	14q13.1	AA147919		ENSG00000258897		
HGNC:37129	EGOT	3p26.1	BC039547	100126791	ENSG00000235947	17351112, 22303364	NR_004428
HGNC:51418	EHD4-AS1	15q15.1	CF887666, CF891641	101928363	ENSG00000259883		NR_120332
HGNC:44133	EHHADH-AS1	3q27.2	BC047598	339926	ENSG00000223358		NR_038990
HGNC:48714	EHMT1-IT1	9q34.3	AK127990, BX106199	643210			
HGNC:39751	EHMT2-AS1	6p21.33			ENSG00000237080		
HGNC:40208	EIF1AX-AS1	Xp22.12			ENSG00000225037		
HGNC:44555	EIF1B-AS1	3p22.1	AK309874, BC045736	440952			NR_033965
HGNC:40209	EIF2B5-AS1	3q27.1			ENSG00000230215		
HGNC:41330	EIF2B5-IT1	3q27.1			ENSG00000227509		
HGNC:48616	EIF3J-AS1	15q21.1		645212	ENSG00000179523		
HGNC:49511	ELDR	7p11.2	DB084610	102725541			
HGNC:39071	ELFN1-AS1	7p22.3	BX119057	101927125	ENSG00000236081	24707484	
HGNC:40765	ELMO1-AS1	7p14.2	DA058843		ENSG00000224101		
HGNC:44156	ELOVL2-AS1	6p24.2	BC038757	100506409	ENSG00000230314		NR_038962
HGNC:49223	EMC3-AS1	3p25.3		442075	ENSG00000180385		
HGNC:48331	EML2-AS1	19q13.32		100287177	ENSG00000267757		NR_130728
HGNC:18511	EMX2OS	10q26.11	AY117034	196047	ENSG00000229847	12573261	NR_002791
HGNC:40214	ENO1-AS1	1p36.23		100505975	ENSG00000230679		
HGNC:41331	ENO1-IT1	1p36.23			ENSG00000236269		
HGNC:42396	ENOX1-AS1	13q14.11			ENSG00000233821		
HGNC:42397	ENOX1-AS2	13q14.11			ENSG00000238189		
HGNC:45203	ENTPD1-AS1	10q24.1		728558	ENSG00000226688		NR_038444
HGNC:26710	ENTPD3-AS1	3p22.1		285266	ENSG00000223797	12477932	NR_040100
HGNC:50504	EP300-AS1	22q13.2		101927279	ENSG00000231993		
HGNC:30749	EPB41L4A-AS1	5q22.2	AB048207	114915	ENSG00000224032	9490301	NR_015370
HGNC:25643	EPB41L4A-AS2	5q22.2		54508	ENSG00000278921		
HGNC:27799	EPHA1-AS1	7q35	AL833583	285965	ENSG00000229153		NR_033897
HGNC:50602	EPHA5-AS1	4q13.1		100144602	ENSG00000250846		
HGNC:40849	EPN2-AS1	17p11.2		100874018	ENSG00000235397		NR_048576

HGNC:41445	EPN2-IT1	17p11.2		100874309			NR_046824
HGNC:1229	ERC2-IT1	3p14.3	U88965	711		9653645	NR_024615
HGNC:41431	ERI3-IT1	1p34.1			ENSG00000233602		
HGNC:49404	ERICD	8q24.3	BX099671			24168400	
HGNC:32290	ERICH1-AS1	8p23.3	BC022082, BC038783	619343	ENSG00000237647	26393913	
HGNC:41093	ERICH3-AS1	1p31.1	BX092187	101927320	ENSG00000234497		
HGNC:41205	ERICH6-AS1	3q25.1	AI146376, AA262424	101928085	ENSG00000240137		
HGNC:39079	ESRG	3p14.3		790952	ENSG00000265992	17803967, 21861197	NR_027122
HGNC:40222	ETV5-AS1	3q27.2			ENSG00000234197		
HGNC:40223	EVX1-AS	7p15.2	DB455833, DB260732		ENSG00000253405		
HGNC:26596	EWSAT1	15q23	AK091087	283673	ENSG00000212766	25401475	NR_026949
HGNC:25175	EXOC3-AS1	5p15.33	BC014011	116349	ENSG00000221990	12477932	NR_126522
HGNC:27985	EXTL3-AS1	8p21.1	BC043205	101929402	ENSG00000246339	12477932	
HGNC:40609	EZR-AS1	6q25.3	AI831881, BE552153		ENSG00000233893		
HGNC:40225	F10-AS1	13q34			ENSG00000231882		
HGNC:27725	F11-AS1	4q35.2	BC038717	285441	ENSG00000251165	12477932	
HGNC:43713	FALEC	1q21.3	AL713297	100874054	ENSG00000228126	20887892, 25203321	NR_051960
HGNC:41276	FAM3D-AS1	3p14.2	BG215913		ENSG00000244383	25904139	
HGNC:19370	FAM13A-AS1	4q22.1	AK126217	285512	ENSG00000248019	15234000	NR_002806
HGNC:23667	FAM27B	9q21.11		100133121	ENSG00000278763		NR_027422
HGNC:23668	FAM27C	9q13		100132948	ENSG00000154537		NR_027421
HGNC:32015	FAM27D1	9p11.2		724094	ENSG00000275493		XR_132873
HGNC:32013	FAM27E2	9p11.2		100289124	ENSG00000276135		XR_078687
HGNC:28655	FAM27E3	9q21.11		100131997	ENSG00000274026	12477932	NR_103833
HGNC:44415	FAM27E4	9q13		100132439	ENSG00000204805		
HGNC:32410	FAM27E5	17p11.2	BC031617	284123	ENSG00000178130		NR_028336
HGNC:23955	FAM41AY1	Yq11.221	BC042981	340618	ENSG00000224989	12477932	NR_028083
HGNC:37135	FAM41AY2	Yq11.222		100302526	ENSG00000226362		NR_028084
HGNC:27635	FAM41C	1p36.33	BC047940	284593	ENSG00000230368	12477932	NR_027055
HGNC:49499	FAM53B-AS1	10q26.13	BI457689, AA620732, A	101927944	ENSG00000233334		XR_246199
HGNC:30444	FAM66A	8p23.1		100133172	ENSG00000227888		NR_026789
HGNC:28890	FAM66B	8p23.1		100128890	ENSG00000215374		NR_027423
HGNC:21644	FAM66C	12p13.31		440078	ENSG00000226711		NR_026788
HGNC:24159	FAM66D	8p23.1		100132923	ENSG00000255052		NR_027425
HGNC:18735	FAM66E	8p23.1		100132103	ENSG00000225725		
HGNC:32029	FAM74A1	9p12		401507	ENSG00000215112		NR_026803
HGNC:32030	FAM74A2	9p12		653114			XR_036862
HGNC:32031	FAM74A3	9q21.11		728495	ENSG00000274355		NR_026801
HGNC:32032	FAM74A4	9q12		401508	ENSG00000274583		NR_026802
HGNC:34036	FAM74A6	9q12		653123	ENSG00000274516		NG_009678.1
HGNC:34037	FAM74A7	9p11.2		100996582	ENSG00000277260		XR_078406
HGNC:48658	FAM83A-AS1	8q24.13	AY211907	100131726	ENSG00000204949		NR_024479
HGNC:16113	FAM83C-AS1	20q11.22	AL121753	140846	ENSG00000235214		
HGNC:48720	FAM83H-AS1	8q24.3		100128338	ENSG00000203499	25864709	
HGNC:27233	FAM87A	8p23.3	BC037297	157693		12477932	
HGNC:32236	FAM87B	1p36.33	AK097327	400728	ENSG00000177757		
HGNC:28069	FAM95A	2q11.1	AL137712	90499			NR_038409
HGNC:32318	FAM95B1	9p11.2	AL833349	100133036	ENSG00000223839		NR_026759
HGNC:45272	FAM95C	9p13.1		100289137			NR_047651
HGNC:32368	FAM99A	11p15.5	AK124823	387742	ENSG00000205866		
HGNC:32369	FAM99B	11p15.5	CR627417	100132464	ENSG00000205865		
HGNC:32334	FAM138A	1p36.33		645520	ENSG00000237613	11779631, 15233989	NR_026818
HGNC:33582	FAM138B	2q13		654412	ENSG00000226516	11779631, 15233989	NR_026821
HGNC:32333	FAM138C	9p24.3		654835	ENSG00000218839	11779631, 15233989	NR_026822

HGNC:33583	FAM138D	12p13.33		677784	ENSG00000249054	11779631, 15233989	NR_026823
HGNC:32335	FAM138E	15q26.3		100124412	ENSG00000248893	11779631, 15233989	NR_026819
HGNC:33581	FAM138F	19p13.3		641702	ENSG00000282591	11779631, 15233989	NR_026820
HGNC:41503	FAM155A-IT1	13q33.3			ENSG00000227248		
HGNC:15548	FAM167A-AS1	8p23.1	AJ301563	83656	ENSG00000184608		NR_026814
HGNC:45006	FAM170B-AS1	10q11.23		100506733	ENSG00000234736		NR_038973
HGNC:20133	FAM181A-AS1	14q32.12		283592	ENSG00000258584		NR_027004
HGNC:27239	FAM201A	9p12	BC041970	158228	ENSG00000204860	12477932	NR_027294
HGNC:49446	FAM212B-AS1	1p13.2		100506343	ENSG00000227811		
HGNC:17505	FAM215A	17q21.31	AF143236	23591		10948432	NR_026770
HGNC:43639	FAM215B	17q21.31	BC044941		ENSG00000232300		
HGNC:28223	FAM222A-AS1	12q24.11		84983	ENSG00000255650	12477932	NR_026661
HGNC:30612	FAM223A	Xq28	AY168775	100132967	ENSG00000279245		NR_027401
HGNC:34048	FAM223B	Xq28		286967	ENSG00000272681		NR_027402
HGNC:37736	FAM224A	Yq11.222	AK026667	401630	ENSG00000233522		NR_002161
HGNC:37737	FAM224B	Yq11.221	AI819212	401629	ENSG00000230663		NR_002160
HGNC:27855	FAM225A	9q32	AL832752	286333	ENSG00000231528		NR_024366
HGNC:21865	FAM225B	9q32	BX647840	100128385	ENSG00000225684		NR_024376
HGNC:28544	FAM226A	Xq13	BC041962	203429		12477932	NR_026595
HGNC:31964	FAM226B	Xq13.1	BC025725	653687		16382448	NR_026594
HGNC:32943	FAM230B	22q11.21	BC039313, AK128837	642633	ENSG00000215498		NR_108107
HGNC:43915	FANK1-AS1	10q26.2	AW080472		ENSG00000233409		
HGNC:41485	FAR1-IT1	11p15.2			ENSG00000254791		
HGNC:40229	FARP1-AS1	13q32.2			ENSG00000231194		
HGNC:50479	FARSA-AS1	19p13.2	AW881480		ENSG00000266975		
HGNC:37128	FAS-AS1	10q24.1		100302740		15829500, 24811343	
HGNC:27557	FBXL19-AS1	16p11.2	AK090544	283932	ENSG00000260852		NR_024348
HGNC:51147	FBXO3-AS1	11p13		101928440	ENSG00000254508		
HGNC:31025	FBXO22-AS1	15q23	AC027104	692224			NR_003136
HGNC:41489	FBXO36-IT1	2q36.3			ENSG00000231534		
HGNC:43894	FENDRR	16q24.1	AK091834	400550	ENSG00000268388	24381249, 23369715,	NR_033925
HGNC:26652	FER1L6-AS1	8q24.13		439941	ENSG00000181171		NR_040044
HGNC:26534	FER1L6-AS2	8q24.13	AK057332	157376	ENSG00000253868		
HGNC:41001	FEZF1-AS1	7q31.32	AK057037	154860	ENSG00000230316		NR_036484
HGNC:40410	FGD5-AS1	3p25.1	BC033386	100505641	ENSG00000225733		NR_046251
HGNC:49382	FGF10-AS1	5p12	BC042062	101927075	ENSG00000248464	24147006	
HGNC:40234	FGF12-AS1	3q28			ENSG00000231383		
HGNC:40235	FGF12-AS2	3q28			ENSG00000230126		
HGNC:40236	FGF12-AS3	3q28			ENSG00000226709		
HGNC:44264	FGF13-AS1	Xq26.3	BC023610	100129662	ENSG00000226031		NR_038405
HGNC:40237	FGF14-AS1	13q33.1	AI217925	100874081	ENSG00000234445		
HGNC:44368	FGF14-AS2	13q33.1	BC033993, BM674599,	283481			NR_036487
HGNC:42774	FGF14-IT1	13q33.1		283480	ENSG00000243319		
HGNC:49627	FIRRE	Xq26.2	AK097911	286467	ENSG00000213468	24463464	NR_026975
HGNC:27913	FLG-AS1	1q21.3	BC041408, AK056431	339400	ENSG00000237975	12477932	
HGNC:40239	FLNB-AS1	3p14.3		100506906	ENSG00000244161		
HGNC:39077	FLVCR1-AS1	1q32.3		642946	ENSG00000198468	11943475	NR_027285
HGNC:39081	FMR1-AS1	Xq27.3		100126270		17921506, 18213394	NR_024499
HGNC:41333	FMR1-IT1	Xq27.3			ENSG00000236337		
HGNC:41460	FNDC1-IT1	6q25.3			ENSG00000235086		
HGNC:49789	FOCAD-AS1	9p21.3	AI288976, AW363020	101929548	ENSG00000227071		
HGNC:50665	FOXC2-AS1	16q24.1	AW271272	103752587	ENSG00000260944	26408180	NR_125795
HGNC:50650	FOXCUT	6p25.3	AW002296			24889262, 25031703	
HGNC:50658	FOXD1-AS1	5q13.2	AA769738		ENSG00000247993		

HGNC:44256	FOXD2-AS1	1p33	BC006113	84793	ENSG00000237424		NR_026878
HGNC:40241	FOXD3-AS1	1p31.3	KJ493778	100996301	ENSG00000230798		
HGNC:50663	FOXG1-AS1	14q12	DA239559	103695363	ENSG00000257126	24007600	NR_125758
HGNC:44290	FOXN3-AS1	14q32.11	BC067103	400236	ENSG00000258920		NR_036500
HGNC:30119	FOXN3-AS2	14q32.11		29018	ENSG00000259073		NR_024620
HGNC:41942	FOXP1-AS1	3p13			ENSG00000244203		
HGNC:41335	FOXP1-IT1	3p13		100506815	ENSG00000242094		
HGNC:50332	FOXP4-AS1	6p21.1			ENSG00000234753		
HGNC:39964	FREM2-AS1	13q13.3			ENSG00000225350		
HGNC:51844	FRGCA	21q22.3			ENSG00000236663	26261500	
HGNC:20129	FRMD6-AS1	14q21.3		145438			NR_037676
HGNC:43637	FRMD6-AS2	14q22.1			ENSG00000258537		
HGNC:41239	FRMPD3-AS1	Xq22.3			ENSG00000227610		
HGNC:41222	FRMPD4-AS1	Xp22.2			ENSG00000223487		
HGNC:39725	FRY-AS1	13q13.1	BC035084	100507099	ENSG00000237637		
HGNC:40978	FSIP2-AS1	2q32.1		102724349	ENSG00000231646		
HGNC:40243	FTCD-AS1	21q22.3	AA905381, AI217733		ENSG00000237338		
HGNC:43758	FTO-IT1	16q12.2	CA450585	100505692			
HGNC:37190	FTX	Xq13.2	AK057701	100302692	ENSG00000230590	12045143, 21254562,	NR_028379
HGNC:44294	FUT8-AS1	14q23.3	AK094893, BX098734,	645431			NR_024334
HGNC:48632	FZD10-AS1	12q24.33	AK123838	440119	ENSG00000250208		
HGNC:44157	GABPB1-AS1	15q21.2		100129387	ENSG00000244879	24036268	NR_024490
HGNC:40249	GABRG3-AS1	15q12	DB073112, BU567553	101928869	ENSG00000228740		NR_120343
HGNC:48336	GACAT1	2q12.3	DB096621		ENSG00000232991	23645148, 24833871	
HGNC:50516	GACAT2	18p11.22	BM709340	100287082	ENSG00000265962	24833871, 24063685, 24961350, 250[illegible]	
HGNC:50847	GACAT3	2p24.3	BX375330, BX107472		ENSG00000236289	24969565, 25077925	
HGNC:51308	GAPLINC	18p11.31	BM782281, AJ707365	100505592	ENSG00000266835	25277524	NR_110429
HGNC:52261	GAS1RR	9q21.33		100506834	ENSG00000226237	26418365	NR_049794
HGNC:16355	GAS5	1q25.1	BC038733	60674	ENSG00000234741	9819378, 23726844	NR_002578
HGNC:44119	GAS5-AS1	1q25.1	AF088026	100506046	ENSG00000270084		NR_037605
HGNC:39826	GAS6-AS1	13q34		650669	ENSG00000233695	23979857	
HGNC:43694	GAS6-AS2	13q34	AK126042	100506394	ENSG00000272695		NR_024609
HGNC:1197	GAS8-AS1	16q24.3	AF050080	750	ENSG00000221819	9790751	NR_122031
HGNC:51108	GATA2-AS1	3q21.3		101927167	ENSG00000244300		
HGNC:33786	GATA3-AS1	10p14	BC036297, AK128810	399717	ENSG00000197308	23870669	NR_104327
HGNC:48840	GATA6-AS1	18q11.2	BM742401	100128893	ENSG00000266010	23846333, 23382218	
HGNC:28126	GCC2-AS1	2q12.3	BC004487	644903	ENSG00000214184		
HGNC:41244	GCSAML-AS1	1q44		148824	ENSG00000227135		NR_027309
HGNC:43592	GDNF-AS1	5p13.2	DA359107		ENSG00000248587	22081608	
HGNC:40956	GFOD1-AS1	6p23			ENSG00000237786		
HGNC:49425	GHET1	7q36.1	AK123072, BC037977	102723099		24397586	
HGNC:33885	GHRLOS	3p25.3	EF549568	100126793	ENSG00000240288	17727735, 18954468	NR_004431
HGNC:40255	GK-AS1	Xp21.2			ENSG00000243055		
HGNC:41339	GK-IT1	Xp21.2			ENSG00000229331		
HGNC:48823	GLIDR	9q21.11	AK092305	389741		23726844	NR_015363
HGNC:51381	GLIS2-AS1	16p13.3	BX104227, AI694962	101926896	ENSG00000262686		NR_110901
HGNC:28260	GLIS3-AS1	9p24.2	BC007366	84850	ENSG00000237009	12477932	NM_032764
HGNC:51588	GLTSCR1-AS1	19q13.33	DB210983		ENSG00000269806		
HGNC:51587	GLTSCR2-AS1	19q13.33	DB337897		ENSG00000269656		
HGNC:41043	GLYCTK-AS1	3p21.1			ENSG00000242797		
HGNC:48993	GMDS-AS1	6p25.3		100508120	ENSG00000250903		
HGNC:50451	GNA14-AS1	9q21.2	BI830360	101927422	ENSG00000231373		
HGNC:24872	GNAS-AS1	20q13.32	AJ251759	149775	ENSG00000235590	10749992	NR_002785
HGNC:43938	GNG12-AS1	1p31.3		100289178	ENSG00000232284	23871723	

HGNC:39886	GPC5-AS1	13q31.3			ENSG00000235984		
HGNC:39887	GPC5-AS2	13q31.3			ENSG00000232885		
HGNC:41340	GPC5-IT1	13q31.3			ENSG00000236240		
HGNC:39909	GPC6-AS1	13q31.3			ENSG00000236520		
HGNC:39910	GPC6-AS2	13q31.3			ENSG00000224394		
HGNC:48602	GPR1-AS	2q33.3	AB774455	101669764		23764515	
HGNC:40259	GPR50-AS1	Xq28		100128688	ENSG00000234696		
HGNC:44163	GPR158-AS1	10p12.1	AK094758	100128811	ENSG00000233642		NR_027333
HGNC:44131	GRID1-AS1	10q23.1	AK097655	100507470			NR_038986
HGNC:16458	GRIK1-AS1	21q22.11	AF107258	642976	ENSG00000174680		NR_027021
HGNC:1282	GRIK1-AS2	21q22.11	AY081145	100379661			NR_033368
HGNC:49478	GRK5-IT1	10q26.11		101927868	ENSG00000228485		XR_246196
HGNC:40265	GRM5-AS1	11q14.2			ENSG00000255082		
HGNC:40267	GRM7-AS1	3p26.1			ENSG00000236202		
HGNC:40266	GRM7-AS2	3p26.1			ENSG00000237665		
HGNC:42444	GRM7-AS3	3p26.1		101927347	ENSG00000226258		
HGNC:48999	GRPEL2-AS1	5q32	AI857303		ENSG00000253618		
HGNC:39917	GRTP1-AS1	13q34			ENSG00000225083		
HGNC:23372	GSN-AS1	9q34.11	AF220263	57000	ENSG00000235865		
HGNC:40269	GTF3C2-AS1	2p23.3		100505624	ENSG00000234945		
HGNC:18406	GTSCR1	18q22.2	AY262164	220158	ENSG00000263417	12682296	
HGNC:27163	GTSE1-AS1	22q13.31	BC015721	150384			
HGNC:40270	GYG2-AS1	Xp22.33			ENSG00000235483		
HGNC:27953	H1FX-AS1	3q21.3	AK091470	339942	ENSG00000206417	14702039	NR_026991
HGNC:4713	H19	11p15.5	AF087017	283120	ENSG00000130600	2595451, 1688465	NR_002196
HGNC:43755	HAGLR	2q31.1	BC030713	401022	ENSG00000224189	24381249, 24555823	NR_033979
HGNC:50646	HAGLROS	2q31.1	BE798911, AA780126	102800310	ENSG00000226363		
HGNC:48872	HAND2-AS1	4q34.1		79804	ENSG00000237125	18171985, 19348682	
HGNC:41342	HAO2-IT1	1p12			ENSG00000230921		
HGNC:33117	HAR1A	20q13.33	DQ860409, BC035016,	768096	ENSG00000225978	16915236	NR_003244
HGNC:33118	HAR1B	20q13.33	DQ860410, DQ860411	768097	ENSG00000231133	16915236	NR_003245
HGNC:34340	HAS2-AS1	8q24.13	AY941178	594842	ENSG00000248690		NR_002835
HGNC:48612	HCCAT5	16q22.3		283902	ENSG00000260880	20130911, 23314567	NR_027756
HGNC:40273	HCFC1-AS1	Xq28			ENSG00000235802		
HGNC:21241	HCG4	6p22.1	AF036977	54435	ENSG00000176998		NR_002139
HGNC:22919	HCG4B	6p22.1	X81005	80868	ENSG00000227262	10557312	NR_001317
HGNC:21243	HCG9	6p21.3	AB088085	10255	ENSG00000204625	10727083, 10557312	NR_028032
HGNC:17707	HCG11	6p22.2	AK024111	493812	ENSG00000228223		
HGNC:18323	HCG14	6p21			ENSG00000224157		
HGNC:18361	HCG15	6p21			ENSG00000227214		
HGNC:20424	HCG16	6p21.3			ENSG00000244349		
HGNC:31339	HCG17	6p21	AB023055	414778	ENSG00000270604		NR_052012
HGNC:31337	HCG18	6p21.3	AK056160	414777	ENSG00000231074		NR_024052
HGNC:31334	HCG20	6p21			ENSG00000228022		
HGNC:31335	HCG21	6p21			ENSG00000233529		
HGNC:19713	HCG23	6p21			ENSG00000228962		
HGNC:23500	HCG24	6p21			ENSG00000230313		
HGNC:20196	HCG25	6p21			ENSG00000232940		
HGNC:29671	HCG26	6p21.3	AB088105	352961			NR_002812
HGNC:27366	HCG27	6p21.3	AK097442	253018	ENSG00000206344	12477932	NR_026791
HGNC:21659	HCP5	6p21.3	D88650	10866	ENSG00000206337	8462994, 10199916	NR_040662
HGNC:30984	HCP5B	6p21.3	BC035828	352990			
HGNC:40868	HDAC11-AS1	3p25.1			ENSG00000244502		
HGNC:48679	HECTD2-AS1	10q23.32		100188947			NR_024467

HGNC:41465	HECW1-IT1	7p14.1		100127950	ENSG00000181211		
HGNC:45049	HEIH	5q35.3	BC019622	100859930		21769904, 23359273	NR_045680
HGNC:43984	HELLPAR	12q23.2	JX088243	101101692	ENSG00000281344	23093777	
HGNC:25810	HEXA-AS1	15q23		80072	ENSG00000260339	12477932	NR_027262
HGNC:43650	HEXDC-IT1	17q25.3	AK129961	100505970			
HGNC:42439	HHATL-AS1	3p22.1			ENSG00000230970		
HGNC:44182	HHIP-AS1	4q31.21	BC039450	646576	ENSG00000248890		NR_037595
HGNC:51181	HID1-AS1	17q25.1		102723641	ENSG00000263586		NR_110878
HGNC:43014	HIF1A-AS1	14q23.2	CN270307	100750246		21897117, 24875884	
HGNC:43015	HIF1A-AS2	14q23.2		100750247		21897117	NR_045406
HGNC:50576	HIPK1-AS1	1p13.2		101928846	ENSG00000235527		
HGNC:39762	HLA-DQB1-AS1	6p21.32			ENSG00000223534		
HGNC:26645	HLA-F-AS1	6p22.1	AK092748	285830	ENSG00000214922		NR_026972
HGNC:41343	HLCS-IT1	21q22.13			ENSG00000237646		
HGNC:40554	HLTF-AS1	3q24			ENSG00000239718		
HGNC:42509	HLX-AS1	1q41	DA373928	100873924	ENSG00000257551		NR_046901
HGNC:41940	HM13-AS1	20q11.21			ENSG00000230613		
HGNC:41424	HM13-IT1	20q11.21			ENSG00000235313		
HGNC:43632	HMBOX1-IT1	8p12			ENSG00000259196		
HGNC:48984	HMGN3-AS1	6q14.1		100288198	ENSG00000270362		
HGNC:49149	HMMR-AS1	5q34	BU852496	101927813	ENSG00000251018		
HGNC:26785	HNF1A-AS1	12q24.31	AK096009	283460	ENSG00000241388	24000294, 25863539	NR_024345
HGNC:49505	HNF4A-AS1	20q13.12	AF143870	101927219	ENSG00000229005	24344320	
HGNC:50729	HORMAD2-AS1	22q12.2		101929664	ENSG00000227117	25144376	
HGNC:33510	HOTAIR	12q13.13	DQ926657	100124700	ENSG00000228630	17604720, 17604716	NR_003716
HGNC:37117	HOTAIRM1	7p15.2		100506311	ENSG00000233429	19144990	
HGNC:37461	HOTTIP	7p15.2	GU724873	100316868	ENSG00000243766	21483457, 21423168,	NR_037843
HGNC:43745	HOXA-AS2	7p15.2	BC035889	285943	ENSG00000253552	23649634	NR_122069
HGNC:43748	HOXA-AS3	7p15.2	AK091933	100133311	ENSG00000254369		NR_038831
HGNC:40281	HOXA10-AS	7p15.2	AW195063, BM549446	100874323	ENSG00000253187		
HGNC:24957	HOXA11-AS	7p15.2		221883	ENSG00000240990	9745033, 12050232	NR_002795
HGNC:43744	HOXB-AS1	17q21.32	BF875734, BE676309, AI292043		ENSG00000230148		
HGNC:40284	HOXB-AS2	17q21.32	DA447098, BF999536		ENSG00000239552		
HGNC:40283	HOXB-AS3	17q21.32	BC010732	404266	ENSG00000233101		
HGNC:40285	HOXB-AS4	17q21.32	DB109357, AI940041		ENSG00000242207		
HGNC:43749	HOXC-AS1	12q13.13	BU619815, CA429707	100874363	ENSG00000250451		
HGNC:43750	HOXC-AS2	12q13.13	AI935658, BF195843		ENSG00000250133		
HGNC:43751	HOXC-AS3	12q13.13	BC035260		ENSG00000251151		
HGNC:43753	HOXC13-AS	12q13.13	CN364530	100874366	ENSG00000249641		
HGNC:43756	HOXD-AS2	2q31.1	BC034000	100506783	ENSG00000237380		NR_038435
HGNC:47041	HPN-AS1	19q13.12		100128675	ENSG00000227392		
HGNC:5169	HPVC1	7p11.2	X85042	3262		7581372	NR_004422
HGNC:16071	HPYR1	8q24.22	AF200341	93668	ENSG00000253521		NR_026684
HGNC:41474	HS1BP3-IT1	2p24.1			ENSG00000231948		
HGNC:40870	HS6ST2-AS1	Xq26.2			ENSG00000235849		
HGNC:40289	HTR2A-AS1	13q14.2			ENSG00000224517		
HGNC:41032	HTR3E-AS1	3q27.1			ENSG00000238020		
HGNC:48956	HTR5A-AS1	7q36.2	BC031272, BC045795	100128264	ENSG00000220575		NR_038945
HGNC:37118	HTT-AS	4p16.3		100750326	ENSG00000251075	21672921, 23756188	
HGNC:34232	HULC	6p24.3		728655	ENSG00000251164	17241883, 23728852	NR_004855
HGNC:40631	HUNK-AS1	21q22.11	CD689785		ENSG00000237138		
HGNC:41173	HYI-AS1	1p34.2			ENSG00000229348		
HGNC:5326	HYMAI	6q24.2	AF241534	57061		10936046	NR_002768
HGNC:32062	IBA57-AS1	1q42.13	AK057440	574432	ENSG00000203684		

HGNC:51103	ID2-AS1	2p25.1		100506299	ENSG00000235092		
HGNC:40292	IDH1-AS1	2q34	AI492612, BE465811	100507475	ENSG00000231908		
HGNC:30885	IDI2-AS1	10p15.3	AF220183	55853	ENSG00000232656	24036268	NR_024628
HGNC:43910	IFNG-AS1	12q15	AK124066, BF510189	100885789	ENSG00000255733	11735227, 24056746	NR_104124
HGNC:49806	IFT74-AS1	9p21.2	AA960898, AI214381	101929602	ENSG00000234676		
HGNC:40295	IGBP1-AS1	Xq13.1			ENSG00000203588		
HGNC:40294	IGBP1-AS2	Xq13.1			ENSG00000220925		
HGNC:14062	IGF2-AS	11p15.5	AB030733	51214	ENSG00000099869	10731720	NM_016412
HGNC:32674	IGF2BP2-AS1	3q27.2	AK057462	646600	ENSG00000163915		NR_027317
HGNC:40296	IGFBP7-AS1	4q12		255130	ENSG00000245067		
HGNC:40777	IGSF11-AS1	3q13.32		100506765	ENSG00000239877		
HGNC:44303	IL10RB-AS1	21q22.11		100288432	ENSG00000223799		NR_038974
HGNC:49094	IL12A-AS1	3q25.33	AK097161	101928376	ENSG00000244040		
HGNC:40298	IL20RB-AS1	3q22.3			ENSG00000249407		
HGNC:40299	IL21-AS1	4q27	BC045668		ENSG00000227145		
HGNC:27551	IL21R-AS1	16p12.1	AK093371	283888	ENSG00000259954		NR_037158
HGNC:27115	ILF3-AS1	19p13.2	BC007593, BC044911,	147727		12477932	NR_024333
HGNC:6060	INE1	Xp11.4-p11.3	Y10696	8552	ENSG00000224975	9244435	NR_024616
HGNC:6061	INE2	Xp22.2	Y10697	8551		9244435	NR_002725
HGNC:40303	INHBA-AS1	7p14.1		285954	ENSG00000224116		NR_027118
HGNC:42691	INTS6-AS1	13q14.3	AA397528	100507398	ENSG00000236778		
HGNC:41180	INTS6L-AS1	Xq26.3			ENSG00000225235		
HGNC:40892	IPO9-AS1	1q32.1	BI916758		ENSG00000231871		
HGNC:6109	IPW	15q11.2	U12897	3653		7849716, 9601023	NR_023915
HGNC:41137	IQCA1-AS1	2q37	BF509914		ENSG00000232893		
HGNC:41297	IQCF5-AS1	3p21.2		101928999	ENSG00000235455		
HGNC:44104	IQCH-AS1	15q23		100506686	ENSG00000259673		NR_040051
HGNC:41303	IQCJ-SCHIP1-AS	3q25.33			ENSG00000241211		
HGNC:50365	IRAIN	15q26.3				25092925	
HGNC:44313	ISM1-AS1	20p12.1	BC038745, BX092222,	100505536	ENSG00000226263		NR_040043
HGNC:48962	ISPD-AS1	7p21.2		100506025	ENSG00000229688		
HGNC:41188	ISX-AS1	22q12.3		101926957	ENSG00000227895		
HGNC:40659	ITCH-AS1	20q11.22			ENSG00000236388		
HGNC:41397	ITCH-IT1	20q11.22			ENSG00000231795		
HGNC:51383	ITFG1-AS1	16q12.1	BE551587, BI462694, A	101927102	ENSG00000260281		NR_110903
HGNC:49668	ITGA9-AS1	3p22.2	BC040563	101928153	ENSG00000235257		
HGNC:44304	ITGB2-AS1	21q22.3	BC040064, BC051807	100505746	ENSG00000227039		NR_038311
HGNC:40309	ITGB5-AS1	3q21.2			ENSG00000244286		
HGNC:40310	ITIH4-AS1	3p21.1			ENSG00000239799		
HGNC:20132	ITPK1-AS1	14q32.12	AK024887	319085	ENSG00000258730		NR_002808
HGNC:40312	ITPKB-AS1	1q42.12			ENSG00000228548		
HGNC:41349	ITPKB-IT1	1q42.12		100506443	ENSG00000228382		
HGNC:44470	ITPR1-AS1	3p26.1	BU629368	100996539	ENSG00000231249		
HGNC:48601	JADRR	4q28.2	KC469579	101669763		24097061	
HGNC:27203	JAKMIP2-AS1	5q32	AK027578	153469			NR_038902
HGNC:40314	JARID2-AS1	6p22.3	AW340306, AW665239	100506681	ENSG00000235488		
HGNC:41218	JAZF1-AS1	7p15.1		100128081	ENSG00000234336		NR_034097
HGNC:48959	JHDM1D-AS1	7q34		100134229	ENSG00000260231		
HGNC:28222	JMJD1C-AS1	10q21.3	AK314171	84989		12477932	NR_027182
HGNC:37191	JPX	Xq13.2	AV714079	554203	ENSG00000225470	21029862, 21254562	NR_024582
HGNC:43670	JRKL-AS1	11q21	AL706355, BE972282		ENSG00000255679		
HGNC:43740	KANSL1-AS1	17q21.31		644246	ENSG00000214401		NR_034172
HGNC:49510	KANTR	Xp11.22	EF565109, CF994433	102723508	ENSG00000232593	24381249	
HGNC:49147	KBTBD11-OT1	8p23.3	BX104191		ENSG00000253696		

HGNC:40316	KCNAB1-AS1	3q25.31			ENSG00000242370		
HGNC:40315	KCNAB1-AS2	3q25.31			ENSG00000240596		
HGNC:39958	KCNC4-AS1	1p13.3			ENSG00000224965		
HGNC:40317	KCND3-AS1	1p13.2			ENSG00000237556		
HGNC:41350	KCND3-IT1	1p13.2			ENSG00000232558		
HGNC:41351	KCNH1-IT1	1q32.2			ENSG00000234233		
HGNC:48680	KCNIP2-AS1	10q24.32		100289509	ENSG00000226009		
HGNC:29895	KCNIP4-IT1	4p15.31	BK000166	359822		12515386	NR_002813
HGNC:43720	KCNJ2-AS1	17q24.3	AK093963	400617			NR_036534
HGNC:41352	KCNJ6-AS1	21q22.13			ENSG00000233213		
HGNC:49901	KCNK15-AS1	20q13.12	BF195580		ENSG00000244558	25430712	
HGNC:51213	KCNMA1-AS1	10q22.3	BX092192	101929328	ENSG00000236467		NR_120655
HGNC:51214	KCNMA1-AS2	10q22.3		101929310	ENSG00000225497		NR_120654
HGNC:51215	KCNMA1-AS3	10q22.3	AA453582, AW510993	101929286	ENSG00000225652		
HGNC:51409	KCNMB2-AS1	3q26.32	BG977814, BG206373, DA735220		ENSG00000237978	25394782	
HGNC:42790	KCNQ1-AS1	11p15.4	AJ251642	338653	ENSG00000229414		
HGNC:13335	KCNQ1DN	11p15.5	AB039920	55539	ENSG00000237941	11056398, 11063728,	NR_024627
HGNC:6295	KCNQ1OT1	11p15.5		10984	ENSG00000269821	10220444, 12772698	NR_002728
HGNC:40323	KCNQ5-AS1	6q13			ENSG00000229154		
HGNC:41354	KCNQ5-IT1	6q13		100507381	ENSG00000233844		
HGNC:48674	KCTD21-AS1	11q14.1		100289388	ENSG00000246174		
HGNC:40528	KDM4A-AS1	1p34.1	BC073819	100132774	ENSG00000236200		NR_033827
HGNC:41384	KDM5C-IT1	Xp11.22			ENSG00000235262		
HGNC:43440	KIAA0196-AS1	8q24.13			ENSG00000253167		
HGNC:50644	KIAA1614-AS1	1q25.3	AK026359	103344928	ENSG00000232586	24886442	
HGNC:26822	KIF9-AS1	3p21.31		285352	ENSG00000227398		
HGNC:20953	KIF25-AS1	6q27	AB016899	100505879	ENSG00000229921	10382971	
HGNC:41412	KIRREL-IT1	1q23.1		100505785	ENSG00000226520		
HGNC:42655	KIRREL3-AS1	11q24.2			ENSG00000257271		
HGNC:42656	KIRREL3-AS2	11q24.2			ENSG00000254960		
HGNC:26855	KIRREL3-AS3	11q24.2	AK097543	283165	ENSG00000218109		
HGNC:51231	KIZ-AS1	20p11.23	BC034426	101929591	ENSG00000232712		NR_109956
HGNC:25796	KLF3-AS1	4p14		79667	ENSG00000231160		
HGNC:41355	KLF7-IT1	2q33.3			ENSG00000237892		
HGNC:40850	KLHL6-AS1	3q27.1			ENSG00000242522		
HGNC:43431	KLHL7-AS1	7p15.3	AK054880	100775104	ENSG00000230658		NR_046220
HGNC:31018	KLHL30-AS1	2q37.3	BC031945	394261			
HGNC:40845	KMT2E-AS1	7q22.3		100216545	ENSG00000239569		
HGNC:42942	KRBOX1-AS1	3p22.1	AK057338	100506275	ENSG00000206552		XR_110049
HGNC:49607	KRT73-AS1	12q13.13	AK128146	100127967	ENSG00000257495		
HGNC:27877	KRTAP5-AS1	11p15.5		338651	ENSG00000233930		
HGNC:19842	KTN1-AS1	14q22.3		100129075	ENSG00000186615		NR_027123
HGNC:51320	L3MBTL4-AS1	18p11.31	BC040631	101927150	ENSG00000264707		NR_110765
HGNC:27841	LACTB2-AS1	8q13.3		286190	ENSG00000246366		NR_038881
HGNC:40334	LAMA5-AS1	20q13.33		101928158	ENSG00000228812		
HGNC:40754	LAMP5-AS1	20p12.2		101929329	ENSG00000225988	24488769	
HGNC:40823	LAMTOR5-AS1	1p13.3	HY022359, BE547292, BG718030		ENSG00000224699		
HGNC:50727	LANCL1-AS1	2q34	AA447268	102724820	ENSG00000234281		
HGNC:40336	LARGE-AS1	22q12.3		100506195	ENSG00000224973		
HGNC:41357	LARGE-IT1	22q12.3			ENSG00000232081		
HGNC:40796	LARS2-AS1	3p21.31			ENSG00000232455		
HGNC:39912	LATS2-AS1	13q12.11			ENSG00000233851		
HGNC:48678	LBX1-AS1	10q24.32	AK096698, AK123344,	399806	ENSG00000227128		NR_029380
HGNC:25136	LBX2-AS1	2p13.1	AK125271, AK096725	151534	ENSG00000257702	24905231	NR_024606

HGNC:25837	LCAL1	6q14.1		80078		25116943, 25864709	NR_130915
HGNC:51177	LCMT1-AS1	16p12.1		102723510	ENSG00000260448		NR_120306
HGNC:51178	LCMT1-AS2	16p12.1		100506655	ENSG00000260034		NR_039998
HGNC:48592	LDLRAD4-AS1	18p11.21	BC042085	100288122	ENSG00000267690		NR_040031
HGNC:40339	LEF1-AS1	4q25		641518	ENSG00000232021		NR_029373
HGNC:44132	LEMD1-AS1	1q32.1	AK090497	284576	ENSG00000226235		NR_038425
HGNC:40705	LENG8-AS1	19q13.42			ENSG00000226696		
HGNC:40340	LGALS8-AS1	1q43		100287902	ENSG00000223776		
HGNC:40341	LHFPL3-AS1	7q22.2	BC012751	645591	ENSG00000226869		NR_034142
HGNC:44106	LHFPL3-AS2	7q22.3	BC050337	723809			NR_027374
HGNC:40982	LHX4-AS1	1q25.2		100527964			
HGNC:49570	LHX5-AS1	12q24.13	DB553737		ENSG00000257935	23382218	
HGNC:43600	LIFR-AS1	5p13.1	BC038371	100506495	ENSG00000244968		
HGNC:44107	LIMD1-AS1	3p21.31	AK026206	644714	ENSG00000230530		NR_033947
HGNC:40342	LIMS1-AS1	2q12.3	AA861200		ENSG00000228763		
HGNC:21553	LIN28B-AS1	6q21	AW612153, BF223582	100113403	ENSG00000203809		
HGNC:26885	LINC-PINT	7q32.3	BC130416	378805	ENSG00000231721	24070194, 24381249	NR_015431
HGNC:43773	LINC-ROR	18q21.31	HQ315778	100885779	ENSG00000258609	21057500, 23208419,	NR_048536
HGNC:16224	LINC00028	20q11.21	AL121751	140875	ENSG00000233354		NR_024358
HGNC:16184	LINC00029	20q13.33	AL117533, BC069708,	100144596	ENSG00000125514	11780052	NR_028295
HGNC:16506	LINC00032	9p21	AF418573	158035	ENSG00000231459		NR_026679
HGNC:32028	LINC00051	8q24.3	BC008253	619434	ENSG00000254008		
HGNC:26455	LINC00052	15q25.3	AK056023	145978			XR_017978
HGNC:34521	LINC00083	1q25.2	AK125993	400797	ENSG00000188585		XR_040317
HGNC:31408	LINC00092	9q22.32	BC043559	100188953	ENSG00000225194		
HGNC:24742	LINC00094	9q34	AK092667	266655	ENSG00000235106	12477932	
HGNC:30470	LINC00102	Xp22.33 and Yp11.31			ENSG00000230542		
HGNC:31843	LINC00106	Xp22.33 and Yp11.32			ENSG00000236871		
HGNC:1262	LINC00111	21q22.3	BX099637	54090	ENSG00000227702		
HGNC:1263	LINC00112	21q22.3		54089	ENSG00000232401		NR_024028
HGNC:1264	LINC00113	21q21.3		54088	ENSG00000225298		
HGNC:1265	LINC00114	21q22.2		400866	ENSG00000223806		XR_041645
HGNC:26211	LINC00115	1p36.33	BC017762	79854	ENSG00000225880	12477932	XR_017693
HGNC:28717	LINC00152	2p11.2	BC009508	112597	ENSG00000222041	24523021, 24036268,	XR_042051
HGNC:1283	LINC00158	21q21.3		54072	ENSG00000185433		
HGNC:1285	LINC00159	21q22.11			ENSG00000230323		
HGNC:1294	LINC00160	21q22.12		54064	ENSG00000230978	26426411	
HGNC:17138	LINC00161	21q21.3	AY063458	118421	ENSG00000226935	12036297	
HGNC:33165	LINC00163	21q22.3	BC036902	727699	ENSG00000234880	16826516	
HGNC:33166	LINC00165	21q22.3	BC009635	727701		16826516	
HGNC:30468	LINC00167	11q24.3	AK055956	440072	ENSG00000233220		XR_040639
HGNC:33791	LINC00173	12q24.22	AC090670, BC038547,	100287569	ENSG00000196668		NR_027345
HGNC:27788	LINC00174	7q11.21	AK091213	285908	ENSG00000179406		NR_026873
HGNC:27655	LINC00176	20q13.33		284739	ENSG00000196421		NR_027686
HGNC:37192	LINC00184	1q42.3	AJ421679	100302691	ENSG00000224939		
HGNC:18461	LINC00189	21q22.11	AF490769	193629	ENSG00000215533	12036298	NR_027072
HGNC:30974	LINC00200	10p15.3	AK097673	399706	ENSG00000229205		NR_015376
HGNC:24672	LINC00202-1	10p12.1	AK097405	387644	ENSG00000232224		NR_026795
HGNC:44917	LINC00202-2	10p12.1		731789	ENSG00000231976		NR_026794
HGNC:16420	LINC00205	21q22.3	AF426264	102723489	ENSG00000223768	12036297	
HGNC:37255	LINC00207	22q13.31	BC144508	388910	ENSG00000187012		NR_028409
HGNC:15535	LINC00208	8p23.1	AJ291678		ENSG00000170983		
HGNC:37458	LINC00210	1q41			ENSG00000231814		
HGNC:37459	LINC00211	2p22.2		101929559	ENSG00000237803		

HGNC:20191	LINC00216	14q22.3	AF116641	55451			
HGNC:20169	LINC00221	14q32.33	AK058096	338005	ENSG00000270816		NR_027457
HGNC:21560	LINC00222	6q21	AY927445	387111	ENSG00000203801		
HGNC:20168	LINC00226	14q32.33	AW205017	338004	ENSG00000276210		
HGNC:13240	LINC00229	22q13.3			ENSG00000234300		
HGNC:14138	LINC00235	16p13.3	AW298011	64493			NR_024121
HGNC:38166	LINC00237	20p11.23			ENSG00000225127	23034868	
HGNC:20100	LINC00238	14q23.3		440184	ENSG00000196553		NR_024338
HGNC:20119	LINC00239	14q32.31		145200			NR_026774
HGNC:18772	LINC00240	6p21		100133205	ENSG00000224843		NR_026775
HGNC:21249	LINC00242	6q28	AK056013	401288	ENSG00000229214		NR_026781
HGNC:30956	LINC00243	6p21.33	AK098012	401247	ENSG00000214894		
HGNC:13245	LINC00244	7q36	AF107456	64433		10329000	NR_024119
HGNC:31950	LINC00251	8q13.1	BX110670	552859			NR_038901
HGNC:14160	LINC00254	16p13.3	AI209093	64735			
HGNC:28770	LINC00260	1q32.1	BC000988	84719		12477932	
HGNC:16189	LINC00261	20p11.21		140828	ENSG00000259974	23801869, 22681909,	NR_001558
HGNC:17776	LINC00264	10p12.1		645528	ENSG00000233261		XR_040540
HGNC:28019	LINC00265	7p14.1		349114	ENSG00000188185	12477932	NR_026999
HGNC:16202	LINC00266-1	20q13.33	BC118988	140849	ENSG00000149656		
HGNC:21112	LINC00266-3	6p25.3		441123	ENSG00000170590		
HGNC:38543	LINC00268	9p11.2	AK126863	441426			
HGNC:26586	LINC00269	Xq13.1	AK090929	100996279	ENSG00000215162		
HGNC:32526	LINC00271	6q23.3		100131814	ENSG00000231028		NR_026805
HGNC:26898	LINC00272	1q25.3	AF508909	388719	ENSG00000203729	12801632	NM_00101089
HGNC:38595	LINC00273	16p11.2	AY587847	649159	ENSG00000256642		NR_038368
HGNC:38663	LINC00276	2p24.3	BC035112		ENSG00000230448		
HGNC:38712	LINC00278	Yp11.31			ENSG00000231535		
HGNC:38724	LINC00279	Yp11.2			ENSG00000232348		
HGNC:38803	LINC00280	Yp11.2			ENSG00000229643		
HGNC:26737	LINC00282	13q14.3	AK094626	283521			NR_027047
HGNC:38809	LINC00283	13q33.1			ENSG00000231633		
HGNC:26981	LINC00284	13q14.11	AK001938	121838	ENSG00000233725		NR_026955
HGNC:38515	LINC00290	4q34.3	AF088005	728081	ENSG00000248197		NR_033918
HGNC:39078	LINC00293	8q11.1	AK097475	497634	ENSG00000253314		NR_027012
HGNC:27456	LINC00294	11p13	BC046921	283267			NR_015451
HGNC:39210	LINC00297	13q12.3			ENSG00000224329		
HGNC:49257	LINC00298	2p25.1		339788	ENSG00000235665		
HGNC:27940	LINC00299	2p25.1	AK127578	339789	ENSG00000236790	12477932	NR_034135
HGNC:28603	LINC00301	11q12.2	AK058123	283197	ENSG00000181995	12477932	NR_026946
HGNC:31825	LINC00302	1q21.3	AF005082	388699	ENSG00000176075	9344646	
HGNC:26865	LINC00303	1q32.1	AK097662	284573	ENSG00000176754		NR_027902
HGNC:26713	LINC00304	16q24.3	AK094020	283860	ENSG00000180422		NR_024347
HGNC:28597	LINC00305	18q22.1	BC029565	221241	ENSG00000179676	12477932	NM_152728
HGNC:16727	LINC00307	21q22.11	AY063455	266919	ENSG00000227342	12036297	
HGNC:16023	LINC00308	21q21.1	AY077696	54143	ENSG00000184856	12036298	
HGNC:25279	LINC00309	2p14		150992	ENSG00000230923		
HGNC:16414	LINC00310	21q22.11	AF426258	114036	ENSG00000227456	12036297	NR_027266
HGNC:28312	LINC00311	16q24.1	BC030801	197196	ENSG00000179219	12477932	XR_017879
HGNC:6662	LINC00312	3p25.3	AF086709	29931	ENSG00000237697	12452030, 12918109,	NR_024065
HGNC:16416	LINC00313	21q22.3	AF426261	114038	ENSG00000185186	12036297, 26178480	NR_026863
HGNC:16622	LINC00314	21q21.3	AF427489	246705	ENSG00000178457	12036297	NR_027246
HGNC:16621	LINC00315	21q22.3	AF427488	246704	ENSG00000184274	12036297	
HGNC:19723	LINC00316	21q22.3	BG221750	388830	ENSG00000237664		

HGNC:23126	LINC00317	21q21.1	BC009878	378828	ENSG00000238265		
HGNC:19730	LINC00319	21q22.3	AK095355	284836	ENSG00000188660		NR_026960
HGNC:19690	LINC00320	21q21.1	AK094858	387486	ENSG00000224924		NR_024090
HGNC:33698	LINC00322	21q22.3	AK131425	100126693	ENSG00000237864	17081983	
HGNC:19720	LINC00323	21q22.3	AK094492	284835	ENSG00000226496		
HGNC:26628	LINC00324	17p13.1	AK092109	284029	ENSG00000178977		
HGNC:41926	LINC00326	6q23.2		285735	ENSG00000231023		NR_026969
HGNC:42009	LINC00327	13q12.12		100506697	ENSG00000232977		
HGNC:42028	LINC00328	21p11.2	AF172850	51152			
HGNC:42047	LINC00330	13q14.12	AK056732	144817	ENSG00000235097		
HGNC:42048	LINC00331	13q31.1			ENSG00000225427		
HGNC:42049	LINC00332	13q14.11			ENSG00000230710		
HGNC:42050	LINC00333	13q31.1			ENSG00000233349		
HGNC:16425	LINC00334	21q22.3	AF426268	114042	ENSG00000182586	12036297	
HGNC:33813	LINC00336	6p21.31	AK125740	401253	ENSG00000197251		
HGNC:28620	LINC00337	1p36.31	BC030279	148645	ENSG00000225077	12477932	
HGNC:25011	LINC00339	1p36.12		29092	ENSG00000218510	11042152	
HGNC:20353	LINC00341	14q32.13	AK024929	79686			NR_026779
HGNC:42470	LINC00342	2q11.1		150759	ENSG00000232931		
HGNC:42500	LINC00343	13q33.2		144920	ENSG00000226620		
HGNC:42503	LINC00345	13q14.3			ENSG00000235660		
HGNC:27492	LINC00346	13q34		283487	ENSG00000255874		NR_027701
HGNC:27890	LINC00347	13q22.1		338864	ENSG00000236678	12477932	
HGNC:42658	LINC00348	13q21.33			ENSG00000226846		
HGNC:42667	LINC00349	13q11			ENSG00000231238		
HGNC:42668	LINC00350	13q12.11			ENSG00000225316		
HGNC:42669	LINC00351	13q31.1			ENSG00000226317		
HGNC:42670	LINC00352	13q12.12			ENSG00000227893		
HGNC:42671	LINC00353	13q31.3		100874139	ENSG00000236176		
HGNC:42672	LINC00354	13q34		101928616	ENSG00000226903		
HGNC:27061	LINC00355	13q21.31		144766	ENSG00000227674		
HGNC:42678	LINC00358	13q21.31			ENSG00000229578		
HGNC:42679	LINC00359	13q32.1		100887754	ENSG00000243300		
HGNC:42682	LINC00362	13q12.12			ENSG00000229483		
HGNC:42684	LINC00363	13q31.3	AA885151		ENSG00000232849		
HGNC:42686	LINC00364	13q21.32			ENSG00000230040		
HGNC:42687	LINC00365	13q12.3			ENSG00000224511		
HGNC:42688	LINC00366	13q13.3			ENSG00000229437		
HGNC:42689	LINC00367	13q12.11	BG723634	101930748			
HGNC:42690	LINC00368	13q34	BG720241	101927802	ENSG00000225870		
HGNC:42692	LINC00370	13q33.3	BF695150		ENSG00000223617		
HGNC:42693	LINC00371	13q14.3		647166	ENSG00000226792		
HGNC:42698	LINC00374	13q21.1	BG190276		ENSG00000232954		
HGNC:42699	LINC00375	13q31.1	AW510855		ENSG00000226370		
HGNC:42701	LINC00376	13q21.31	AA759092		ENSG00000227564		
HGNC:42703	LINC00377	13q31.1	BG185523		ENSG00000229246		
HGNC:42704	LINC00378	13q21.2			ENSG00000225249		
HGNC:42705	LINC00379	13q31.3			ENSG00000229557		
HGNC:42706	LINC00380	13q31.3	AA609658		ENSG00000234625		
HGNC:42708	LINC00381	13q22.1			ENSG00000226240		
HGNC:42709	LINC00382	13q31.1	BG717866	101927195	ENSG00000229175		
HGNC:42710	LINC00383	13q21.33	BG527271, BM557184	103689913	ENSG00000237534		NR_125752
HGNC:42711	LINC00384	13q12.3	BX648491		ENSG00000232117		
HGNC:42713	LINC00385	13q12.3	AI203837		ENSG00000232643		

HGNC:42715	LINC00387	13q11			ENSG00000231914		
HGNC:42716	LINC00388	13q11			ENSG00000229788		
HGNC:42718	LINC00390	13q14.11	AW779259		ENSG00000226519		
HGNC:42719	LINC00391	13q32.1			ENSG00000238230		
HGNC:42720	LINC00392	13q22.1			ENSG00000228295		
HGNC:42721	LINC00393	13q22.1			ENSG00000224853		
HGNC:42723	LINC00395	13q21.31			ENSG00000231061		
HGNC:42724	LINC00396	13q34	AA927226		ENSG00000231428		
HGNC:42725	LINC00397	13q31.2	AI286230		ENSG00000223404		
HGNC:42727	LINC00398	13q12.3			ENSG00000237879		
HGNC:42728	LINC00399	13q33.3	BX111569, AI023810		ENSG00000229792		
HGNC:42729	LINC00400	13q14.11			ENSG00000229928		
HGNC:42732	LINC00402	13q22.1		100507612	ENSG00000235532		
HGNC:42733	LINC00403	13q34		100505996	ENSG00000224243		
HGNC:42734	LINC00404	13q34			ENSG00000229520		
HGNC:42739	LINC00407	13q14.12	BG959100		ENSG00000237585		
HGNC:42740	LINC00408	13q11	BG722014	100652856	ENSG00000226250		
HGNC:42742	LINC00410	13q31.3		144776	ENSG00000231674		
HGNC:42744	LINC00411	13q32.3			ENSG00000229599		
HGNC:42745	LINC00412	13q12.2	AI051530		ENSG00000234772		
HGNC:42748	LINC00415	13q12.13			ENSG00000231983		
HGNC:42755	LINC00421	13q12.11		100287114	ENSG00000236834		
HGNC:42758	LINC00423	13q13.1			ENSG00000226968		
HGNC:42815	LINC00424	13q12.11			ENSG00000226722		
HGNC:42761	LINC00426	13q12.3		100188949	ENSG00000238121		
HGNC:42762	LINC00427	13q12.3		100507040	ENSG00000236463		
HGNC:42763	LINC00428	13q14.11	AA206719		ENSG00000229546		
HGNC:42765	LINC00430	13q31.1	AW661815		ENSG00000233528		
HGNC:42766	LINC00431	13q34	BG181389		ENSG00000225760		
HGNC:42768	LINC00433	13q31.2			ENSG00000229443		
HGNC:42769	LINC00434	13q21.2			ENSG00000227336		
HGNC:42772	LINC00437	13q13.3	BM927018		ENSG00000236354		
HGNC:42777	LINC00440	13q31.2			ENSG00000234660		
HGNC:42778	LINC00441	13q14.2		100862704	ENSG00000231473	25579178	NR_046414
HGNC:42779	LINC00442	13q12.11		348021	ENSG00000232685		
HGNC:42780	LINC00443	13q33.3			ENSG00000230156		
HGNC:42781	LINC00444	13q14.2			ENSG00000234689		
HGNC:42782	LINC00445	13q13.3		100507114	ENSG00000236036		
HGNC:42783	LINC00446	13q22.3		100874175	ENSG00000229249		
HGNC:42785	LINC00448	13q21.31			ENSG00000228669		
HGNC:42786	LINC00449	13q32.3			ENSG00000203441		
HGNC:42802	LINC00454	13q34		100874178	ENSG00000226921		
HGNC:42804	LINC00456	13q32.1	BG623605	103625683	ENSG00000233124		
HGNC:42805	LINC00457	13q13.2		100874179	ENSG00000225179		
HGNC:42807	LINC00458	13q14.3	BC026300	100507428	ENSG00000234787		
HGNC:42808	LINC00459	13q21.31			ENSG00000229307		
HGNC:42809	LINC00460	13q33.3		728192	ENSG00000233532		
HGNC:42810	LINC00461	5q14.3		645323	ENSG00000245526	23562822, 25209608,	NR_024384
HGNC:42811	LINC00462	13q14.2			ENSG00000233610		
HGNC:42813	LINC00463	13q12.13	BM666193	101928922	ENSG00000234056		
HGNC:27294	LINC00466	1p31.3		199899	ENSG00000224209	12477932	NR_038252
HGNC:28227	LINC00467	1q32.3	BC005997	84791	ENSG00000153363	24586304	NR_026761
HGNC:26863	LINC00469	17q25.1	AK097638	283982	ENSG00000177338		NM_182564
HGNC:1225	LINC00470	18p11.32	AF295730	56651	ENSG00000132204	11173868	NR_023925

HGNC:28668	LINC00471	2q37.1	BC033054	151477	ENSG00000181798	12477932	NM_173513
HGNC:21380	LINC00472	6q13		79940	ENSG00000233237	25865225	NR_121612
HGNC:21160	LINC00473	6q27	BC008632	90632	ENSG00000223414	22108211	NR_026860
HGNC:23367	LINC00474	9q31.3	AB021923	58483	ENSG00000204148		NM_021208
HGNC:23569	LINC00475	9q22.31	AK023662	158314	ENSG00000225511		
HGNC:27858	LINC00476	9q22.33	AK092137	100128782	ENSG00000175611		
HGNC:26557	LINC00477	12p12.1	AK057456	144360	ENSG00000197503	14702039	NM_144667
HGNC:19727	LINC00479	21q22.3	AK057397	150135	ENSG00000236384		
HGNC:26816	LINC00482	17q25.3	AK096740	284185	ENSG00000185168		NM_178519
HGNC:26080	LINC00483	17q21.33	AK000701	55018	ENSG00000167117		
HGNC:27862	LINC00484	9q22.31	AK095132	100129347	ENSG00000235641		
HGNC:27476	LINC00485	12q23.2	BC037211	283432	ENSG00000258169		NR_033855
HGNC:42946	LINC00486	2p22.3		285045	ENSG00000230876		NR_027099
HGNC:42947	LINC00487	2p25.2		400941	ENSG00000205837		NR_038369
HGNC:32675	LINC00488	3q13.13	AK123226	677779	ENSG00000214381		NR_026767
HGNC:43426	LINC00489	20q11.23	DB341379		ENSG00000225759		
HGNC:43428	LINC00491	5q21.1	CN270888		ENSG00000250682	25200694	
HGNC:43429	LINC00492	5q21.1	CA314239		ENSG00000250958		
HGNC:43430	LINC00493	20p11.23		388789	ENSG00000232388	25392693	NR_015432
HGNC:27657	LINC00494	20q13.13		284749	ENSG00000235621	12477932	
HGNC:43435	LINC00498	4q28.3			ENSG00000248397		
HGNC:43436	LINC00499	4q28.3			ENSG00000251372		
HGNC:43437	LINC00500	4q28.3			ENSG00000249381		
HGNC:43439	LINC00501	3q26.32	AK055528		ENSG00000203645		
HGNC:43442	LINC00502	10q23.31			ENSG00000224851		
HGNC:43555	LINC00504	4p15.33			ENSG00000248360		
HGNC:43556	LINC00505	1p33			ENSG00000225667		
HGNC:43557	LINC00506	3p12.1	BC030609	100846978			
HGNC:43558	LINC00507	12q24.32			ENSG00000256193		
HGNC:43559	LINC00508	12q24.32			ENSG00000256971		
HGNC:43564	LINC00511	17q24.3	AK129994	400619	ENSG00000227036	25864709	
HGNC:27549	LINC00514	16p13.3	AK098017	283875			NR_033861
HGNC:16019	LINC00515	21q21.3	AF086441	282566			
HGNC:19831	LINC00517	14q21.1	AA029430	400208	ENSG00000259091		
HGNC:28626	LINC00518	6p24.3	BC028118	221718	ENSG00000183674	12477932, 24906614	NR_027793
HGNC:19838	LINC00519	14q22.1	BG121773	161342	ENSG00000258955		
HGNC:19843	LINC00520	14q22.3	BF572611	645687	ENSG00000258791		
HGNC:19860	LINC00521	14q32.12	BI463117	256369	ENSG00000175699		
HGNC:20117	LINC00523	14q32.2		283601	ENSG00000196273		NR_024096
HGNC:20118	LINC00524	14q32.31	AA843471	338002	ENSG00000259023		
HGNC:40290	LINC00525	7p12.3	BC007354	84847	ENSG00000146666		
HGNC:28278	LINC00526	18p11.31	BC010538	147525		12477932	NR_026849
HGNC:17215	LINC00527	21q22.2	AJ011409	54748			
HGNC:26875	LINC00528	22q11.21	AK097861	200298		14702039	
HGNC:15544	LINC00529	8p23-p22	AJ301561	83647	ENSG00000236827	11896452	
HGNC:18690	LINC00533	6p21	AA913908, AI688709	387055	ENSG00000235570		
HGNC:43643	LINC00534	8q21.3			ENSG00000253394		
HGNC:43644	LINC00535	8q22.1		642924	ENSG00000246662		NR_033858
HGNC:43645	LINC00536	8q23.3	BC040336		ENSG00000249917		
HGNC:43654	LINC00537	9q13	BC110369	203274			
HGNC:43655	LINC00538	1q32.3	JF495099	100861504		22258142	NR_046189
HGNC:43672	LINC00539	13q12.11	BG940872	100652865	ENSG00000224429		
HGNC:43673	LINC00540	13q12.11	AK054845, BC035104	100506622			
HGNC:43678	LINC00543	13q12.2	CB142335, AI262987	100132234			

HGNC:43679	LINC00544	13q12.3		440131	ENSG00000122043		NR_033889
HGNC:43680	LINC00545	13q12.3	AW971998, AA514237	440132	ENSG00000236094		
HGNC:43682	LINC00547	13q13.3	AK092066	400121			NR_040244
HGNC:43683	LINC00548	13q14.11	AK124383	400123			NR_033877
HGNC:43688	LINC00550	13q21.33	BC042673	338862			NR_038878
HGNC:43691	LINC00551	13q33.3	AK098538	283483			
HGNC:43692	LINC00552	13q34	AK057435	100130386	ENSG00000279770		NR_028064
HGNC:43697	LINC00554	13q32.3	BC068276				
HGNC:43699	LINC00555	13q33.1	AF339825				
HGNC:43700	LINC00556	13q12.11	AF086361				
HGNC:43701	LINC00557	13q32.1	AF339811				
HGNC:43702	LINC00558	13q14.3	BC041877	100861552	ENSG00000261517		
HGNC:43703	LINC00559	13q31.3	BC043442				
HGNC:43704	LINC00560	13q31.2	BC043278				
HGNC:43705	LINC00561	13q22.2	BC034981				
HGNC:43706	LINC00562	13q14.2	AK123146				
HGNC:43707	LINC00563	13q14.13	U79295				
HGNC:43708	LINC00564	13q31.1	BC039360				
HGNC:43709	LINC00565	13q34	AK092053				
HGNC:43710	LINC00566	13q12.12	BC038727				
HGNC:43711	LINC00567	13q34	BC035264	283486			
HGNC:43717	LINC00570	2p25.1	BX102688, AI493106		ENSG00000224177	20887892	
HGNC:43721	LINC00571	13q13.3	BM023585, BF887755		ENSG00000223685		
HGNC:43722	LINC00572	13q12.3	BC039674		ENSG00000224405		
HGNC:21598	LINC00574	6q27	AK023224	80069	ENSG00000231690		NR_026780
HGNC:21342	LINC00575	4q21.23	AY316301	439934	ENSG00000231782	15218245	NR_024087
HGNC:43807	LINC00578	3q26.32	BX101293	100505566	ENSG00000228221		
HGNC:43840	LINC00581	6p22.3	BX118339	100874531		22245136	NR_103790
HGNC:43842	LINC00582	1q42.2		100287814	ENSG00000229228		NR_034037
HGNC:31437	LINC00583	9p23		100113404	ENSG00000205636		NR_038194
HGNC:31372	LINC00587	9q31.1	BC038565	414319	ENSG00000204250		
HGNC:24494	LINC00588	8q12.1		26138	ENSG00000215117	11230166	NR_026772
HGNC:32299	LINC00589	8p12	BC003524, BC014230	619351	ENSG00000251191		NR_026765
HGNC:27474	LINC00592	12q13.13	BC039104	283404	ENSG00000258279		NR_027358
HGNC:32382	LINC00593	15q23	BC031958	414926	ENSG00000259703	12477932	
HGNC:32358	LINC00594	15q15.1	AI278850	641314	ENSG00000259714		
HGNC:31430	LINC00595	10q22.3	AA256890, BQ082446, BQ070817		ENSG00000227136		
HGNC:23167	LINC00596	14q11.2	BX161431, CR615368	414767	ENSG00000259334		
HGNC:1193	LINC00597	15q23-q24	AL109679	81698			
HGNC:42770	LINC00598	13q14.11		646982	ENSG00000215483	12764377	NR_024505
HGNC:27231	LINC00599	8p23.1	AF052108	157627	ENSG00000253230	8619474, 9110174	NR_024281
HGNC:43916	LINC00601	10q26.2	BF215388		ENSG00000235180		
HGNC:43917	LINC00602	6q27	BC110806	441177			NR_027284
HGNC:43918	LINC00603	5p13.1	BG181872, BX100325		ENSG00000250048		
HGNC:43924	LINC00604	5p13.1	AI539454, BX115904		ENSG00000250585		
HGNC:43928	LINC00605	14q32.32	BC035396	100131366	ENSG00000251533		NR_033938
HGNC:43931	LINC00606	3p25.3	AK093398	285370	ENSG00000226567		NR_027102
HGNC:43944	LINC00607	2q35	AK091865	646324	ENSG00000235770		NR_037195
HGNC:27179	LINC00608	2q35	BC033552	151300	ENSG00000236445	12477932	
HGNC:43960	LINC00609	14q13.2-q13.	DN993271		ENSG00000257585		
HGNC:23262	LINC00610	11p13	AK127155	399879			
HGNC:28621	LINC00612	12p13.31	BC028195	253128	ENSG00000214851	12477932	NR_034140
HGNC:44060	LINC00613	4q28.3	AW237894	100507528	ENSG00000248330		
HGNC:30165	LINC00614	10p12.1		645587	ENSG00000236983		

HGNC:26343	LINC00615	12q21.33	AK058107	439916	ENSG00000196243		NR_038868
HGNC:44065	LINC00616	4q28.3		641365	ENSG00000248307		NR_037866
HGNC:20110	LINC00618	14q32.2	AA055628	145249	ENSG00000225163		
HGNC:31657	LINC00619	10q11.21	BC017939	414260	ENSG00000204187		NR_033923
HGNC:44223	LINC00620	3p25.1	BC039529	285375	ENSG00000224514	23478628, 21368711	NR_027103
HGNC:44227	LINC00621	13q12.12	AK091626		ENSG00000262619		
HGNC:44251	LINC00622	1p12	AK123168, BC015390	644242	ENSG00000260941		NR_036540
HGNC:44252	LINC00623	1q21.1		728855	ENSG00000226067		
HGNC:44254	LINC00624	1q21.1		100289211	ENSG00000278811		
HGNC:44257	LINC00626	1q24.2	BC001841	79100	ENSG00000225826		NR_024160
HGNC:27008	LINC00628	1q32.1	BC015457	127841		12477932	NR_027022
HGNC:44262	LINC00629	Xq26.3	BQ374946	100506757	ENSG00000227060		NR_038998
HGNC:44263	LINC00630	Xq22.1	BC032741, BC040959,	100287765	ENSG00000223546		NR_038988
HGNC:27865	LINC00632	Xq27.1	AK054978	286411	ENSG00000203930	24833871	
HGNC:44269	LINC00633	Xq26.3	AI015085, AY730280	100129515	ENSG00000224107		NR_033941
HGNC:27930	LINC00634	22q13.2		339674	ENSG00000205704	12477932	NR_024355
HGNC:27184	LINC00635	3q13.12	BC045586	151658	ENSG00000241469	12477932	NR_015414
HGNC:27702	LINC00636	3q13.12		285205	ENSG00000240423	12477932	NR_015394
HGNC:27069	LINC00637	14q32.33		145216	ENSG00000258735		NR_038436
HGNC:28325	LINC00638	14q32.33		196872	ENSG00000258701	12477932	NR_024396
HGNC:27502	LINC00639	14q21.1	AK125018, AK127318,	283547	ENSG00000259070		NR_039982
HGNC:44291	LINC00640	14q22.1		283553	ENSG00000258479		NR_038358
HGNC:27511	LINC00641	14q11.2	AK092333, AK093962	283624	ENSG00000258441		NR_038970
HGNC:44293	LINC00642	14q32.11	BC036259, BX101323	400238	ENSG00000233208		NR_033986
HGNC:44296	LINC00643	14q23.2		646113	ENSG00000186369		NR_015358
HGNC:44297	LINC00644	14q23.2	AW292481	101954204	ENSG00000259142		NR_104063
HGNC:44299	LINC00645	14q12	BC042069, BX538073,	100505967	ENSG00000258548		NR_039992
HGNC:44302	LINC00648	14q21.3	AK056212, CA435241	100506433	ENSG00000259129		NR_039996
HGNC:44305	LINC00649	21q22.11	DA374118	100506334	ENSG00000237945	26421281	NR_038883
HGNC:25003	LINC00652	20p11.23	AF161557, BC029555	29075	ENSG00000179935	11042152	NR_026883
HGNC:27154	LINC00654	20p12.3	BC067900	149837	ENSG00000205181		NR_015406
HGNC:27304	LINC00656	20p11.21	AF258576, CA748604,	200261	ENSG00000233746		NR_034149
HGNC:44315	LINC00658	20p12.3	BC035192	100507629	ENSG00000226995		NR_038239
HGNC:44316	LINC00659	20q13.33	DA633805, DB320598	100652730	ENSG00000228705		NR_046224
HGNC:27002	LINC00661	19p13.12	AI184190, CR749400	126536	ENSG00000205396	12477932	NR_026828
HGNC:27122	LINC00662	19q11	AK311452, BC045720	148189		12477932	NR_027301
HGNC:28609	LINC00663	19p13.11	AK289657, BC034236,	284440		12477932	NR_026956
HGNC:44319	LINC00664	19p12		400680	ENSG00000268658		NR_037194
HGNC:44323	LINC00665	19q13.12	AI282277, BC041949,	100506930	ENSG00000232677		NR_038278
HGNC:27906	LINC00667	18p11.31		339290	ENSG00000263753	8619474, 9110174	NR_015389
HGNC:44328	LINC00668	18p11.31		400643	ENSG00000265933		NR_034100
HGNC:44338	LINC00670	17p12	BC122870, DB255288	284034	ENSG00000179136		NR_034144
HGNC:44339	LINC00671	17q21.31	AK055784, BC122868,	388387	ENSG00000213373		NR_027254
HGNC:44353	LINC00672	17q12	AK090935, BC009632	100505576	ENSG00000263874		NR_038847
HGNC:44354	LINC00673	17q24.3	AK000477, AK129763	100499467		23040067	NR_036488
HGNC:44355	LINC00674	17q24.2	BC045718, DA232946	100499466	ENSG00000237854		NR_027418
HGNC:44356	LINC00675	17p13.1-p12	BC015790	100289255	ENSG00000263429		NR_036581
HGNC:44394	LINC00676	13q34	AW816149		ENSG00000234854		
HGNC:20121	LINC00677	14q32.32	AW182802	254299	ENSG00000259717		
HGNC:44413	LINC00678	11p14.1	CD176172, CD049147		ENSG00000254934		
HGNC:44417	LINC00680	6p11.2	BC011838, BC017330	106660612	ENSG00000215190		
HGNC:44423	LINC00681	8p23.1	BM725196, BM678210		ENSG00000255494		
HGNC:44466	LINC00682	4p13	BC025350		ENSG00000245870		
HGNC:44467	LINC00683	18q23	AK094957	400660	ENSG00000266256		

HGNC:44268	LINC00684	Xq13.1		100129407	ENSG00000275520		NR_034116
HGNC:27560	LINC00685	Xp22.33 and	BC041934	283981	ENSG00000226179	12477932	
HGNC:16221	LINC00686	20q13.33	D80415	140865	ENSG00000237687		
HGNC:16194	LINC00687	20p12.2	BX092309	728450	ENSG00000228422		XR_109647
HGNC:27217	LINC00689	7q36.3		154822	ENSG00000231419	12477932	NR_024394
HGNC:44503	LINC00690	3p24.3	BC034913	100996597	ENSG00000233570		
HGNC:44514	LINC00691	3p24.2		152024	ENSG00000224074		
HGNC:27708	LINC00692	3p24.2	BC033370	285326	ENSG00000230891	12477932	NR_034055
HGNC:44526	LINC00693	3p24.1		645206	ENSG00000228214		
HGNC:44570	LINC00694	3p21.31		100506301	ENSG00000225873		
HGNC:34426	LINC00696	3p21.1	AK127958	100128378			NR_027331
HGNC:27720	LINC00698	3p14.2	BC039502, BC043407	285401	ENSG00000244342		NR_027104
HGNC:27422	LINC00700	10p15.3	AK097474	282980	ENSG00000234962		NR_040253
HGNC:44674	LINC00701	10p15.3	BC055423	399708	ENSG00000234556		NR_038884
HGNC:44676	LINC00702	10p15.1	DA655411, DB287388,	100652988	ENSG00000233117		
HGNC:44677	LINC00703	10p15.1	AK095699	100507059	ENSG00000224382		
HGNC:44678	LINC00704	10p15.1	BC048320	100216001	ENSG00000231298		NR_024475
HGNC:27874	LINC00705	10p15.1	BC104742	338588	ENSG00000225269	12477932	NR_015425
HGNC:44690	LINC00706	10p14	DA137858, DB484295	100652997			
HGNC:44691	LINC00707	10p14		100507127	ENSG00000238266		NR_038291
HGNC:44694	LINC00708	10p14	BC031880	100507143	ENSG00000232170		
HGNC:44700	LINC00709	10p14	AI761698, BX111793, [illegible]	100507163	ENSG00000230014		
HGNC:27386	LINC00710	10p14		254312	ENSG00000229240		NR_015413
HGNC:50281	LINC00824	8q24.21	BC009730	101927774	ENSG00000254275		
HGNC:44915	LINC00836	10p12.1	W37259, BX100192	101929052			NR_108067
HGNC:27436	LINC00837	10p12.1		100507605	ENSG00000235824		NR_038374
HGNC:44963	LINC00838	10p11.22	BC042457	100505583	ENSG00000261683		NR_038932
HGNC:28269	LINC00839	10q11.21		84856	ENSG00000185904	12477932	NR_026827
HGNC:44987	LINC00840	10q11.21		100506835	ENSG00000226808		NR_038268
HGNC:27430	LINC00841	10q11.21	AK093142	283033	ENSG00000233395		
HGNC:44989	LINC00842	10q11.22		643650			NR_033957
HGNC:45009	LINC00843	10q11.23	BC035067	102902672	ENSG00000178440		
HGNC:45031	LINC00844	10q21.1	BG618474, AB060916,	100507008	ENSG00000237949		
HGNC:45033	LINC00845	10q21.2	BX105353, AI792470	100507058	ENSG00000227244		
HGNC:45050	LINC00847	5q35.3	AK095170	729678	ENSG00000245060		NR_027183
HGNC:45092	LINC00850	Xq28	JX283354			23223008	
HGNC:43424	LINC00851	20p11.23		440757	ENSG00000237282		NR_034167
HGNC:29904	LINC00852	3p25.3	AF280797	84657	ENSG00000231177	12477932	
HGNC:43716	LINC00853	1p33	AI792909	100874253	ENSG00000224805	20887892	
HGNC:43658	LINC00854	17q21.31		100874261	ENSG00000236383	22196729	
HGNC:45111	LINC00856	10q22.3		100132987	ENSG00000230417		NR_038985
HGNC:45114	LINC00857	10q22.3		439990	ENSG00000237523		NR_038464
HGNC:27276	LINC00858	10q23.1		170425	ENSG00000229404		NR_038220
HGNC:45133	LINC00861	8q24.13		100130231	ENSG00000245164		NR_038446
HGNC:21901	LINC00862	1q32.1	BC040731	554279	ENSG00000203721		NR_040064
HGNC:45162	LINC00863	10q23.2	BC082979	677759	ENSG00000224914		
HGNC:45163	LINC00864	10q23.2		728218	ENSG00000228055		NR_046091
HGNC:45170	LINC00865	10q23.31		643529	ENSG00000232229		NR_038382
HGNC:45232	LINC00866	10q24.2	BM126639, CK818728,	100505561	ENSG00000227356		
HGNC:45265	LINC00867	10q26.11	AI733769, AA121564	100506126	ENSG00000232139		
HGNC:27562	LINC00868	17q25.2	BC038218	283994	ENSG00000267535	12477932	
HGNC:29050	LINC00869	1q21.1	AB007962	57234	ENSG00000277147	9455484	NR_046135
HGNC:27319	LINC00870	3p13		201617	ENSG00000243083		NR_038221
HGNC:47038	LINC00871	14q21.2	BG186771		ENSG00000258700		

HGNC:27706	LINC00877	3p13	AK097190	285286	ENSG00000241163		
HGNC:48566	LINC00879	3q11.2		255025	ENSG00000239589		
HGNC:27948	LINC00880	3q25.31	BC038760, BC040669	339894	ENSG00000243629	12477932	
HGNC:48567	LINC00881	3q25.31		100498859	ENSG00000241135		NR_034008
HGNC:48568	LINC00882	3q13.12		100302640	ENSG00000242759	24886442	
HGNC:48570	LINC00884	3q29		401106	ENSG00000233058		
HGNC:48571	LINC00885	3q29		401109	ENSG00000224652		
HGNC:48572	LINC00886	3q25.31		730091	ENSG00000240875		
HGNC:48574	LINC00887	3q29		100131551	ENSG00000214145	26400545	
HGNC:48575	LINC00888	3q27.1	AK057000	100505687	ENSG00000240024		NR_038301
HGNC:26410	LINC00889	Xq26.3	AK055234, DA392242	158696			
HGNC:48576	LINC00890	Xq23		401613	ENSG00000260802		
HGNC:48577	LINC00891	Xq13.1	BX276092	441501			
HGNC:48578	LINC00892	Xq26.3		100128420	ENSG00000233093		
HGNC:44265	LINC00893	Xq28	AK124040, AF050144	100131434	ENSG00000241769	10737977	NR_027455
HGNC:48579	LINC00894	Xq28		100272228	ENSG00000235703		
HGNC:48580	LINC00895	22q11.21	AK057431, BC104250	150185			
HGNC:26519	LINC00896	22q11.21	AK057137	150197			
HGNC:48581	LINC00898	22q13.31		400932	ENSG00000205634		
HGNC:48583	LINC00899	22q13.31		100271722	ENSG00000231711		
HGNC:27444	LINC00900	11q23.3		283143	ENSG00000246100		
HGNC:40352	LINC00901	3q13.31	BI464214, BC040587	100506724	ENSG00000242385	25435812	
HGNC:40351	LINC00903	3q13.31	DB446734		ENSG00000241397		
HGNC:44325	LINC00904	19q13.11	BX091932	100652909			NR_046222
HGNC:26334	LINC00905	19p13.12	BC031284, BC069223,	148231	ENSG00000167459		NR_024335
HGNC:27121	LINC00906	19q12	BX647231, BC039341	148145	ENSG00000267339		NR_027318
HGNC:44327	LINC00907	18q12.3	BC021928, BC040725,	284260	ENSG00000267586		NR_046174
HGNC:27599	LINC00908	18q23	AK093936, BC035336,	284276	ENSG00000263812		NR_015417
HGNC:44331	LINC00909	18q22.3		400657	ENSG00000264247		
HGNC:44361	LINC00910	17q21.31	BC035366	100130581	ENSG00000188825		NR_027412
HGNC:48596	LINC00911	14q31.3	AA905171, AK131026	100996280	ENSG00000259107		NR_102737
HGNC:48604	LINC00914	6p21.33	HF678991				
HGNC:48607	LINC00917	16q24.1		732275	ENSG00000168367		
HGNC:48610	LINC00919	16q12.1	AK098676	100505619	ENSG00000260268		NR_038233
HGNC:48611	LINC00920	16q21	AW015153, DA813001	100505865	ENSG00000246898		NR_046242
HGNC:26830	LINC00921	16p13.3	AK096958, DA532447	283876			NR_033904
HGNC:27545	LINC00922	16q21	BC037902, BC104446	283867	ENSG00000261742		NR_027755
HGNC:28088	LINC00923	15q26.2	AK025311, BC105717	91948	ENSG00000251209		NR_024172
HGNC:27081	LINC00924	15q26.2		145820	ENSG00000259134		
HGNC:27514	LINC00926	15q21.3		283663	ENSG00000247982		
HGNC:27522	LINC00927	15q25.1		283688	ENSG00000259361		
HGNC:27535	LINC00928	15q26.1		283761	ENSG00000259218		
HGNC:48615	LINC00929	15q12		503519	ENSG00000259150		
HGNC:48620	LINC00930	15q26.1		100144604	ENSG00000258647		
HGNC:48625	LINC00933	15q25.2		100506874	ENSG00000259728		
HGNC:27059	LINC00934	12q24.23	AK057497	144742			
HGNC:48628	LINC00935	12q13.12	AK131005, BC054859	255411	ENSG00000257987		
HGNC:27883	LINC00936	12q21.33		338758		12477932	NR_028138
HGNC:48629	LINC00937	12p13.31	BC073935	389634	ENSG00000226091		
HGNC:48630	LINC00938	12q12		400027			NR_028408
HGNC:48631	LINC00939	12q24.32		400084	ENSG00000249267		
HGNC:48634	LINC00940	12p13.33	AK316036, DA269488	100271702	ENSG00000235049		
HGNC:48635	LINC00941	12p11.21	AK289874, BC072678	100287314	ENSG00000235884		
HGNC:48636	LINC00942	12p13.33	AK090616, BG332174,	100292680	ENSG00000249628		

HGNC:48639	LINC00943	12q24.32		100507206	ENSG00000189238		
HGNC:48640	LINC00944	12q24.32	BC040060, DA048939	387895	ENSG00000256128		
HGNC:48643	LINC00945	21q22.11	BF204217		ENSG00000232539		
HGNC:28098	LINC00950	9p13.3	AF370382	92973		8619474, 9110174	
HGNC:48662	LINC00951	6p21.2	AK123643, BC132805	401260	ENSG00000204092	23872665	
HGNC:48668	LINC00954	2p24.1	BC068572, DB155534	400946	ENSG00000228784		NR_033875
HGNC:26644	LINC00955	4p16.3	AK092743	285492	ENSG00000216560		
HGNC:22332	LINC00957	7p13	BC014556	255031	ENSG00000235314		
HGNC:48671	LINC00958	11p15.2	AK001432	100506305	ENSG00000251381		
HGNC:48677	LINC00959	10q26.3		387723	ENSG00000237489		
HGNC:48710	LINC00960	3p12.3		401074	ENSG00000242516		
HGNC:27244	LINC00961	9p13.3		158376	ENSG00000235387		
HGNC:48716	LINC00963	9q34.11		100506190	ENSG00000204054	24691949	
HGNC:27226	LINC00964	8q24.13		157381	ENSG00000249816	12477932	
HGNC:28022	LINC00965	8p23.1		349196			
HGNC:48725	LINC00967	8q13.1		100505659	ENSG00000253138		
HGNC:48727	LINC00968	8q12.1		100507632	ENSG00000246430		
HGNC:48729	LINC00969	3q29	AK128346		ENSG00000242086		
HGNC:48730	LINC00970	1q24.2	BC041008		ENSG00000203601	23741487	
HGNC:48737	LINC00971	3p12.1		440970	ENSG00000242641		
HGNC:48826	LINC00972	7q21.11	BG220912	105375380	ENSG00000225128		
HGNC:48868	LINC00973	3q12.1		100506377	ENSG00000240476		
HGNC:27105	LINC00974	17q21.2		147093	ENSG00000226629	25476897	
HGNC:50610	LINC00976	8q24.21	BF691581, BF691786				
HGNC:48902	LINC00977	8q24.21		728724	ENSG00000250400		
HGNC:48664	LINC00982	1p36.32		440556	ENSG00000177133	23801869, 26334618	
HGNC:48911	LINC00987	12p13.31	AK126248	100499405	ENSG00000237248		
HGNC:48918	LINC00989	4q21.21	BC047610	100506035	ENSG00000250334		
HGNC:48943	LINC00992	5q23.1	BC045192	728342	ENSG00000248663		
HGNC:48948	LINC00993	10p11.21	H39762	101929520	ENSG00000235687		
HGNC:48949	LINC00994	3p14.1		100287879	ENSG00000189196		
HGNC:27800	LINC00996	7q36.1		285972	ENSG00000242258	12477932	
HGNC:48952	LINC00997	7p14.3	AK127989	401321			NR_036501
HGNC:48953	LINC00998	7q31.1	BC107860	401397	ENSG00000214194		
HGNC:38537	LINC00999	10p11.1	AK128746	399744			NR_024497
HGNC:38541	LINC01000	7q32.1		402483			NR_024368
HGNC:38540	LINC01001	11p15.5		100133161	ENSG00000230724		NR_028326
HGNC:38538	LINC01002	19p13.3		399844	ENSG00000282508		NR_028324
HGNC:48957	LINC01003	7q36.1	BC036622	100128822	ENSG00000261455		
HGNC:48961	LINC01004	7q22.3		100216546	ENSG00000228393		
HGNC:48963	LINC01005	7q11.21		100506050	ENSG00000228564		
HGNC:48971	LINC01006	7q36.3		100506380	ENSG00000182648		
HGNC:48973	LINC01007	7q22.1		100506527	ENSG00000233123		
HGNC:48978	LINC01010	6q23.2		154092	ENSG00000236700		
HGNC:33812	LINC01011	6p25.2		401232	ENSG00000244041		
HGNC:48986	LINC01012	6p22.1	BC035101	100507173			
HGNC:48987	LINC01013	6q23.2		100507254	ENSG00000228495		
HGNC:41353	LINC01014	3q26.32	BX105665	100874330	ENSG00000223941		
HGNC:48988	LINC01015	6p22.1		100507362	ENSG00000224582		
HGNC:48991	LINC01016	6p21.31		100507584	ENSG00000249346	26426411	
HGNC:50641	LINC01017	5p15.33	BQ428982	102467075	ENSG00000250716		
HGNC:27394	LINC01018	5p15.31		255167	ENSG00000250056	12477932	
HGNC:27742	LINC01019	5p15.33		285577	ENSG00000248118	12477932	
HGNC:27968	LINC01020	5p15.32		340094	ENSG00000215231	12477932	

HGNC:48995	LINC01021	5p14.1		643401	ENSG00000250337		
HGNC:48998	LINC01022	7q36.3	DB518621		ENSG00000232715		
HGNC:49004	LINC01023	5q21.3	BF972671	100652853			NR_046368
HGNC:49009	LINC01024	5q31.2		100505636	ENSG00000245146	26337085	NR_102739
HGNC:49014	LINC01028	17q24.3	BX647864		ENSG00000267603		
HGNC:49015	LINC01029	18q23	BC016792, BF183892		ENSG00000265843		
HGNC:49016	LINC01030	8q21.3	BU568440		ENSG00000253799		
HGNC:49017	LINC01031	1q31.2	BX100123		ENSG00000232077		
HGNC:49018	LINC01032	1q31.2	AW104394		ENSG00000234871		
HGNC:49019	LINC01033	5q11.2	BG181707		ENSG00000249069		
HGNC:49021	LINC01034	13q22.2	BG183202		ENSG00000224933		
HGNC:49022	LINC01035	1q31.1	BQ212312		ENSG00000226486		
HGNC:49024	LINC01036	1q31.1	BU171718		ENSG00000236030		
HGNC:49025	LINC01037	1q31.1	BG182723		ENSG00000231599		
HGNC:49026	LINC01038	13q31.1	AI799053		ENSG00000229011		
HGNC:49027	LINC01039	13q32.3	BI464824		ENSG00000234168		
HGNC:49028	LINC01040	13q31.3	BG192883		ENSG00000226037		
HGNC:49031	LINC01043	13q34	BX649107		ENSG00000260343		
HGNC:49032	LINC01044	13q34	BG722997		ENSG00000223626		
HGNC:49040	LINC01046	13q12.11	AW117237		ENSG00000233405		
HGNC:49041	LINC01047	13q31.2	BG183515		ENSG00000232225		
HGNC:49042	LINC01048	13q13.3	BX091314		ENSG00000230390		
HGNC:49043	LINC01049	13q31.3	BI825411	101927224	ENSG00000234384		
HGNC:49044	LINC01050	13q14.11	CD367180		ENSG00000271216		
HGNC:49046	LINC01052	13q21.32	AA889679		ENSG00000234767		
HGNC:49047	LINC01053	13q12.13		102723318	ENSG00000238169		
HGNC:49048	LINC01054	13q34	BG460330		ENSG00000229723		
HGNC:49049	LINC01055	13q14.13	BG431110		ENSG00000235366		
HGNC:49050	LINC01056	20q13.33	BC038718	100144597	ENSG00000237119		
HGNC:49057	LINC01057	1p21.3	BC030750	101928079	ENSG00000224081		
HGNC:49061	LINC01058	13q12.3	CF138692		ENSG00000225039		
HGNC:49074	LINC01059	11q24.1	CR617890, CV373778		ENSG00000255414		
HGNC:49081	LINC01060	4q35.2		401164	ENSG00000249378		
HGNC:49084	LINC01061	4q26		401149			
HGNC:49092	LINC01063	3q29		101929769	ENSG00000232065		
HGNC:49103	LINC01065	13q14.3	AW303400		ENSG00000237092		
HGNC:49104	LINC01066	13q12.3	AA644401		ENSG00000230403		
HGNC:49105	LINC01067	13q33.3	BF969615		ENSG00000236053		
HGNC:49106	LINC01068	13q31.1	AI138787		ENSG00000227676		
HGNC:49109	LINC01069	13q22.3	BI825933	101927176	ENSG00000236133		
HGNC:49110	LINC01070	13q34	CR627049	101928698	ENSG00000260102		
HGNC:49112	LINC01072	13q12.11	AI150015		ENSG00000236076		
HGNC:49114	LINC01073	13q13.1	DR731308		ENSG00000235822		
HGNC:49117	LINC01074	13q21.31	BF057195		ENSG00000227611		
HGNC:49118	LINC01075	13q21.31	AI028357		ENSG00000230142		
HGNC:49119	LINC01076	13q12.13	BG190398		ENSG00000225105		
HGNC:49120	LINC01077	13q14.2	AI769787		ENSG00000233456		
HGNC:49121	LINC01078	13q22.2	AA442466		ENSG00000223880		
HGNC:49122	LINC01079	13q12.2	BM503984		ENSG00000229609		
HGNC:49123	LINC01080	13q31.1	DA225206	101515984		25019275	NR_104138
HGNC:49124	LINC01081	16q24.1	CR737045, HY035025	101154687		24842713	NR_104139
HGNC:49125	LINC01082	16q24.1	AA399680, AI676062	100506542	ENSG00000269186		NR_103859
HGNC:27198	LINC01085	4p15.33	AK055518	152742	ENSG00000248698		
HGNC:49144	LINC01087	2q21.1	BC036114	101927994	ENSG00000224559		

HGNC:49148	LINC01088	4q21.21		100505875	ENSG00000249307		
HGNC:27886	LINC01089	12q24.31	BC038786	338799	ENSG00000212694	26252651	NR_002809
HGNC:49201	LINC01090	2q32.1	DA863615		ENSG00000231689	24080187	
HGNC:27721	LINC01091	4q28.1	BC053945	285419	ENSG00000249464	12477932	
HGNC:49218	LINC01093	4q35.1		100506229	ENSG00000249173		
HGNC:49219	LINC01094	4q21.21		100505702	ENSG00000251442		
HGNC:49220	LINC01095	4q31.22		100505545	ENSG00000248809		
HGNC:27739	LINC01096	4p15.33		285548	ENSG00000246095	12477932	
HGNC:27738	LINC01097	4p15.33	BC035722	285547			NR_034054
HGNC:27731	LINC01098	4q34.3	BC033326	285501	ENSG00000231171		NR_028342
HGNC:49222	LINC01099	4q34.3	BU071468	101928656	ENSG00000251504		
HGNC:49224	LINC01100	3q25.33		730109	ENSG00000242107		
HGNC:25923	LINC01101	2q14.2	AK027722	84931	ENSG00000280409		NR_027181
HGNC:27165	LINC01102	2q12.1		150568	ENSG00000235597		
HGNC:49225	LINC01103	2q12.1		101927360	ENSG00000234781		
HGNC:49226	LINC01104	2q11.2		150577	ENSG00000232084		
HGNC:26403	LINC01105	2p25.2		150622	ENSG00000232044	25392693	
HGNC:26769	LINC01106	2q13		151009	ENSG00000175772		
HGNC:49229	LINC01107	2q37.3		151171	ENSG00000225493		
HGNC:49234	LINC01108	6p23	AK056826		ENSG00000226673	22193719	
HGNC:49235	LINC01109	8q21.11	AK124684		ENSG00000271167		
HGNC:49237	LINC01111	8q21.11		101926978	ENSG00000254300		
HGNC:49245	LINC01114	2q12.1		284998	ENSG00000234177		
HGNC:49258	LINC01115	2p25.3		339822	ENSG00000237667		
HGNC:49259	LINC01116	2q31.1		375295	ENSG00000163364		
HGNC:49260	LINC01117	2q31.1			ENSG00000224577		
HGNC:49261	LINC01118	2p21		388948	ENSG00000222005		
HGNC:49262	LINC01119	2p21		100134259	ENSG00000239332		
HGNC:49265	LINC01120	2q21.1		389043	ENSG00000223631		
HGNC:49266	LINC01121	2p21		400952	ENSG00000205054		
HGNC:49267	LINC01122	2p16.1		400955	ENSG00000233723	25959816	NR_033873
HGNC:49269	LINC01123	2q13		440894	ENSG00000204588		
HGNC:49270	LINC01124	2q31.1		440925	ENSG00000222033		
HGNC:49272	LINC01125	2q11.2		728537	ENSG00000228486		
HGNC:49275	LINC01126	2p21	BC032412, CR738297	100129726			
HGNC:49292	LINC01127	2q11.2	DB124570, DB153872	100506328			
HGNC:49377	LINC01128	1p36.33		643837	ENSG00000228794		
HGNC:49444	LINC01132	1q42.3	AK125357	100506810	ENSG00000227630		
HGNC:49447	LINC01133	1q23.2		100505633	ENSG00000224259	25908174	NR_038849
HGNC:49449	LINC01134	1p36.32		100133612	ENSG00000236423		
HGNC:49450	LINC01135	1p32.1		100131060	ENSG00000234807		
HGNC:49452	LINC01136	1q32.1		730227	ENSG00000233791		
HGNC:49453	LINC01137	1p34.3		728431	ENSG00000233621		
HGNC:49454	LINC01138	1q21.2		388685	ENSG00000274020		
HGNC:27924	LINC01139	1q43		339535	ENSG00000215808	12477932	
HGNC:27922	LINC01140	1p22.3	AK123670	339524	ENSG00000267272	12477932	
HGNC:49455	LINC01141	1p36.12		339505	ENSG00000236963		
HGNC:49456	LINC01142	1q24.2		284688	ENSG00000224286		
HGNC:49458	LINC01143	2p13.3	BX088774		ENSG00000237751		
HGNC:49460	LINC01144	1p34.1	BC006119	400752			
HGNC:49467	LINC01146	14q31.3		283587	ENSG00000258867		
HGNC:39757	LINC01149	6p21.33		101929111	ENSG00000230174		
HGNC:49469	LINC01150	11p15.5	L38282, U62668	101927624	ENSG00000229671	8968746	
HGNC:49471	LINC01151	8q24.13	DB186869		ENSG00000253819		

HGNC:16752	LINC01152	17q24.3	D43770, DB072529		ENSG00000256124	8789441	
HGNC:49495	LINC01153	10q26.12		101927889	ENSG00000227143		XR_246197
HGNC:49504	LINC01154	12q13.13	AA082317		ENSG00000257477	24344320	
HGNC:49512	LINC01157	5q13.3				24381249	
HGNC:49513	LINC01158	2q12.1	AK096498	100506421	ENSG00000233639	24381249	
HGNC:49514	LINC01159	2q12.1	AK095498		ENSG00000229743	24381249	
HGNC:49525	LINC01160	1p13.2	BG742401	100129269	ENSG00000231346		
HGNC:49528	LINC01162	7p15.3	CN412322		ENSG00000232790		
HGNC:49530	LINC01163	10q26.2	AK124226	101927381			XR_242761
HGNC:49533	LINC01164	10q26.3		399827			NR_038365
HGNC:49534	LINC01165	10q26.3	AK097584	100128830	ENSG00000229081		XR_252837
HGNC:49535	LINC01166	10q26.3	AK095503	101927590	ENSG00000232903		XR_242766
HGNC:49536	LINC01167	10q26.3	BI753673		ENSG00000224758		
HGNC:49537	LINC01168	10q26.3		399829	ENSG00000240707		NR_046231
HGNC:49541	LINC01169	15q22.31	AK096135, BC016970	102723165	ENSG00000259471		
HGNC:49542	LINC01170	5q23.2	N72756, AA215598		ENSG00000253807		
HGNC:49545	LINC01173	2q37.1-q37.2	BF185848				
HGNC:49548	LINC01176	7p14.3	BM981427	100506516			
HGNC:49549	LINC01177	16p13.2	AA725682		ENSG00000261397		
HGNC:49556	LINC01179	4q32.3	BX648971	101928151	ENSG00000249500		
HGNC:49558	LINC01180	17q21.31	BC043289	101927017	ENSG00000266979		
HGNC:25533	LINC01181	8q22.3	AK001351	379034	ENSG00000250929		
HGNC:49564	LINC01182	4p15.33	DB054622	101929071	ENSG00000250634		
HGNC:49565	LINC01184	5q23.3	BX648677	644873	ENSG00000245937	24036268	NR_015360
HGNC:49572	LINC01185	2p16.1	AK122786	400957	ENSG00000228414		
HGNC:49573	LINC01186	Xp11.3	BC015977	101927574	ENSG00000236751		
HGNC:49575	LINC01187	5q35.1	AK055811	100507267	ENSG00000249601		
HGNC:49592	LINC01189	9p11.2		643648	ENSG00000232827		
HGNC:49595	LINC01191	2q14.1	AW016224	440900	ENSG00000234199	24440876	NR_034128
HGNC:37197	LINC01192	3q26.1	BX103208	647107	ENSG00000241369	15905330	NR_033945
HGNC:28003	LINC01193	15q11.2	BC047459	348120	ENSG00000258710	15905330	NR_040094
HGNC:37171	LINC01194	5p15.2		404663	ENSG00000248131	14871852	NR_033383
HGNC:49550	LINC01195	16p13.2	CR748468		ENSG00000261075		
HGNC:49597	LINC01197	15q26.2	BC040875	400456	ENSG00000248441		
HGNC:49598	LINC01198	13q14.13	BC046635	101929344	ENSG00000231817		NR_120426
HGNC:49632	LINC01201	Xq26.1	AA418797		ENSG00000228659		
HGNC:49633	LINC01202	5q34	BI755689			24463510	
HGNC:49634	LINC01203	Xp22.2		100133123	ENSG00000226985		
HGNC:49635	LINC01204	Xp11.3		101927528	ENSG00000229563		
HGNC:49636	LINC01205	3q13.13		401082	ENSG00000228980		
HGNC:49637	LINC01206	3q26.33		100996490	ENSG00000242512		
HGNC:49638	LINC01207	4q32.3		100505989	ENSG00000248771		
HGNC:49639	LINC01208	3q26.32		100505547	ENSG00000223715		
HGNC:49640	LINC01209	3q26.32	BC041880	101928684	ENSG00000228308		
HGNC:49642	LINC01210	3q22.3		100507274	ENSG00000239513		
HGNC:49648	LINC01213	3q25.1		101927992	ENSG00000244541		
HGNC:49650	LINC01214	3q25.1		101928022	ENSG00000243550		
HGNC:49651	LINC01215	3q13.12		101929623	ENSG00000271856		
HGNC:41418	LINC01216	4q24			ENSG00000250223		
HGNC:41419	LINC01217	4q24			ENSG00000251219		
HGNC:41420	LINC01218	4q24			ENSG00000251636		
HGNC:49653	LINC01219	11p15.5	BX092271		ENSG00000232987		
HGNC:49664	LINC01220	14q24.3		731223	ENSG00000259687		
HGNC:49671	LINC01221	1q32.1	BG613973		ENSG00000235492		

HGNC:49672	LINC01222	1q32.1	BC040869		ENSG00000233410		
HGNC:49676	LINC01224	19p12			ENSG00000269416		
HGNC:49677	LINC01225	1p35.2		149086	ENSG00000260386		
HGNC:49678	LINC01226	1p35.2		284551	ENSG00000223907		NR_027085
HGNC:49680	LINC01227	16q23.2		101928276	ENSG00000260737		
HGNC:49681	LINC01228	16q23.2	BM973870		ENSG00000261082	24493833	
HGNC:49682	LINC01229	16q23.2		101928248	ENSG00000260876		NR_104661
HGNC:49686	LINC01230	9p24.3	AK055761			24504737	
HGNC:49688	LINC01231	9p24.2	BC041456	101929247	ENSG00000236511		
HGNC:49755	LINC01232	13q32.3	AK123584	102725509		24522499	
HGNC:49756	LINC01233	19p12	DB454962	100128139	ENSG00000269364	24522499	
HGNC:49757	LINC01234	12q24.13		100506465	ENSG00000249550	24522499	NR_110026
HGNC:49769	LINC01235	9p23	AK123194	401492	ENSG00000270547		NR_033863
HGNC:49793	LINC01237	2q37.3		101927289	ENSG00000233806		
HGNC:49796	LINC01239	9p21.3	AK092601, AA205910	441389	ENSG00000234840		NR_038977
HGNC:49804	LINC01241	9p21.2	BC043546	101929582			
HGNC:49810	LINC01242	9p21.1		401497			NR_046204
HGNC:49814	LINC01243	9p21.1	BC040892	101929620	ENSG00000260720		
HGNC:49840	LINC01246	2p25.2	BX096693	102800315			NR_110498
HGNC:49841	LINC01247	2p25.2		101929390	ENSG00000227007		NR_110251
HGNC:49842	LINC01248	2p25.2	BX111928	102723818	ENSG00000224128		
HGNC:49843	LINC01249	2p25.2	AK307324, BC047589	727982	ENSG00000231532		NR_034134
HGNC:49844	LINC01250	2p25.3	AK095310	101927554	ENSG00000234423		
HGNC:49845	LINC01251	9p13.3	BX115224	101929688	ENSG00000233776		NR_109755
HGNC:27888	LINC01252	12p13.2		338817	ENSG00000247157	12477932	
HGNC:49870	LINC01254	18p11.22		101927350	ENSG00000260913		
HGNC:49871	LINC01255	18p11.21		101927433	ENSG00000267252		
HGNC:49895	LINC01256	4q28.3	BX088800		ENSG00000251398		
HGNC:26972	LINC01257	12q24.33	BC112214	116437	ENSG00000204603		
HGNC:49898	LINC01258	4p14		101928776	ENSG00000249534		
HGNC:49899	LINC01259	4p14	CR984336		ENSG00000249667		
HGNC:49900	LINC01260	20q13.12	BC000849	79015	ENSG00000132832		
HGNC:50275	LINC01262	4q35.2	BC092460	101928971	ENSG00000250739	24651478	
HGNC:50282	LINC01264	10q11.21	BE644626		ENSG00000229630	24672800	
HGNC:50297	LINC01265	5p13.1		101926904	ENSG00000249911		
HGNC:50309	LINC01266	3p26.3		101927215	ENSG00000224957		
HGNC:50320	LINC01267	3p25.1		101927565	ENSG00000251576		
HGNC:50323	LINC01268	6q21	BC037331	285758	ENSG00000227502	24821221	
HGNC:50325	LINC01269	14q24.2	DA437066		ENSG00000258689		
HGNC:27658	LINC01270	20q13.13	AK090605	284751	ENSG00000203999		
HGNC:50327	LINC01271	20q13.13		101927586	ENSG00000233077		
HGNC:50328	LINC01272	20q13.13		100506115	ENSG00000224397		
HGNC:50329	LINC01273	20q13.13		101927541	ENSG00000231742		
HGNC:50333	LINC01276	6p21.1	DQ141194		ENSG00000226917		
HGNC:50334	LINC01277	6q24.2		100507489	ENSG00000229017		
HGNC:28090	LINC01278	Xq11.1		92249	ENSG00000235437		
HGNC:50335	LINC01279	3q13.2	AK126393	100506621	ENSG00000272761		
HGNC:50336	LINC01280	2q35	AK307257	101928232	ENSG00000224391		
HGNC:50337	LINC01281	Xp11.4		286442	ENSG00000235304		
HGNC:50338	LINC01282	Xp11.4	BC043536	101927449	ENSG00000236747		
HGNC:50342	LINC01284	Xp11.22		101926971	ENSG00000230317		
HGNC:50344	LINC01285	Xq24		101928287	ENSG00000203650		NR_110393
HGNC:50351	LINC01287	7q36.2	BF514057		ENSG00000234722		
HGNC:50353	LINC01288	8p12	DB066514		ENSG00000254344		

HGNC:50354	LINC01289	8q12.3	BC037345	286184	ENSG00000253734		
HGNC:50356	LINC01290	16p13.13	BF853722, BI010826		ENSG00000260468		
HGNC:50358	LINC01291	2p12	CV382059		ENSG00000204792		
HGNC:50362	LINC01293	2p12		101927861	ENSG00000230836		
HGNC:48904	LINC01296	14q11.2	BC041856	642477	ENSG00000244306	25894381	
HGNC:50443	LINC01297	14q11.2	BF001177		ENSG00000274827		
HGNC:32292	LINC01298	8q22.1	AA469917	619344	ENSG00000253351		NR_046232
HGNC:27839	LINC01299	8q13.1	AK094960	286186	ENSG00000254081		
HGNC:50460	LINC01300	8q24.3		731779	ENSG00000253595		
HGNC:50464	LINC01301	8q12.1		100505532	ENSG00000251396		
HGNC:50472	LINC01304	2p25.3		100505964	ENSG00000237401		
HGNC:27690	LINC01305	2q31.1		285084	ENSG00000231453		
HGNC:50494	LINC01307	1p21.2	BG203915		ENSG00000231671		
HGNC:50497	LINC01309	13q33.1	AA421210		ENSG00000234551		
HGNC:50501	LINC01310	22q13.32	AK056844	100128946	ENSG00000205632		
HGNC:50503	LINC01311	22q11.21		100652736	ENSG00000260924		
HGNC:28525	LINC01312	6q23.2		154089	ENSG00000223586	12477932	
HGNC:50507	LINC01314	15q25.1		100996492	ENSG00000259417		
HGNC:50513	LINC01315	22q13.2	BC038245	102723775	ENSG00000229891		
HGNC:50523	LINC01317	2p22.3	BQ213083		ENSG00000203386	24833871	
HGNC:50524	LINC01318	2p22.3	DB027554		ENSG00000237790		
HGNC:50526	LINC01320	2p22.3	BX395998, BI759570		ENSG00000228262		
HGNC:50528	LINC01322	3q26.1	DA224933, DA156477		ENSG00000244128		
HGNC:50529	LINC01323	3q26.1	BG192479		ENSG00000241636		
HGNC:50530	LINC01324	3q26.1	BG221042		ENSG00000241767		
HGNC:50532	LINC01326	3q26.1	BG190199		ENSG00000244321		
HGNC:50533	LINC01327	3q26.1	BF059162		ENSG00000241669		
HGNC:50536	LINC01330	3q26.2	AK093355, BC043538	646168	ENSG00000244227		
HGNC:50538	LINC01331	5q13.3	DA145234		ENSG00000248673		
HGNC:50540	LINC01332	5q13.3	DA178531		ENSG00000250446		
HGNC:50539	LINC01333	5q13.3	BC043537	101929082	ENSG00000249343		
HGNC:50542	LINC01335	5q13.3	AI018361	102503429	ENSG00000248942		
HGNC:50543	LINC01336	5q13.3	DB344036		ENSG00000250889		
HGNC:50546	LINC01337	5q14.1	DB525610		ENSG00000251221		
HGNC:50547	LINC01338	5q14.2		102546175			NR_105016
HGNC:50549	LINC01339	5q14.3	AI674738	101929495	ENSG00000248555		
HGNC:50550	LINC01340	5q15	BQ432570	102546227	ENSG00000250331		
HGNC:49457	LINC01341	1q44		149134	ENSG00000227953		
HGNC:50551	LINC01342	1p36.33	BC028014	254099	ENSG00000223823		
HGNC:50553	LINC01343	1p34.3	BC042675	339442	ENSG00000237290		
HGNC:50554	LINC01344	1q25.3		400799	ENSG00000228918		
HGNC:50563	LINC01346	1p36.32		728716	ENSG00000233304		
HGNC:50566	LINC01347	1q43		731275	ENSG00000214837		
HGNC:50567	LINC01348	1q42	AK128489	731656			
HGNC:50568	LINC01349	1p21.2		100128787	ENSG00000230402		
HGNC:50575	LINC01350	1q25.3		101929093	ENSG00000228309		
HGNC:50577	LINC01351	1q31.1		101929120	ENSG00000237457		
HGNC:50578	LINC01352	1q41		101929730	ENSG00000238078		
HGNC:50580	LINC01353	1q32.1		100506775	ENSG00000231507		
HGNC:50581	LINC01354	1q42.2		100506795	ENSG00000231768		
HGNC:50584	LINC01355	1p36.12		100996511	ENSG00000261326		
HGNC:50587	LINC01356	1p13.2		100996702	ENSG00000215866		
HGNC:50589	LINC01358	1p32.1		101926925	ENSG00000230812		
HGNC:50591	LINC01359	1p31.3		101927084	ENSG00000226891		

HGNC:50593	LINC01360	1p31.1		101927295	ENSG00000233973		
HGNC:50595	LINC01361	1p31.1		101927498	ENSG00000236268		
HGNC:50596	LINC01362	1p31.1	BC043539		ENSG00000230817		
HGNC:50598	LINC01363	1q24.1		101928484	ENSG00000231605		
HGNC:50599	LINC01364	1p22.3		100505768	ENSG00000227290		
HGNC:50603	LINC01365	4q26		101927007	ENSG00000250772		
HGNC:27416	LINC01366	5q35.1		257358	ENSG00000235172		
HGNC:50608	LINC01370	20q12	BI792917	100505663	ENSG00000237767	23040067	NR_109936
HGNC:50623	LINC01372	7q11.21	BC017910	101929736	ENSG00000235475		
HGNC:50631	LINC01374	10q23.31	AI126223			24876753	
HGNC:50632	LINC01375	10q23.31	AK093219	101926924	ENSG00000226159		
HGNC:50637	LINC01376	2p24.1	AK123475	400945	ENSG00000236204		
HGNC:50642	LINC01377	5p15.33		102467074			
HGNC:50645	LINC01378	4q26	DB466276		ENSG00000236922		
HGNC:50653	LINC01381	2p23.3	AW515644		ENSG00000230452		
HGNC:50656	LINC01384	20p11.21		101929685	ENSG00000237396		
HGNC:50657	LINC01385	5q13.2	DB294021		ENSG00000251604		
HGNC:50659	LINC01386	5q13.2	AA969225		ENSG00000251324		
HGNC:44660	LINC01387	18p11.31		100130480	ENSG00000265944		
HGNC:50660	LINC01388	9p24.3	AW873619		ENSG00000231808		
HGNC:50661	LINC01389	1p33	BX102452		ENSG00000225762		
HGNC:50666	LINC01391	3q22.3	AK128202		ENSG00000244578		
HGNC:50668	LINC01392	7q31.2	DA452608		ENSG00000233607		
HGNC:50669	LINC01393	7q31.2	BC022431		ENSG00000225535		
HGNC:50670	LINC01394	6p25.3	AI698698				
HGNC:50674	LINC01395	11q24.3	BC038579	101929557			
HGNC:50675	LINC01396	4p16.2	AW195733		ENSG00000273396		
HGNC:50677	LINC01397	1p13.3	DA779101		ENSG00000258673		
HGNC:50678	LINC01398	1p33	BC021746	101929651	ENSG00000224863		
HGNC:50680	LINC01399	22q12.3	CB111670		ENSG00000233080		
HGNC:50681	LINC01400	9p13.2	AL559860		ENSG00000232920		
HGNC:50685	LINC01402	Xq24	AA360663	104797536	ENSG00000228139		NR_126557
HGNC:50688	LINC01405	12q24.11	AJ276555	100131138	ENSG00000185847		
HGNC:50702	LINC01410	9q13	CR627148		ENSG00000238113		
HGNC:50703	LINC01411	5q35.2	BM051440	101928176	ENSG00000249306	25422238	NR_125806
HGNC:50704	LINC01412	2q22.3		101928455	ENSG00000232606		
HGNC:50705	LINC01413	15q21.3		101928611	ENSG00000260172		
HGNC:50709	LINC01415	18q21.2	AK127787	100132501	ENSG00000267325		
HGNC:51645	LINC01416	18q21.2		101927273	ENSG00000260930		NR_110755
HGNC:50712	LINC01419	8q21.13	BC038578		ENSG00000253898		
HGNC:50713	LINC01420	Xp11.21	BC048131	550643	ENSG00000204272		
HGNC:50728	LINC01422	22q12.1		101929539	ENSG00000223704		
HGNC:50732	LINC01423	21q22	AI752367, DA464432	102724678	ENSG00000231231		
HGNC:40558	LINC01424	21q22.3		101928689	ENSG00000236519		
HGNC:50733	LINC01425	21q21.1		101927821	ENSG00000233997		
HGNC:50734	LINC01426	21q22.12		100506385	ENSG00000234380		
HGNC:50737	LINC01427	20p11.22		101929663	ENSG00000225321		
HGNC:50738	LINC01428	20p12.3		101929265	ENSG00000228888		
HGNC:50741	LINC01429	20q13.2		101927678	ENSG00000227964		
HGNC:50742	LINC01430	20q13.12		101927242	ENSG00000237907		
HGNC:50744	LINC01431	20p11.21		100505683	ENSG00000232645		
HGNC:50745	LINC01432	20p11.22		100270679	ENSG00000234435		
HGNC:50746	LINC01433	20p13		728228	ENSG00000230176	25820656	NR_033917
HGNC:50753	LINC01435	10q25.1	BX116650, AA406208		ENSG00000229981	24932682	

HGNC:50754	LINC01436	21q22.12		100996609	ENSG00000231106	24932682	
HGNC:50757	LINC01438	4q25	DA725631		ENSG00000249519		
HGNC:50762	LINC01440	20q13.2	BX107747	102723578	ENSG00000235166		
HGNC:50763	LINC01441	20q13.2		101927796	ENSG00000224008		
HGNC:50764	LINC01442	13q21.2	AK097816, AA393276		ENSG00000227510		
HGNC:50768	LINC01443	18p11.21		400644	ENSG00000266554		
HGNC:50769	LINC01444	18p11.21		101927642	ENSG00000264301		
HGNC:50771	LINC01445	7p11.2	BF966467	102723605	ENSG00000231427		
HGNC:50773	LINC01446	7p12.1		401337	ENSG00000205628		
HGNC:50783	LINC01447	7p12.3		101929086	ENSG00000236078		
HGNC:50790	LINC01448	7p14.1		101928795	ENSG00000238284		
HGNC:50791	LINC01449	7p14.1		101928773	ENSG00000224017		
HGNC:50792	LINC01450	7p14.1		101928744	ENSG00000232458		
HGNC:48599	LINC01451	9q34.3	AY129027, CA503198	401561			
HGNC:50545	LINC01455	5q14.1	DB088210		ENSG00000250888		
HGNC:50846	LINC01456	Xp22.13	BE792552		ENSG00000225882		
HGNC:50858	LINC01460	2p23.2	AK124439	100129995	ENSG00000205334		
HGNC:50859	LINC01461	1p22.3	DA091614				
HGNC:26364	LINC01465	12q14.1	AK098456	283416	ENSG00000221949		
HGNC:50911	LINC01467	14q31.1		101928559	ENSG00000258977		
HGNC:50913	LINC01468	10q21.1		101928687	ENSG00000231131	25045670	
HGNC:51105	LINC01470	5q33.1		101927134	ENSG00000249484		
HGNC:51106	LINC01471	3q21.3		101927149	ENSG00000239921		
HGNC:51109	LINC01473	2q32.1		101927217	ENSG00000237877		
HGNC:51111	LINC01474	9q21.13		101927258	ENSG00000236849		
HGNC:51113	LINC01475	10q24.2		101927324	ENSG00000257582		
HGNC:51117	LINC01476	17q23.1		101927728	ENSG00000265313		
HGNC:51119	LINC01477	18q12.3		101927900	ENSG00000261715		
HGNC:51121	LINC01478	18q12.3		101927921	ENSG00000267337		
HGNC:51123	LINC01479	12q15		101927922	ENSG00000255772		
HGNC:51124	LINC01480	19q13.2		101927931	ENSG00000270164		
HGNC:51126	LINC01481	12q15		101928062	ENSG00000257613		
HGNC:51128	LINC01482	17q24.2		101928104	ENSG00000267659		
HGNC:51130	LINC01483	17q24.3		101928122	ENSG00000227517		
HGNC:51136	LINC01484	5q35.2		101928136	ENSG00000253686		
HGNC:51138	LINC01485	5q35.2		101928154	ENSG00000254211		
HGNC:51137	LINC01486	12q24.11		101928138	ENSG00000256560		
HGNC:51139	LINC01487	3q25.2		101928190	ENSG00000241336		
HGNC:51144	LINC01488	11q13.3		101928292			NR_120542
HGNC:51145	LINC01489	12p12.3		101928340	ENSG00000255727		
HGNC:51146	LINC01490	12q21.31		101928420	ENSG00000257741		
HGNC:51148	LINC01491	15q21.1		101928442	ENSG00000259572		
HGNC:51149	LINC01492	9q31.1		101928496	ENSG00000225564		
HGNC:51150	LINC01493	11p12		101928536	ENSG00000254562		
HGNC:51152	LINC01494	2q35		101928537	ENSG00000228135		
HGNC:51161	LINC01495	11p14.3		102723378	ENSG00000255323		
HGNC:51162	LINC01496	Xp11.22		102723426	ENSG00000234766		
HGNC:51163	LINC01497	17q24.3		102723487	ENSG00000237560		
HGNC:51164	LINC01498	12q23.3		102723562	ENSG00000247213		
HGNC:51165	LINC01499	11p12		102723644	ENSG00000255171		
HGNC:51166	LINC01500	14q23.1		102723742	ENSG00000258583		
HGNC:27988	LINC01501	9q22.2	BC040625	340515	ENSG00000229613	12477932	NR_034157
HGNC:51183	LINC01502	9q34.3	AK098241	100130954	ENSG00000237339		NR_034016
HGNC:51184	LINC01503	9q34.11		100506119	ENSG00000233901	25045670	NR_120685

HGNC:51185	LINC01504	9q21.13	AK095210	100507540	ENSG00000225434		NR_110952
HGNC:51186	LINC01505	9q31.2	AK093363	100996590	ENSG00000234229		NR_104145
HGNC:51187	LINC01506	9q21.11		101927015	ENSG00000234506		NR_109769
HGNC:51189	LINC01507	9q21.31		101927477	ENSG00000230945	26328256	NR_121212
HGNC:51190	LINC01508	9q22.2		101927873	ENSG00000231107		NR_109795
HGNC:51193	LINC01509	9q31.2		101928720	ENSG00000226825		NR_121581
HGNC:51196	LINC01510	7q31.2		100996266	ENSG00000231210		
HGNC:51200	LINC01511	5p15.33	BC034612	100506791	ENSG00000250584	25085781	
HGNC:51201	LINC01512	6p21.1	AK095548	100132354		25089627, 23040067	NR_024478
HGNC:51202	LINC01513	5p15.2	BM975707		ENSG00000246016	25089627	
HGNC:51207	LINC01514	10q24.31	BC038784	101927396	ENSG00000237579		NR_120620
HGNC:51210	LINC01515	10q21.3		101928913	ENSG00000228065		NR_120647
HGNC:51211	LINC01516	10p12.1	BC042983	101929025	ENSG00000231422		NR_120649
HGNC:51212	LINC01517	10p12.1	HY052409	101929218	ENSG00000232624		NR_120652
HGNC:51216	LINC01518	10q11.21	AK123067	101929397	ENSG00000233515		NR_120659
HGNC:51217	LINC01519	10q23.1	AK097624	101929624	ENSG00000237267		NR_120667
HGNC:51218	LINC01520	10q23.1	AL117638	101929684	ENSG00000230962		NR_120671
HGNC:26029	LINC01521	22q12.2	BC004550	54944	ENSG00000213888		NR_120386
HGNC:51224	LINC01522	20q13.13		101927457	ENSG00000237423		NR_110027
HGNC:51225	LINC01523	20q13.13		101060004	ENSG00000229522		NR_109942
HGNC:51228	LINC01524	20q13.2	CD109474, BC045652	101927700	ENSG00000234948		NR_110038
HGNC:51264	LINC01525	1p12	BX109912, AW590930, AA909723		ENSG00000235202	25045670	
HGNC:51265	LINC01526	6q14.1	BC038576	101928770	ENSG00000224995	25045670	
HGNC:51266	LINC01527	1q21.3	BC042056	101927988	ENSG00000224308	25045670	XR_241125
HGNC:51268	LINC01529	19q13.12	AK055260	644050	ENSG00000225872		NR_104176
HGNC:44320	LINC01530	19q13.41	AK054965, CA867516,	729975			NR_034159
HGNC:51270	LINC01531	19q13.12	AK128099	100128682	ENSG00000205786		NR_040046
HGNC:51272	LINC01532	19q12	AK055100	100505835	ENSG00000267014		NR_040036
HGNC:51274	LINC01533	19q13.11		100996665	ENSG00000267779		NR_110673
HGNC:51281	LINC01534	19q13.12		101927621	ENSG00000225975		NR_110708
HGNC:51282	LINC01535	19q13.12		101927667	ENSG00000226686		NR_110720
HGNC:51301	LINC01537	11q13.4	AL832797	101928555	ENSG00000227467	25243407	
HGNC:51306	LINC01538	18q22.1	BC042493	400654	ENSG00000266952		NR_033983
HGNC:51307	LINC01539	18q21.2	AK057336, AK058169	100505474	ENSG00000267712		NR_040025
HGNC:51309	LINC01541	18q22.3	BX648957	100505776	ENSG00000260676		NR_038325
HGNC:51310	LINC01543	18q11.2	HY185322	100506787	ENSG00000263862		NR_110762
HGNC:51312	LINC01544	18q21.33	AK054836	100996669	ENSG00000260440		NR_103449
HGNC:17986	LINC01545	Xp11.3	BC038573	724087	ENSG00000204904		NR_046101
HGNC:27336	LINC01546	Xp22.33		100129464	ENSG00000228459		NR_038428
HGNC:15707	LINC01547	21q22.3	AY040088	84536	ENSG00000183250	11707072	NR_027128
HGNC:1296	LINC01548	21q22.11		728409	ENSG00000229086		NR_024102
HGNC:1277	LINC01549	21q21.1		100505929	ENSG00000232560		NR_037585
HGNC:20111	LINC01550	14q32.2	BC043585	388011	ENSG00000246223		NR_015430
HGNC:19828	LINC01551	14q11.2		387978	ENSG00000186960		NR_026731
HGNC:23524	LINC01553	10q21.3	BC038741	283025	ENSG00000235931	12477932	NR_024340
HGNC:24687	LINC01554	5q15	AY168789	202299	ENSG00000236882		NR_026936
HGNC:26647	LINC01555	1p22.3	AK092806	439927	ENSG00000180869		NR_027379
HGNC:21195	LINC01556	6p22.1		729583	ENSG00000204709		NR_103538
HGNC:21235	LINC01558	6q27		26238	ENSG00000146521	10382971	NR_026773
HGNC:26598	LINC01559	12p13.1	AK091129	283422	ENSG00000180861		NR_036555
HGNC:27333	LINC01560	Xp11.23	BC025179	203414	ENSG00000196741	12477932	NR_126059
HGNC:31365	LINC01561	10q26.12	AK094721	404216	ENSG00000177234		NR_103717
HGNC:51335	LINC01562	1p32.3	AK127900		ENSG00000203356	25280944	
HGNC:51343	LINC01563	17p11.2	AB593144	101060544	ENSG00000236819		NR_110895

HGNC:51361	LINC01564	6p12.1		101927171	ENSG00000235899	25307116	NR_125841
HGNC:17099	LINC01565	3q21	AF008192	23434	ENSG00000198685	9307271	
HGNC:27555	LINC01566	16p11.1	BC038774, BC045579	283914	ENSG00000259841		NR_027079
HGNC:51367	LINC01567	16p12.1	AK127191	400511	ENSG00000224310		NR_122072
HGNC:51371	LINC01568	16q22.3	BC036646	100506172	ENSG00000258779		NR_038234
HGNC:51380	LINC01569	16p13.3	AK094828	100507501	ENSG00000262468		NR_039999
HGNC:51382	LINC01570	16p13.3	BI825594, DB449025	101926950	ENSG00000260338		NR_110902
HGNC:51384	LINC01571	16q12.1	AI050873	101927364	ENSG00000260057		NR_110916
HGNC:51385	LINC01572	16q22.2		101927957	ENSG00000261008		NR_126330
HGNC:51388	LINC01574	5q35.2		102577424	ENSG00000248859	23040067	NR_108030
HGNC:48626	LINC01578	15q26.1		100507217	ENSG00000272888		NR_037601
HGNC:27519	LINC01579	15q26.1-q26.	BC037497	283682	ENSG00000258754		
HGNC:51414	LINC01580	15q26.2	BC021741	101927129	ENSG00000258785		NR_120322
HGNC:51415	LINC01581	15q26.2	BC043440	101927112	ENSG00000259724		NR_120320
HGNC:51416	LINC01582	15q26.3		101927332	ENSG00000259611		NR_120325
HGNC:51425	LINC01583	15q25.2	BC043257	101929690	ENSG00000259518		NR_120367
HGNC:51426	LINC01584	15q25.3	BX116292	101929701	ENSG00000260477		NR_120368
HGNC:51432	LINC01585	15q26.1	BC038778	101929765	ENSG00000245479		NR_120371
HGNC:51434	LINC01586	15q26.1	AK054749	101929743	ENSG00000249487		NR_120369
HGNC:13716	LINC01587	4p16	D82070	10141	ENSG00000082929	9016955	NR_126517
HGNC:27503	LINC01588	14q22.1	AK090420	283551	ENSG00000214900		NR_126496
HGNC:51520	LINC01589	22q13.31	BC045163	100506737	ENSG00000238120	25482444	
HGNC:21404	LINC01590	6q15-q16.1	AK021476	63914			NR_026784
HGNC:27819	LINC01591	8q24.22		286094	ENSG00000254083	12477932	NR_026706
HGNC:51557	LINC01592	8q13.2		100505718	ENSG00000253658		NR_039986
HGNC:51583	LINC01593	2q12.3			ENSG00000231221		
HGNC:51584	LINC01594	2q12.3	CR748429	102724774	ENSG00000225328		
HGNC:51586	LINC01595	19q13.33	BP420239, AV687190		ENSG00000259108		
HGNC:51591	LINC01596	4q35.2	BX094246, AI806912		ENSG00000250666		
HGNC:51594	LINC01597	20q11.1	AK127732		ENSG00000205611		
HGNC:51597	LINC01598	20q11.1	BX100171, AA470003, AW342060		ENSG00000231934		
HGNC:27285	LINC01599	14q21.3	AF390030	196913	ENSG00000168260		NR_102736
HGNC:21600	LINC01600	6p25.2	AK056496	154386	ENSG00000164385		NR_131168
HGNC:51651	LINC01601	18q12.3	BX119171	105667213			NR_131768
HGNC:51634	LINC01602	8q12.1	BC040611	100505477	ENSG00000205293	15223130	NR_130934
HGNC:51652	LINC01603	8q13.2		100505739	ENSG00000253479		NR_110433
HGNC:51654	LINC01605	8p11.23		100507420	ENSG00000253161		NR_121620
HGNC:51656	LINC01606	8q12.1		100507651	ENSG00000253301		NR_038235
HGNC:51660	LINC01607	8q21.13		101927067	ENSG00000272138		NR_125410
HGNC:51662	LINC01608	8q23.2		101927459	ENSG00000253877		NR_125416
HGNC:51663	LINC01609	8q23.2-q23.3		101927487	ENSG00000253103		NR_125418
HGNC:51791	LINC01611	6q14.3		105377880	ENSG00000231776	25364816	
HGNC:51837	LINC01612	4q33		101928223	ENSG00000250266	26045391	NR_125889
HGNC:51842	LINC01613	9q33.2		106146149	ENSG00000261432	26269497	NR_132393
HGNC:51847	LINC01614	2q35		105373869	ENSG00000230838	25116943	NR_132383
HGNC:51898	LINC01615	6q27	CB123670	101929484	ENSG00000223485		NR_132622
HGNC:51900	LINC01616	11p14.1	AK095844	106456574	ENSG00000261340	26318290	NR_132652
HGNC:30442	LINC01617	8q21.11	AA861243	101926947		26328256	
HGNC:27195	LINC01618	4q12		152578	ENSG00000250302	26328256	NR_040106
HGNC:27409	LINC01619	12q21.33		256021	ENSG00000257242		NR_132339
HGNC:16195	LINC01620	20q13.12		140834	ENSG00000168746		NR_132342
HGNC:14109	LINC01621	6q14.1		89758	ENSG00000235357		NR_131786
HGNC:27768	LINC01622	6p25.3		285768		12477932	NR_027115
HGNC:52050	LINC01623	6p22.1		401242	ENSG00000225595		NR_033379

HGNC:52051	LINC01624	6q27		401289	ENSG00000227508		NR_104177
HGNC:52052	LINC01625	6q24.1		645434	ENSG00000238099		NR_033919
HGNC:52257	LINC01626	6q13		102724000	ENSG00000223765		NR_121615
HGNC:52258	LINC01627	9p13.2			ENSG00000224648	26393913	
HGNC:52259	LINC01628	2p14		102724321	ENSG00000232688	26393913	NR_110600
HGNC:52260	LINC01629	14q24.3		105370578	ENSG00000258602	26400545	
HGNC:49089	LINCMD1	6p12.2	BG461844	101154644	ENSG00000225613		
HGNC:27368	LINGO1-AS1	15q24.3	BC042092	253044	ENSG00000259666	12477932	NR_045123
HGNC:51423	LINGO1-AS2	15q24.3	BC038758	101929478	ENSG00000259281		NR_120361
HGNC:48589	LIPE-AS1	19q13.2	AK096849, BM974950	100996307	ENSG00000213904		NR_073179
HGNC:50493	LLPH-AS1	12q14.3	DA010836		ENSG00000239335		
HGNC:44477	LMCD1-AS1	3p26.1		100288428	ENSG00000227110		NR_033378
HGNC:50469	LMF1-AS1	16p13.3		101929387	ENSG00000260439		
HGNC:40738	LMLN-AS1	3q29			ENSG00000232832		
HGNC:50277	LMO7-AS1	13q22.2	AW188034	101927155	ENSG00000261105		
HGNC:49790	LMO7DN-IT1	13q22.2	AI825938		ENSG00000223458		
HGNC:40345	LNX1-AS1	4q12			ENSG00000250930		
HGNC:41450	LNX1-AS2	4q12			ENSG00000248494		
HGNC:26524	LOH12CR2	12p13.2	AY037867	503693	ENSG00000205791	11896457, 15284860	NR_024061
HGNC:44169	LOXL1-AS1	15q24.1	AK056885	100287616	ENSG00000261801		NR_040066
HGNC:40346	LPP-AS1	3q28			ENSG00000224563		
HGNC:27952	LPP-AS2	3q27.3	BC042414	339929	ENSG00000270959	12477932	NR_036497
HGNC:51694	LRP1-AS	12q13.3		105751187	ENSG00000259125	25937287	
HGNC:44128	LRP4-AS1	11p11.2		100507401	ENSG00000247675		NR_038909
HGNC:15571	LRRC2-AS1	3p21.3	AJ312775	83598			NR_073385
HGNC:43636	LRRC3-AS1	21q22.3	AA725743, BM703952	100861510	ENSG00000229356		
HGNC:1270	LRRC3DN	21q22.3	AL117578	54083			
HGNC:28619	LRRC75A-AS1	17p11.2	BC027986	125144	ENSG00000175061	12477932	NR_027160
HGNC:40350	LSAMP-AS1	3q13.31		100506708	ENSG00000240922		
HGNC:37824	LSINCT5	5p15.33	GU228577	101234261		21532345, 20214974, 23359273	
HGNC:51845	LUADT1	6q24.3			ENSG00000196634	26291312	
HGNC:48498	LUCAT1	5q14.3	BQ232757, AK091866,	100505994	ENSG00000248323	23672216	
HGNC:51199	LUNAR1	15q26.3				25083870	
HGNC:49761	LURAP1L-AS1	9p23	BQ416907		ENSG00000235448		
HGNC:26593	LY86-AS1	6p25.1	AK091027	285780	ENSG00000216863		NR_026970
HGNC:50560	LYPLAL1-AS1	1q41		643723	ENSG00000228063		
HGNC:52055	LYRM4-AS1	6p25.1		100129461			NR_126015
HGNC:41320	LYST-AS1	1q42.3			ENSG00000229463		
HGNC:43630	LZTS1-AS1	8p21.3			ENSG00000253733		
HGNC:41257	MACC1-AS1	7p21.1	AI125264, BX114725		ENSG00000228598		
HGNC:37193	MACROD2-AS1	20p12.1	BX417920	100379174	ENSG00000235914		
HGNC:16201	MACROD2-IT1	20p12.1	AI026654	140848	ENSG00000227927		
HGNC:51203	MAFA-AS1	8q24.3	BE503758, BF448373, BX114021		ENSG00000254338	25089627	
HGNC:43649	MAFG-AS1	17q25.3	BC009233	92659	ENSG00000265688	12477932	NR_015454
HGNC:51525	MAFTRR	16q23.2		102467146	ENSG00000261390	25621826	NR_104663
HGNC:45093	MAGEA8-AS1	Xq28	CB961226		ENSG00000230899	23223008	
HGNC:40091	MAGI1-AS1	3p14.1		100873983	ENSG00000240175		
HGNC:42436	MAGI1-IT1	3p14.1		151877	ENSG00000272610		
HGNC:40860	MAGI2-AS1	7q21.11			ENSG00000251276		
HGNC:40861	MAGI2-AS2	7q21.11			ENSG00000226978		
HGNC:40862	MAGI2-AS3	7q21.11		100505881	ENSG00000234456		
HGNC:30058	MAGI2-IT1	7q21.11	AF521131	266671	ENSG00000235751	12110681	
HGNC:29665	MALAT1	11q13.1	AF001540	378938	ENSG00000251562	12970751, 22560368	NR_002819
HGNC:48719	MAMDC2-AS1	9q21.12		100507244	ENSG00000204706		

HGNC:48715	MAN1B1-AS1	9q34.3		100289341			NR_027447
HGNC:43732	MANEA-AS1	6q16.1	BC047582	101927288	ENSG00000261366		NR_047502
HGNC:44359	MAP3K14-AS1	17q21.31	AK311429, BC031942	100133991	ENSG00000267278		NR_024434
HGNC:24091	MAPKAPK5-AS1	12q24.12		51275	ENSG00000234608	12477932	NR_015404
HGNC:43738	MAPT-AS1	17q21.31		100128977	ENSG00000264589		NR_024559
HGNC:43741	MAPT-IT1	17q21.31		100130148			NR_024560
HGNC:40865	MAST4-AS1	5q12.3		100506600	ENSG00000229666		
HGNC:41448	MAST4-IT1	5q12.3			ENSG00000249057		
HGNC:40364	MATN1-AS1	1p35.2	AK057725	100129196	ENSG00000186056		NR_034182
HGNC:44584	MBNL1-AS1	3q25.1	AK304857, BM790896	401093			NR_027037
HGNC:40366	MCCC1-AS1	3q27.1			ENSG00000243368		
HGNC:39825	MCF2L-AS1	13q34		100289410	ENSG00000235280		
HGNC:48980	MCHR2-AS1	6q16.2		728012	ENSG00000229315		
HGNC:16417	MCM3AP-AS1	21q22.3	AK001370	114044	ENSG00000215424	12036297	NR_002776
HGNC:51230	MCM8-AS1	20p12.3	AK058185	101929225	ENSG00000278719		NR_110101
HGNC:51655	MCPH1-AS1	8p23.1		100507530	ENSG00000249898		NR_125386
HGNC:39764	MDC1-AS1	6p21.33			ENSG00000224328	25514464	
HGNC:39213	MED4-AS1	13q14.2			ENSG00000229111		
HGNC:48908	MEF2C-AS1	5q14.3	CD702404	101929423	ENSG00000248309	23801869	
HGNC:14575	MEG3	14q32.2	AB032607	55384	ENSG00000214548	10759892, 23790166	NR_002766
HGNC:14574	MEG8	14q32.31	CA396130, AW026953	79104	ENSG00000258399	11481034, 11337479,	NR_024149
HGNC:43874	MEG9	14q32.31	AK094562, AK021527	100507257	ENSG00000223403	19194500	
HGNC:40370	MEIS1-AS2	2p14		100873998	ENSG00000230749		
HGNC:40369	MEIS1-AS3	2p14	DA226783, DA702499	730198	ENSG00000226819		
HGNC:50672	MEOX2-AS1	7p21.2		101927524	ENSG00000229108		
HGNC:17991	MESTIT1	7q32.2	AF482998	317751		12095916	NR_004382
HGNC:40373	MFI2-AS1	3q29		100507057	ENSG00000228109		
HGNC:51356	MGAT3-AS1	22q13.1			ENSG00000227188	25294924	
HGNC:51291	MHRT	14q11.2		104564225		25119045	NR_126491
HGNC:33425	MIAT	22q12.1	AB263414	440823	ENSG00000225783	17066261, 17623775,	NR_003491
HGNC:50731	MIATNB	22q12.1	AK026502	102724827	ENSG00000244625		
HGNC:40932	MID1IP1-AS1	Xp11.4			ENSG00000238123		
HGNC:27669	MIF-AS1	22q11.23		284889	ENSG00000218537	24066864	NR_038911
HGNC:33464	MIMT1	19q13.43	EF110915	100073347		17509818	NR_024059
HGNC:51653	MINCR	8q24.3		100507316	ENSG00000253716	26351698	NR_120682
HGNC:30049	MIR7-3HG	19p13.3	AB058892	284424	ENSG00000176840	11854097	NR_027148
HGNC:27388	MIR9-3HG	15q26.1		254559	ENSG00000255571		NR_133001
HGNC:23564	MIR17HG	13q31.3	AB176707	407975	ENSG00000215417	15126345, 15944707,	NR_027349
HGNC:28219	MIR22HG	17p13.3	BX648321	84981	ENSG00000186594	8619474, 9110174, 24	NR_028502
HGNC:37187	MIR31HG	9p21.3	AK124391	554202	ENSG00000171889	15364902, 22289355,	NR_027054
HGNC:51913	MIR34AHG	1p36.22	EF592573	106614088		17540599	NR_132742
HGNC:1274	MIR99AHG	21q11-q21.1	AF486622	388815	ENSG00000215386	12036298, 25027842	NR_027790
HGNC:39522	MIR100HG	11q24.1	AK091713	399959		19531736, 21031016,	NR_024430
HGNC:48723	MIR124-2HG	8q12.3		100130155	ENSG00000254377		NR_109792
HGNC:49594	MIR133A1HG	18q11.2	BX648566	102723167	ENSG00000265142		NR_029662
HGNC:42871	MIR137HG	1p21.3	AK094607	400765	ENSG00000225206		NR_046105
HGNC:35460	MIR155HG	21q21.3	AF402776	114614	ENSG00000234883	21296997, 23246696	NR_001458
HGNC:48659	MIR181A1HG	1q32.1		100131234	ENSG00000229989		NR_040073
HGNC:37188	MIR181A2HG	9q33.3		100379345	ENSG00000224020	15364906	NR_038975
HGNC:51945	MIR193BHG	16p13.12		100129781	ENSG00000262454		NR_132983
HGNC:51946	MIR194-2HG	11q13.1		105369343	ENSG00000229719		
HGNC:49402	MIR202HG	10q26.3	DB447466	101927671	ENSG00000166917		NR_108078
HGNC:43562	MIR205HG	1q32.2		642587	ENSG00000230937		
HGNC:39524	MIR210HG	11p15.5		100506211	ENSG00000247095	26400545	NR_038262

HGNC:50537	MIR217HG	2p16.1	DA732292		ENSG00000226702		
HGNC:49555	MIR222HG	Xp11.3			ENSG00000270069	23697773	
HGNC:50346	MIR325HG	Xq13.3-q21.1	AK311342	101928469			NR_110400
HGNC:20136	MIR381HG	14q32.31	AA861571	378881	ENSG00000258861		NR_104192
HGNC:39523	MIR497HG	17p13.1		100506755	ENSG00000267532		NR_038310
HGNC:28258	MIR503HG	Xq26.3		84848	ENSG00000223749	24463510	NR_024607
HGNC:52006	MIR548XHG	21q21.1		101927797	ENSG00000224141		NR_109925
HGNC:23642	MIR600HG	9q34.11	BC015678	81571	ENSG00000236901	12477932	NR_026677
HGNC:27659	MIR646HG	20q13.33		284757	ENSG00000228340		NR_046099
HGNC:27662	MIR663AHG	20p11.1		284801	ENSG00000227195	24932682	NR_040095
HGNC:49204	MIR670HG	11p11.2		100507261	ENSG00000235661		
HGNC:51386	MIR762HG	16p11.2		101928736	ENSG00000260083		NR_110940
HGNC:44332	MIR924HG	18q12.2-q12.	AK090603, BG220862,	647946			NR_024391
HGNC:51555	MIR2052HG	8q21.11-q21.13		441355	ENSG00000254349		NR_033830
HGNC:51999	MIR2117HG	17q21.31	DA448302	106660605	ENSG00000267151		
HGNC:51944	MIR3142HG	5q33.3			ENSG00000253522		
HGNC:50676	MIR3663HG	10q25.3	BC039338	101927704	ENSG00000234474		NR_121650
HGNC:52001	MIR3681HG	2p24.3		100506457	ENSG00000224184		NR_110196
HGNC:52002	MIR3945HG	4q35.1		731424	ENSG00000251230		NR_037867
HGNC:51104	MIR3976HG	18p11.31		645355	ENSG00000261738		
HGNC:27861	MIR4290HG	9q22.2		286370	ENSG00000227555		NR_038882
HGNC:52003	MIR4300HG	11q14.1		101928989	ENSG00000245832		NR_120571
HGNC:52004	MIR4307HG	14q12		101927081	ENSG00000257612		NR_110041
HGNC:52005	MIR4432HG	2p16.1		106660609	ENSG00000223929		NR_132991
HGNC:35163	MIR4435-2HG	2q13	BX647931	541471	ENSG00000172965	19531736, 25888808	NR_015395
HGNC:49008	MIR4458HG	5p15.31		100505738	ENSG00000247516		NR_039989
HGNC:42773	MIR4500HG	13q31.2		642345	ENSG00000228824		NR_033829
HGNC:27448	MIR4697HG	11q25		283174			NR_024344
HGNC:52007	MIR5689HG	6p24.3		106660610	ENSG00000229401		NR_132993
HGNC:49838	MIR7515HG	2p25.2	BX119852, DB061664	102800314	ENSG00000236172		NR_110497
HGNC:37189	MIRLET7BHG	22q13.31		400931	ENSG00000197182	15364908, 24381249	NR_027033
HGNC:37186	MIRLET7DHG	9q22.32		158257	ENSG00000230262	15364901	NR_046163
HGNC:40106	MIS18A-AS1	21q22.11	BG209386		ENSG00000227256		
HGNC:40374	MKLN1-AS	7q32.3	AK054623, DB039005	100506881	ENSG00000236753		
HGNC:44129	MKNK1-AS1	1p33	AK097731	100507423			NR_038403
HGNC:12910	MKRN3-AS1	15q11-q13		10108		10196367, 9237260	
HGNC:50686	MKX-AS1	10p12.1	BU195543	101929202	ENSG00000230500		
HGNC:40963	MLIP-AS1	6p12.1			ENSG00000235050		
HGNC:41461	MLIP-IT1	6p12.1			ENSG00000236996		
HGNC:27935	MLK7-AS1	2q31.1	BC040565	339751	ENSG00000238133	12477932	NR_033882
HGNC:21236	MLLT4-AS1	6q27		653483	ENSG00000198221	10382971	NR_027906
HGNC:40376	MME-AS1	3q25.2			ENSG00000240666		
HGNC:44421	MMP24-AS1	20q11.22	BG546906, DA451679		ENSG00000126005		
HGNC:51372	MMP25-AS1	16p13.3	BC026114, AK124978	100507419	ENSG00000261971		NR_123723
HGNC:48954	MNX1-AS1	7q36.3		645249	ENSG00000243479		
HGNC:40278	MNX1-AS2	7q36.3	AI761375	105375606	ENSG00000235029		
HGNC:40377	MORC1-AS1	3q13.13		100506506	ENSG00000239314		
HGNC:26662	MORC2-AS1	22q12.2	AK093120	150291	ENSG00000235989		NR_026920
HGNC:27991	MORF4L2-AS1	Xq22.2	BC043382	340544	ENSG00000231154	12477932	NR_038978
HGNC:41263	MPRIP-AS1	17p11.2	BG548937	100874529	ENSG00000225442		
HGNC:51140	MRGPRF-AS1	11q13.3		101928200	ENSG00000256508		
HGNC:26691	MRGPRG-AS1	11p15.4	AK097749	283303	ENSG00000236301		NR_027138
HGNC:42812	MRPL23-AS1	11p15.5		100133545	ENSG00000226416		NR_024471
HGNC:43434	MRVI1-AS1	11p15.4		100129827	ENSG00000177112		NR_034093

HGNC:48724	MSC-AS1	8q13.3		100132891	ENSG00000235531		NR_033652
HGNC:50279	MT-LIPCAR	mitochondria	JA760602	103504742		24663402	
HGNC:40242	MTOR-AS1	1p36.22			ENSG00000225602		
HGNC:40924	MTUS2-AS1	13q12.3			ENSG00000179141		
HGNC:40923	MTUS2-AS2	13q12.3			ENSG00000236758		
HGNC:37457	MYB-AS1	6q22-q23			ENSG00000236703		
HGNC:41023	MYCBP2-AS1	13q22.3			ENSG00000236051		
HGNC:41024	MYCBP2-AS2	13q22.3			ENSG00000229521		
HGNC:16911	MYCNOS	2p24.1	S49953	10408	ENSG00000233718	1419902, 12880964, 19615087, 2439	
HGNC:32684	MYCNUT	2p24.3	W87755		ENSG00000223850	24906397	
HGNC:50609	MYHAS	17p13.1	AK097500	100128560	ENSG00000272975		
HGNC:42440	MYLK-AS1	3q21.1		100506826	ENSG00000239523		
HGNC:40387	MYLK-AS2	3q21.1			ENSG00000250174		
HGNC:39913	MYO16-AS1	13q33.3			ENSG00000236242		
HGNC:39914	MYO16-AS2	13q33.3			ENSG00000229938		
HGNC:49274	MYT1L-AS1	2p25.3		730811	ENSG00000225619		
HGNC:51271	MZF1-AS1	19q13.43	BC024736	100131691	ENSG00000267858		NR_027334
HGNC:25038	N4BP2L2-IT2	13q13.1	U50531	116828		8812419	NR_026928
HGNC:41016	NAALADL2-AS1	3q26.3			ENSG00000225552		
HGNC:41015	NAALADL2-AS2	3q26.3			ENSG00000226779		
HGNC:41014	NAALADL2-AS3	3q26.3			ENSG00000230292		
HGNC:41143	NADK2-AS1	5p13.2	BC033402		ENSG00000245711		
HGNC:44184	NAGPA-AS1	16p13.3	AK055222	100507589			NR_038913
HGNC:42743	NALCN-AS1	13q33.1		100885778	ENSG00000233009		
HGNC:51192	NALT1	9q34.3		101928483	ENSG00000237886	26330272	NR_121577
HGNC:42408	NAMA	9q22.33	DQ673922	100996569		17415708	
HGNC:44118	NAPA-AS1	19q13.32-q13	AK057858	100505681			NR_038452
HGNC:43651	NARF-IT1	17q25.3		100506001			
HGNC:40744	NAV2-AS1	11p15.1			ENSG00000254894		
HGNC:40743	NAV2-AS2	11p15.1			ENSG00000254453		
HGNC:40742	NAV2-AS3	11p15.1			ENSG00000254542		
HGNC:40741	NAV2-AS4	11p15.1		399876	ENSG00000254622		
HGNC:40740	NAV2-AS5	11p15.1		100874012	ENSG00000255043		
HGNC:41416	NAV2-IT1	11p15.1			ENSG00000255270		
HGNC:49075	NBAT1	6p22.3		729177	ENSG00000260455	25859549, 26378045	NR_034143
HGNC:20691	NBR2	17q21	U88573	10230	ENSG00000198496	9215675, 15777733	NR_003108
HGNC:48675	NCAM1-AS1	11q23.2		100288346	ENSG00000227487		
HGNC:40392	NCBP2-AS1	3q29			ENSG00000225578		
HGNC:25121	NCBP2-AS2	3q29	BC007882	152217	ENSG00000270170	12477932	NM_138487
HGNC:49645	NCK1-AS1	3q22.3		101927597	ENSG00000239213		
HGNC:41497	NCKAP5-IT1	2q21.2			ENSG00000232474		
HGNC:40954	NCOA7-AS1	6q22.32	AW444508		ENSG00000232131	23801869	
HGNC:37153	NCRUPAR	5q13.3		100302746		12084570	NR_028375
HGNC:40844	NDFIP2-AS1	13q31.1			ENSG00000232132		
HGNC:40395	NDP-AS1	Xp11.3			ENSG00000236276		
HGNC:45273	NDUFA6-AS1	22q13.2	BC039542	100132273	ENSG00000237037		NR_034118
HGNC:40396	NDUFB2-AS1	7q34	AK129584	100134713	ENSG00000240889		NR_024454
HGNC:50826	NDUFV2-AS1	18p11.22		101927275	ENSG00000266053		
HGNC:30815	NEAT1	11q13.1	AF080092	283131	ENSG00000245532	9253601, 9858482, 12	NR_028272
HGNC:44899	NEBL-AS1	10p12.31		100128511	ENSG00000231920		NR_046283
HGNC:41432	NEGR1-IT1	1p31.1	BC042082		ENSG00000228853		
HGNC:51220	NEURL1-AS1	10q24.33	AA399019	102724341	ENSG00000235470		NR_120675
HGNC:31983	NEXN-AS1	1p31.1	AK075118	374987	ENSG00000235927		NM_199343
HGNC:40402	NFIA-AS1	1p31.3		645030	ENSG00000237853	25265644	NR_104180

HGNC:40401	NFIA-AS2	1p31.3		100996570	ENSG00000237928		
HGNC:49451	NFYC-AS1	1p34.2		100130557	ENSG00000272145		
HGNC:40403	NHS-AS1	Xp22.13			ENSG00000230020		
HGNC:27385	NIFK-AS1	2q14.3		254128	ENSG00000236859	12477932	
HGNC:51293	NIPBL-AS1	5p13.2		646719		24007600	NR_046262
HGNC:51599	NKILA	20q13.31	AK056098	105416157	ENSG00000278709	25759022, 26069731	NR_131157
HGNC:40585	NKX2-1-AS1	14q13.3		100506237	ENSG00000253563		
HGNC:37154	NKX2-2-AS1	20p11.22			ENSG00000258197	18538132	
HGNC:40676	NLGN1-AS1	3q26.31			ENSG00000228213		
HGNC:38793	NLGN4Y-AS1	Yq11.221		100874056	ENSG00000228787		
HGNC:49005	NNT-AS1	5p12		100652772	ENSG00000248092		
HGNC:20205	NOP14-AS1	4p16.3	AB000465	317648	ENSG00000249673	9734812	NR_015453
HGNC:44311	NORAD	20q11.23	AI619767, AK090641, I	647979	ENSG00000260032		NR_027451
HGNC:19827	NOVA1-AS1	14q12	AI278049	387977	ENSG00000257842		
HGNC:24129	NPHP3-AS1	3q22.1	BC048124	348808	ENSG00000248724	12477932	NR_002811
HGNC:37635	NPPA-AS1	1p36.21			ENSG00000242349	19671135	
HGNC:22128	NPSR1-AS1	7p14.3	BC031961	404744	ENSG00000197085	15073379	NR_015356
HGNC:45091	NPTN-IT1	15q24.1	AK055007	101241892		23395002	NR_103844
HGNC:48622	NR2F1-AS1	5q15	BC010610, DA718791	441094	ENSG00000237187	23583100	NR_021490
HGNC:44222	NR2F2-AS1	15q26.2	AK000872	644192	ENSG00000247809		XR_109225
HGNC:48588	NRAV	12q24.31	AK026783, BC065744	100506668	ENSG00000248008	25525793	NR_038854
HGNC:40780	NREP-AS1	5q22.1			ENSG00000250095		
HGNC:43633	NRG1-IT1	8p12	BC037250		ENSG00000253974		
HGNC:43635	NRG1-IT3	8p12			ENSG00000254049		
HGNC:31429	NRG3-AS1	10q23.1	CF272718, AA383013, .	101929590	ENSG00000225738		
HGNC:51269	NRIR	2p25.2	BQ437827		ENSG00000225964	25122750	
HGNC:37079	NRON	9q33.3		641373	ENSG00000253079	16141075	NR_045006
HGNC:51222	NRSN2-AS1	20p13	BG249690	100507459	ENSG00000225377		NR_109990
HGNC:32293	NTM-AS1	11q25	AK127362	101929637	ENSG00000224795		NR_126159
HGNC:41437	NTM-IT	11q25	AA398869		ENSG00000238262		
HGNC:27532	NTRK3-AS1	15q25.3	AK097486	283738	ENSG00000260305		NR_038229
HGNC:40419	NUCB1-AS1	19q13.33			ENSG00000235191		
HGNC:50502	NUP50-AS1	22q13.31		100506714	ENSG00000226328		
HGNC:45161	NUTM2A-AS1	10q23.2	AK311174, AK309777	728190	ENSG00000223482		NR_024397
HGNC:51204	NUTM2B-AS1	10q22.3		101060691	ENSG00000225484		NR_120613
HGNC:40751	OCIAD1-AS1	4p11	AI018597, BG200457		ENSG00000248256		
HGNC:49461	ODF2-AS1	9q34.11	AW303426		ENSG00000225951		
HGNC:40724	OGFR-AS1	20q13.33	BQ187924, CA412981		ENSG00000229873		
HGNC:43563	OIP5-AS1	15q15.1		729082	ENSG00000247556	22196729	NR_026757
HGNC:28060	OLMALINC	10q24.31	BC045787	90271	ENSG00000235823	12477932, 23040067	NR_026762
HGNC:39192	OOEP-AS1	6q13			ENSG00000231332		
HGNC:40421	OPA1-AS1	3q29		100873941	ENSG00000224855		
HGNC:41366	OPCML-IT1	11q25			ENSG00000254896		
HGNC:41365	OPCML-IT2	11q25			ENSG00000255371		
HGNC:49168	OR2A1-AS1	7q35	BC040701	101928492	ENSG00000244479		
HGNC:40767	OSBPL10-AS1	3p23			ENSG00000232490		
HGNC:48585	OSER1-AS1	20q13.12		100505783	ENSG00000223891		
HGNC:41009	OSGEPL1-AS1	2q32.2	BX474356, DB142079		ENSG00000253559		
HGNC:50296	OSMR-AS1	5p13.1		101929768	ENSG00000249740		
HGNC:43666	OSTM1-AS1	6q21	BC031282	100287366	ENSG00000225174		
HGNC:41250	OSTN-AS1	3q28			ENSG00000233308		
HGNC:50466	OTUD6B-AS1	8q21.3		100506365	ENSG00000253738		
HGNC:43906	OTX2-AS1	14q22.3	BC041486	100309464	ENSG00000248550	15703187	NR_029385
HGNC:49422	OVAAL	1q25.3	BC036830	148756	ENSG00000236719	24265805	

HGNC:44484	OVCH1-AS1	12p11.22		101055625	ENSG00000257599		
HGNC:49319	OVOL1-AS1	11q13.1	AI458914	101927828	ENSG00000255120		
HGNC:40423	OXCT1-AS1	5p13.1	DB470733, DB476656		ENSG00000248668		
HGNC:40886	P3H2-AS1	3q28			ENSG00000225764		
HGNC:43560	P4HA2-AS1	5q31.1	BG772701		ENSG00000237714		
HGNC:50345	PABPC1L2B-AS	Xq13.2		101928345	ENSG00000226725		
HGNC:31845	PABPC5-AS1	Xq21.31		102724167	ENSG00000234161		
HGNC:50552	PACERR	1q31.1	DA380694, BU733503		ENSG00000273129	24843008	
HGNC:27772	PACRG-AS1	6q26		285796	ENSG00000281692		NR_028390
HGNC:40871	PACRG-AS2	6q26		101929239	ENSG00000225437		NR_110871
HGNC:52053	PACRG-AS3	6q26		729658	ENSG00000225683		NR_122120
HGNC:39932	PAN3-AS1	13q12.2	BC045730	100288730			NR_029383
HGNC:52282	PANCR	4q25		105377363	ENSG00000250103	26783232	
HGNC:44048	PANDAR	6p21.2	JF803844	101154753		21642992	NR_109836
HGNC:35152	PAPPA-AS1	9q33.2		493913	ENSG00000256040	15656990	XR_041634
HGNC:35160	PAPPA-AS2	9q33.1	AK092105		ENSG00000226604	19531736	
HGNC:50861	PAQR9-AS1	3q23	DB456045	101927832	ENSG00000241570		
HGNC:44964	PARD3-AS1	10p11.21	AI792816, BX089194	100505601	ENSG00000226386		
HGNC:44109	PARD6G-AS1	18q23	AK056304	100130522			NR_028339
HGNC:17263	PART1	5q12.1	AF163475	25859	ENSG00000152931	10706094, 23726844	NR_024617
HGNC:50886	PARTICL	2p11.2	BC009563	100630918		25900080	NR_038942
HGNC:49670	PAUPAR	11p13	KJ434992	103157000		24488179	
HGNC:49271	PAX8-AS1	2q13	AK130275	654433	ENSG00000189223		
HGNC:39603	PAXBP1-AS1	21q22.11	BC038361	100506215	ENSG00000238197		
HGNC:27328	PAXIP1-AS1	7q36.2	BI438440	202781		12477932	NR_028090
HGNC:48958	PAXIP1-AS2	7q36.2		100132707	ENSG00000214106		NR_024476
HGNC:8637	PCA3	9q21.2	AF103907	50652	ENSG00000225937	10606244, 23130941,	NR_015342
HGNC:43022	PCAT1	8q24.21	HQ605084	100750225	ENSG00000253438	21804560, 23640607	NR_045262
HGNC:45089	PCAT2	8q24.21	BU852210	103164619	ENSG00000253264		
HGNC:24853	PCAT4	4q21.21	AF388200	118425	ENSG00000251321	11536302, 15299187,	NR_026555
HGNC:48665	PCAT5	10p11.21	KF154780	102578074	ENSG00000280719	26282172	NR_110138
HGNC:43714	PCAT6	1q32.1	BI962506, AI806248	100506696	ENSG00000228288	20887892, 23728290	
HGNC:48824	PCAT7	9q22.32		101928099	ENSG00000231806	23728290	NR_121566
HGNC:48977	PCAT14	22q11.23	HQ605085	101978785		21804560	NR_109832
HGNC:49211	PCAT18	18q11.2		728606	ENSG00000265369	24519926	
HGNC:49593	PCAT19	19q13.2	BC070102	100505495	ENSG00000267107	24497837	NR_040109
HGNC:50895	PCAT29	15q23		104472713	ENSG00000259641	25030374	NR_126437
HGNC:42948	PCBP1-AS1	2p13.3		400960	ENSG00000179818		NR_033872
HGNC:49150	PCBP2-OT1	12q13.13	KF041153	102157401		21187392	
HGNC:33548	PCBP3-OT1	21q22.3	AK126016			17525332	
HGNC:39901	PCCA-AS1	13q32.3			ENSG00000234650		
HGNC:39897	PCDH9-AS1	13q21.32			ENSG00000234527		
HGNC:39896	PCDH9-AS2	13q21.32			ENSG00000228842		
HGNC:40427	PCDH9-AS3	13q21.32			ENSG00000225263		
HGNC:40428	PCDH9-AS4	13q21.32			ENSG00000233840		
HGNC:44166	PCED1B-AS1	12q13.11	AK123977	100233209	ENSG00000247774		NR_026544
HGNC:52263	PCF11-AS1	11q14.1	AK092827		ENSG00000269939	26421281	
HGNC:30145	PCGEM1	2q32.3	AF223389	64002	ENSG00000227418	11050243, 16569192,	NR_002769
HGNC:37184	PCNA-AS1	20p12.3		100302739		10488129, 24704293	
HGNC:40430	PCOLCE-AS1	7q22.1	AK097705	100129845	ENSG00000224729		NR_038910
HGNC:51448	PCSK6-AS1	15q26.3	BG505308		ENSG00000259764		
HGNC:40431	PCYT1B-AS1	Xp22.11			ENSG00000236836		
HGNC:27425	PDCD4-AS1	10q25.2		282997	ENSG00000203497		NR_026932
HGNC:43698	PDX1-AS1	13q12.2	BX383058, CR593175		ENSG00000247381		

HGNC:40814	PDZRN3-AS1	3p13		101927249	ENSG00000239677		
HGNC:35127	PEG3-AS1	19q13.43		100169890		18166281, 15950772,	NR_023847
HGNC:19969	PEG13	8q24.3	AK307073		ENSG00000282164	24402520, 24667089, 24980697	
HGNC:41251	PEX5L-AS1	3q26.33			ENSG00000243799		
HGNC:41252	PEX5L-AS2	3q26.33		101928790	ENSG00000244302		NR_110059
HGNC:44181	PGM5-AS1	9q21.11	AY343894	572558	ENSG00000224958		NR_015423
HGNC:51194	PGM5P3-AS1	9p24.3		101929127	ENSG00000277631		NR_121188
HGNC:51195	PGM5P4-AS1	2q14.1		103344932	ENSG00000231943		NR_121185
HGNC:40943	PHACTR2-AS1	6q24.2		285740	ENSG00000235740		NR_027114
HGNC:40445	PHEX-AS1	Xp22.11			ENSG00000224204		
HGNC:40446	PHKA1-AS1	Xq13.2		100506671	ENSG00000231944		
HGNC:44110	PHKA2-AS1	Xp22.13	BC073810	100132163	ENSG00000237836		NR_029379
HGNC:19725	PICSAR	21q22.3	BC027456	378825	ENSG00000275874	16826516, 27049681	
HGNC:32346	PIK3CD-AS1	1p36.22	BC016063	644997	ENSG00000179840		NR_027045
HGNC:51334	PIK3CD-AS2	1p36.22	AL523099, BI763597, B	101929074	ENSG00000231789		
HGNC:41072	PIK3IP1-AS1	22q12.2	BF439736	101929760	ENSG00000228839		NR_110542
HGNC:38872	PINK1-AS	1p36.12	AK090466		ENSG00000117242	17362513	
HGNC:16671	PISRT1	3q23	AW268472	140464			NR_027070
HGNC:44116	PITPNA-AS1	17p13.3	HY016432, DB053306,	100306951	ENSG00000236618		NR_028514
HGNC:44675	PITRM1-AS1	10p15.2	BC050332	100507034	ENSG00000237399		NR_038284
HGNC:51659	PKIA-AS1	8q21.13	BC036404	101927003	ENSG00000254266		NR_125389
HGNC:50597	PKN2-AS1	1p22.2		101927891	ENSG00000237505		
HGNC:50690	PKNOX2-AS1	11q24.2	BP872841		ENSG00000254880		
HGNC:51585	PLA2G4C-AS1	19q13.33	AA318256, BU736556, EL953961		ENSG00000269420		
HGNC:51419	PLA2G4E-AS1	15q15.1	BC043275	101928388	ENSG00000246740		NR_120334
HGNC:51143	PLBD1-AS1	12p13.1		101928290	ENSG00000256751		
HGNC:41414	PLCB1-IT1	20p12.3			ENSG00000225479		
HGNC:43662	PLCB2-AS1	15q15.1			ENSG00000259307		
HGNC:45193	PLCE1-AS1	10q23.33		100128054	ENSG00000268894		NR_033969
HGNC:51206	PLCE1-AS2	10q23.33	AK098548	101927049	ENSG00000232913		NR_120615
HGNC:40450	PLCG1-AS1	20q12		101927117	ENSG00000226648		
HGNC:41231	PLCH1-AS1	3q25.31			ENSG00000239508		
HGNC:41232	PLCH1-AS2	3q25.31			ENSG00000242925		
HGNC:40449	PLCL2-AS1	3p24.3			ENSG00000226441		
HGNC:41145	PLCXD2-AS1	3q13.2			ENSG00000240766		
HGNC:40451	PLS1-AS1	3q23			ENSG00000239641		
HGNC:50343	PLS3-AS1	Xq23		101927352	ENSG00000271826		
HGNC:40907	PLSCR5-AS1	3q24			ENSG00000241457		
HGNC:49459	POT1-AS1	7q31.33	BX648695		ENSG00000224897		
HGNC:40058	POTEH-AS1	22q11.1	AY338953		ENSG00000236666		
HGNC:40979	POU6F2-AS1	7p14.1	AI807878, BX113391		ENSG00000224122		
HGNC:21887	POU6F2-AS2	7p14.1	AK023033		ENSG00000233854		
HGNC:40463	PPEF1-AS1	Xp22.13			ENSG00000237221		
HGNC:48717	PPP1R26-AS1	9q34.3		100506599	ENSG00000225361		
HGNC:42984	PPP2R2B-IT1	5q32			ENSG00000249553		
HGNC:50750	PPP3CB-AS1	10q22.2		101929145	ENSG00000221817		
HGNC:51112	PPP4R1-AS1	18p11.22		101927323	ENSG00000263627		
HGNC:48587	PRC1-AS1	15q26.1		100507118	ENSG00000258725		
HGNC:40916	PRICKLE2-AS1	3p14.1		100652759	ENSG00000241572		
HGNC:40917	PRICKLE2-AS2	3p14.1			ENSG00000241101		
HGNC:40918	PRICKLE2-AS3	3p14.1			ENSG00000226017		
HGNC:34235	PRINS	10p12.1	AK022045	100169750		15855153, 20377629,	NR_023388
HGNC:40468	PRKAG2-AS1	7q36.1	BC131560	100505483	ENSG00000239911		NR_038926
HGNC:40471	PRKAR2A-AS1	3p21.31		100506637	ENSG00000224424		

HGNC:51347	PRKCA-AS1	17q24.2	BC033554	101928001	ENSG00000264630		NR_110822
HGNC:44689	PRKCQ-AS1	10p14		439949	ENSG00000237943	26421281	NR_036502
HGNC:45029	PRKG1-AS1	10q21.1	BC039504	100506939	ENSG00000236671		NR_038277
HGNC:40479	PRKX-AS1	Xp22.33			ENSG00000236188		
HGNC:40533	PRMT5-AS1	14q11.2	AK093277	100505758	ENSG00000237054		
HGNC:48942	PRNCR1	8q24.21	AB458446	101867536		20874843, 23945587,	NR_109833
HGNC:27343	PROSER2-AS1	10p14		219731	ENSG00000225778		NR_038222
HGNC:43656	PROX1-AS1	1q32.3	AK092251	100505832	ENSG00000230461		NR_037850
HGNC:27961	PRR7-AS1	5q35.3	BC041422	340037	ENSG00000246334	12477932	NR_038915
HGNC:51822	PRR29-AS1	17q23.3	AK094767	400612	ENSG00000264954		NR_126169
HGNC:50499	PRR34-AS1	22q13.31		150381	ENSG00000241990		
HGNC:41151	PRRT3-AS1	3p25.3			ENSG00000230082		
HGNC:50684	PRRX2-AS1	9q34.11		101929437	ENSG00000236024		
HGNC:26445	PSMA3-AS1	14q23.1		379025	ENSG00000257621	25392693	NR_029434
HGNC:39758	PSMB8-AS1	6p21.32		100507463	ENSG00000204261		NR_037173
HGNC:27367	PSMD5-AS1	9q33.2		253039	ENSG00000226752	12477932	
HGNC:44125	PSMD6-AS2	3p14.1	AK023371	100507062	ENSG00000239653		NR_038286
HGNC:22230	PSMG3-AS1	7p22.3		114796	ENSG00000230487		NR_021487
HGNC:17203	PSORS1C3	6p21.33		100130889	ENSG00000204528		NR_026816
HGNC:37703	PTCHD1-AS	Xp22.11	DA355362, BX115199	100873065	ENSG00000233067		NR_073010
HGNC:37127	PTCSC1	8q24	AK023948	100302522		19147577	
HGNC:44086	PTCSC2	9q22.33	HG502283, BE073442	101928337	ENSG00000236130	23408906, 25303483	
HGNC:43959	PTCSC3	14q13.3	JN689234, CB987890	100886964	ENSG00000259104	22586128, 26274343	NR_049735
HGNC:48773	PTENP1-AS	9p13.3	BX374997			23435381	
HGNC:48711	PTGES2-AS1	9q34.11	BC141829, BC151210	389791			
HGNC:44174	PTOV1-AS1	19q13.33	BC043359	100506033			NR_040037
HGNC:51284	PTOV1-AS2	19q13.33		101928378	ENSG00000269352		NR_110730
HGNC:49753	PTPRD-AS1	9p24.1	AK094342	101929407	ENSG00000225706		
HGNC:49754	PTPRD-AS2	9p23	BC045688	101929428	ENSG00000226717		
HGNC:44638	PTPRG-AS1	3p14.2	AK308660, BC041886	100506994	ENSG00000241472		NR_038281
HGNC:50634	PTPRJ-AS1	11p11.2	AI365468		ENSG00000254879	23840844	
HGNC:40813	PVRL3-AS1	3q13.13		100506555	ENSG00000242242		
HGNC:9709	PVT1	8q24	M31519	5820	ENSG00000249859	17503467, 23801869	NR_003367
HGNC:30089	PWAR1	15q11.2		145624		7987392, 9477342	
HGNC:29998	PWAR4	15q11.2		347745		11106375, 7987392	
HGNC:30090	PWAR5	15q11.2		8123		7987392, 9477342, 23726844	
HGNC:49129	PWAR6	15q11.2	BC043194, AK096584	100506965	ENSG00000257151		
HGNC:30001	PWARSN	15q11.2		347746		12210318, 8858349	NR_022011
HGNC:33235	PWRN1	15q11.2	AC139362, AC139147,	791114	ENSG00000259905	17337158	NR_026646
HGNC:33236	PWRN2	15q11.2	AC087474	791115		17337158	NR_026647
HGNC:49131	PWRN3	15q11.2	AA412395, DB060889	101928840	ENSG00000260760		
HGNC:49130	PWRN4	15q11.2	CK820921, BI712798		ENSG00000260232		
HGNC:44123	PXN-AS1	12q24.23		100506649	ENSG00000255857		NR_038924
HGNC:45036	PYCARD-AS1	16p11.2		100652740	ENSG00000261359		NR_102400
HGNC:49278	RAB6C-AS1	2q21.1		100131320	ENSG00000225449		
HGNC:44178	RAB11B-AS1	19p13.2	BC014506	100507567			NR_038237
HGNC:48672	RAB30-AS1	11q14.1		100506233	ENSG00000246067		
HGNC:41471	RABGAP1L-IT1	1q25.1			ENSG00000223525		
HGNC:32158	RAD21-AS1	8q24.11	BC031280	644660	ENSG00000253327		XR_040079
HGNC:48621	RAD51-AS1	15q15.1	AK125393	100505648	ENSG00000245849	26230935	NR_040058
HGNC:48994	RAET1E-AS1	6q25.1		100652739	ENSG00000223701		
HGNC:40496	RAI1-AS1	17p11.2	BX366317, BX401587		ENSG00000237328		
HGNC:50743	RALY-AS1	20q11.22		101926888	ENSG00000228265		
HGNC:44358	RAMP2-AS1	17q21.31	BC018638, BC041021	100190938	ENSG00000197291		NR_024461

HGNC:40957	RAP2C-AS1	Xq26.2		101928578	ENSG00000232160		
HGNC:28081	RAPGEF4-AS1	2q31.1	AL157450	91149	ENSG00000228016		NR_026995
HGNC:49577	RARA-AS1	17q21.2	BC038432	101929693	ENSG00000265666		
HGNC:41376	RASA2-IT1	3q23			ENSG00000250170		
HGNC:39938	RASA3-IT1	13q34			ENSG00000232487		
HGNC:44170	RASAL2-AS1	1q25.2	BC027927	100302401	ENSG00000224687		NR_027982
HGNC:40499	RASGRF2-AS1	5q14.1	BM682385	102524628	ENSG00000251450		
HGNC:49091	RASSF1-AS1	3p21.3	KC330992	102060282		23990798	NR_109831
HGNC:48637	RASSF8-AS1	12p12.1		100506451	ENSG00000246695		
HGNC:33770	RBAKDN	7p22.1	BC031661	389458			NR_015343
HGNC:48591	RBFADN	18q23		100506070			NR_103445
HGNC:48871	RBM5-AS1	3p21.31	EF470865	100775107		10949932, 19559772	NR_045388
HGNC:28818	RBM12B-AS1	8q22.1	AF116672	55472	ENSG00000279331		NR_027259
HGNC:39805	RBM26-AS1	13q31.1	BC034812	100505538	ENSG00000227354		
HGNC:39987	RBMS3-AS1	3p24.1			ENSG00000235593		
HGNC:39988	RBMS3-AS2	3p24.1			ENSG00000203506		
HGNC:39989	RBMS3-AS3	3p24.1			ENSG00000235904		
HGNC:48721	RBPMS-AS1	8p12		100128750	ENSG00000254109		
HGNC:41496	RC3H1-IT1	1q25.1			ENSG00000236535		
HGNC:51658	RDH10-AS1	8q21.11		101926926	ENSG00000250295		NR_125388
HGNC:39990	RERG-AS1	12p12.3	BF508473, AW197661		ENSG00000255660		
HGNC:39991	RERG-IT1	12p12.3			ENSG00000256650		
HGNC:41377	REV3L-IT1	6q21			ENSG00000229276		
HGNC:9978	RFPL1S	22q12	AJ010230	10740	ENSG00000225465	10508838	NR_002727
HGNC:51197	RFX3-AS1	9p24.2	BG720696	101929302	ENSG00000232104		NR_121586
HGNC:48666	RGMB-AS1	5q15		503569	ENSG00000246763	26055877	NR_033932
HGNC:49273	RGPD4-AS1	2q12.3		729121	ENSG00000230651		
HGNC:41308	RHOA-IT1	3p21.31			ENSG00000235908		
HGNC:51582	RHOXF1-AS1	Xq24	AK123976	101928969	ENSG00000258545		
HGNC:28457	RHPN1-AS1	8q24		78998	ENSG00000254389	12477932	NR_026785
HGNC:41150	RMDN2-AS1	2p22.2	DB142860		ENSG00000235848		
HGNC:29893	RMST	12q23.1	AK056164	196475	ENSG00000255794	12082533, 20062813	NR_024037
HGNC:49289	RNASEH1-AS1	2p25.3		100506054	ENSG00000234171		
HGNC:39967	RNASEH2B-AS1	13q14.3			ENSG00000233672		
HGNC:48940	RNF139-AS1	8q24.13	AA905116	101927612	ENSG00000245149		NR_108047
HGNC:30963	RNF144A-AS1	2p25.2		386597	ENSG00000228203		NR_033997
HGNC:44127	RNF157-AS1	17q25.1	AK093500	100507218			NR_040017
HGNC:41161	RNF185-AS1	22q12.2	BC069286		ENSG00000254835		
HGNC:41463	RNF216-IT1	7p22.1			ENSG00000237738		
HGNC:50866	RNF217-AS1	6q22.31		7955	ENSG00000236548	9087565	
HGNC:42700	RNF219-AS1	13q31.1		100874222	ENSG00000234377		
HGNC:39984	ROPN1L-AS1	5p15.2		100505845	ENSG00000250600		
HGNC:40508	ROR1-AS1	1p31.3		101927034	ENSG00000223949		
HGNC:51410	RORA-AS1	15q22.2	BC035094	101928784	ENSG00000245534		NR_120339
HGNC:51411	RORA-AS2	15q22.2	DB058680, DB033761	100996876	ENSG00000259482		NR_120318
HGNC:49803	RORB-AS1	9q21.13	BI520565		ENSG00000224825		
HGNC:45238	RPARP-AS1	10q24.32		100505761	ENSG00000269609		NR_038937
HGNC:26749	RPL34-AS1	4q25		285456	ENSG00000234492	24908062	
HGNC:40511	RPS6KA2-AS1	6q27	AA707471, AW290888	100861523	ENSG00000231654	19287968	
HGNC:41378	RPS6KA2-IT1	6q27			ENSG00000232082		
HGNC:40512	RRM1-AS1	11p15.4			ENSG00000255276		
HGNC:50465	RRS1-AS1	8q13.1		100505676	ENSG00000246145		
HGNC:41439	RSF1-IT1	11q14.1			ENSG00000255409		
HGNC:41438	RSF1-IT2	11q14.1			ENSG00000254985		

HGNC:50573	RTCA-AS1	1p21.2		100506007	ENSG00000224616		
HGNC:51344	RUNDC3A-AS1	17q21.31	AK055254, BC051705	101926996	ENSG00000267750		NR_110802
HGNC:16623	RUNX1-IT1	21q22.12	AK024509	80215			NR_026812
HGNC:26680	RUSC1-AS1	1q22	AK093295	284618	ENSG00000225855	14702039	
HGNC:40514	RUVBL1-AS1	3q21.3			ENSG00000239608		
HGNC:39835	SACS-AS1	13q12.12		100506680	ENSG00000229558		
HGNC:49001	SALRNA1	14q23.1				23758631	
HGNC:49003	SALRNA2	15q23				23758631	
HGNC:49002	SALRNA3	15q23				23758631	
HGNC:30937	SAMD12-AS1	8q24.12	BE069098	552860			
HGNC:49644	SAMMSON	3p13		101927152	ENSG00000240405	27008969	NR_110000
HGNC:39599	SAMSN1-AS1	21q11.2			ENSG00000223662		
HGNC:26760	SAP30L-AS1	5q33.2		386627	ENSG00000245275	12477932	
HGNC:39824	SAPCD1-AS1	6p21.33			ENSG00000235663		
HGNC:50687	SATB1-AS1	3p24.3	DR006704		ENSG00000228956		
HGNC:26490	SATB2-AS1	2q33.1		150538	ENSG00000225953		NR_026830
HGNC:27438	SBF2-AS1	11p15.4		283104	ENSG00000246273		NR_036485
HGNC:43718	SCAANT1	3p14.1	BU569004	100861563		21689595	
HGNC:50544	SCAMP1-AS1	5q14.1		728769	ENSG00000245556		
HGNC:39895	SCEL-AS1	13q22.3			ENSG00000224347		
HGNC:48603	SCHLAP1	2q31.3	JX117418	101669767		24076601, 24165726	NR_104323
HGNC:50601	SCOC-AS1	4q31.1		100129858	ENSG00000196951		
HGNC:44314	SDCBP2-AS1	20p13	BC034773, DA094549,	100507495	ENSG00000234684		NR_040047
HGNC:44003	SEC24B-AS1	4q25	BC009800	100533182	ENSG00000247950	21307942	NR_039978
HGNC:40586	SEC62-AS1	3q26.2			ENSG00000240373		
HGNC:49096	SEMA3B-AS1	3p21.31		101928931	ENSG00000232352		
HGNC:40518	SEMA3F-AS1	3p21.31	AK125500	100129060	ENSG00000235016		
HGNC:51110	SEMA6A-AS1	5q23.1		101927233	ENSG00000248445		
HGNC:44177	SENCR	11q24.3	BC039676	100507392	ENSG00000254703	24578380	NR_038908
HGNC:27737	SEPSECS-AS1	4p15.2	AK095450	285540			NR_037934
HGNC:51345	SEPT4-AS1	17q22	CK429595, BX092197,	101927688	ENSG00000264672		NR_110810
HGNC:51153	SEPT7-AS1	7p14.2		101928545	ENSG00000228878		
HGNC:32019	SERTAD4-AS1	1q32.2	BC066352	574036	ENSG00000203706		NR_024337
HGNC:51589	SFTPD-AS1	10q22.3	AK124605		ENSG00000273372		
HGNC:49683	SGMS1-AS1	10q11.23			ENSG00000226200	24495672	
HGNC:41081	SGO1-AS1	3p24.3			ENSG00000237485		
HGNC:44501	SH3BP5-AS1	3p25.1		100505696	ENSG00000224660		NR_046084
HGNC:45242	SH3PXD2A-AS1	10q24.33	AK056784	100505839			NR_038940
HGNC:44168	SH3RF3-AS1	2q12.3		100287216	ENSG00000259863		NR_029193
HGNC:40014	SHANK2-AS1	11q13.4			ENSG00000226627		
HGNC:40015	SHANK2-AS2	11q13.4			ENSG00000236262		
HGNC:25098	SHANK2-AS3	11q13.4	BC004224	220070	ENSG00000171671	12477932	
HGNC:40526	SIAH2-AS1	3q25.1			ENSG00000244265		
HGNC:41126	SIDT1-AS1	3q13.2			ENSG00000239453		
HGNC:41495	SIK3-IT1	11q23.3			ENSG00000231865		
HGNC:51229	SIRPG-AS1	20p13	AK093519	101929010	ENSG00000237914		NR_110090
HGNC:51912	SIRT1-AS	10q21.3				26324025	
HGNC:40532	SIX3-AS1	2p21	BM690547, BM663835	100506108	ENSG00000236502		
HGNC:44187	SLC2A1-AS1	1p34.2	AK056786	440584	ENSG00000227533		NR_033967
HGNC:40546	SLC6A1-AS1	3p25.3			ENSG00000232287		
HGNC:44064	SLC7A11-AS1	4q28.3		641364	ENSG00000250033		NR_038380
HGNC:44102	SLC8A1-AS1	2p22.1		100128590	ENSG00000227028		
HGNC:40928	SLC9A9-AS1	3q24			ENSG00000240012		
HGNC:40929	SLC9A9-AS2	3q24			ENSG00000244493		

HGNC:51125	SLC14A2-AS1	18q12.3		101927980	ENSG00000267097		
HGNC:49445	SLC16A1-AS1	1p13.2		100506392	ENSG00000226419		
HGNC:51205	SLC16A12-AS1	10q23.31		101926906	ENSG00000234452		NR_120614
HGNC:43438	SLC25A5-AS1	Xq24		100303728	ENSG00000224281		NR_028443
HGNC:44298	SLC25A21-AS1	14q13.3		100129794	ENSG00000258708		
HGNC:27844	SLC25A25-AS1	9q34.11	AL833509, BU623777,	100289019	ENSG00000234771		NR_033374
HGNC:42795	SLC25A30-AS1	13q14.13			ENSG00000251015		
HGNC:22385	SLC26A4-AS1	7q22.3	BC037315	286002	ENSG00000233705		NR_028137
HGNC:44881	SLC39A12-AS1	10p12.33		100129213	ENSG00000226083		NM_00114519
HGNC:40537	SLCO4A1-AS1	20q13.33		100127888	ENSG00000232803		
HGNC:44126	SLFNL1-AS1	1p34.2	AL157461	100507178			NR_037868
HGNC:51198	SLIT1-AS1	10q24.1		100505540	ENSG00000234855		NR_038330
HGNC:41383	SLIT2-IT1	4p15.31		100505893	ENSG00000248228		
HGNC:49379	SMAD1-AS1	4q31.21	AW087665		ENSG00000250902	24147006	
HGNC:49381	SMAD1-AS2	4q31.21	BC042377	101927659	ENSG00000250582		
HGNC:30586	SMAD5-AS1	5q31.1	AF086556	9597	ENSG00000164621	10049768	
HGNC:39963	SMAD9-IT1	13q13.3			ENSG00000236711		
HGNC:39982	SMARCA5-AS1	4q31.21		100128055	ENSG00000245112		
HGNC:50827	SMC2-AS1	9q31.1		101928550	ENSG00000270332		
HGNC:48718	SMC5-AS1	9q21.12		100507299	ENSG00000268364		
HGNC:17914	SMCR2	17p11.2	AI821758	105371564	ENSG00000223979	11997338	NR_131243
HGNC:17918	SMCR5	17p11.2		140771		11997338	NR_024007
HGNC:17919	SMCR6	17p11.2	AJ230805, BF511382, V	140772		11997338	
HGNC:1220	SMG6-IT1	17p13.3	AJ276246	53400			
HGNC:24518	SMG7-AS1	1q25.3	BC032873	284649	ENSG00000232860	12477932	NR_040063
HGNC:42674	SMIM2-AS1	13q14.11		101929212	ENSG00000227258		
HGNC:41492	SMIM2-IT1	13q14.11	AI476676	100874377	ENSG00000235285		
HGNC:41406	SMYD3-IT1	1q44			ENSG00000230184		
HGNC:28327	SNAI3-AS1	16q24.3		197187	ENSG00000260630	12477932	
HGNC:44312	SNAP25-AS1	20p12.2	AK124961, BF244758,	100131208	ENSG00000227906		NR_040710
HGNC:41501	SNAP47-AS1	1q42.13	AK057311		ENSG00000230005		
HGNC:50600	SNCA-AS1	4q22.1		644248	ENSG00000247775		
HGNC:24158	SND1-IT1	7q32.1	AF191492	27099			NR_027330
HGNC:32688	SNHG1	11q12.3	L36588	23642	ENSG00000255717	8559254, 23801869, 2	NR_003098
HGNC:10118	SNHG3	1p35.3	AJ006834	8420	ENSG00000242125	9671460	NR_002909.1
HGNC:32964	SNHG4	5q31.2		724102		9630250	NR_003141
HGNC:21026	SNHG5	6q14.3	BC009220	387066	ENSG00000203875	10792466	NR_003038
HGNC:32965	SNHG6	8q13	AY264285	641638	ENSG00000245910	16226852	NR_002599
HGNC:28254	SNHG7	9q34.3	CR599965, BC042369	84973	ENSG00000233016	12477932	NR_003672
HGNC:33098	SNHG8	4q26	BC017993, CR625561,	100093630	ENSG00000269893	15199136	NR_003584
HGNC:33102	SNHG9	16p13.3	CR749453, BC028232	735301	ENSG00000255198		NR_003142
HGNC:27510	SNHG10	14q32.13		283596	ENSG00000247092	12477932	NR_001459
HGNC:25046	SNHG11	20q11.23	AF497716	128439	ENSG00000174365	12477932	NR_003239
HGNC:30062	SNHG12	1p35.3	AF277181, KJ782215	85028	ENSG00000197989	25695231	NR_024127
HGNC:37462	SNHG14	15q11.2			ENSG00000224078	23771028	
HGNC:27797	SNHG15	7p13	BC092459, AK096179	285958	ENSG00000232956	14702039, 24036268	NR_003697
HGNC:44352	SNHG16	17q25.1	BC042949, BC100293,	100507246	ENSG00000163597	19287950, 21147498,	NR_038108
HGNC:48600	SNHG17	20q11.23	BC032119, BC052370	388796	ENSG00000196756		NR_015366
HGNC:49007	SNHG18	5p15.31		100505806	ENSG00000250786		NR_045196
HGNC:49574	SNHG19	16p13.3		100507303	ENSG00000260260		
HGNC:33099	SNHG20	17q25.2	AF130050	654434	ENSG00000234912	16373490	
HGNC:50284	SNHG21	15q25.2	AA284521, BX112407	100505616	ENSG00000250988		NR_110096
HGNC:50285	SNHG22	18q21.1	AK123464	103091864	ENSG00000267322		NR_117096
HGNC:50622	SNHG23	14q32.31	BX508291	100507242	ENSG00000225746		

HGNC:50825	SNHG24	14q32.31		101929369	ENSG00000271417		NR_110178
HGNC:51534	SNHG25	17q23.3			ENSG00000266402		
HGNC:41269	SNRK-AS1	3p22.1			ENSG00000234617		
HGNC:27054	SOCS2-AS1	12q22		144481	ENSG00000246985	24168098	NR_038263
HGNC:45244	SORCS3-AS1	10q25.1	BC042079	100505890	ENSG00000226387		
HGNC:41385	SOS1-IT1	2p22.1		100505911	ENSG00000229692		
HGNC:20209	SOX2-OT	3q26.33	AL157425, AK022826	347689	ENSG00000242808	12612584, 19767420	NR_004053
HGNC:49321	SOX9-AS1	17q24.3		400618	ENSG00000234899		
HGNC:39807	SOX21-AS1	13q32.1		100507533	ENSG00000227640	25959816	NR_046514
HGNC:51341	SP2-AS1	17q21.32	BC038442, BF673352, I	100506325	ENSG00000234494		NR_103856
HGNC:49383	SPACA6P-AS	19q13.41	AK125996		ENSG00000269959		
HGNC:41140	SPAG5-AS1	17q11.2		100506436	ENSG00000227543		NR_040012
HGNC:31683	SPANXA2-OT1	Xq27.2	AK093505	619455	ENSG00000277215		NR_037183
HGNC:28013	SPATA3-AS1	2q37.1		348761	ENSG00000238062	12477932	
HGNC:48627	SPATA8-AS1	15q26.2		100652749	ENSG00000259282		
HGNC:39905	SPATA13-AS1	13q12.12			ENSG00000227213		
HGNC:41086	SPATA17-AS1	1q41	AI025201		ENSG00000234070		
HGNC:48613	SPATA41	15q26.3	AK057536, AK124283,	388182	ENSG00000189419		NR_028139
HGNC:41198	SPATA42	1p13.3		642864	ENSG00000203897		
HGNC:39933	SPG20-AS1	13q13.3		100507135	ENSG00000120664		NR_045180
HGNC:41177	SPIN4-AS1	Xq11.1			ENSG00000233661		
HGNC:42394	SPRY4-IT1	5q31.3	AK024556	100642175		21558391, 26829028	
HGNC:44122	SPTY2D1-AS1	11p15.1	AK097654	100506540	ENSG00000247595		NR_038360
HGNC:44138	SRD5A3-AS1	4q12	BC034006	100506462	ENSG00000249700		NR_037969
HGNC:40902	SRGAP2-AS1	1q32.1	AK127439		ENSG00000233501		
HGNC:40898	SRGAP3-AS1	3p25.3	AI209215, BX111651		ENSG00000224808		
HGNC:40899	SRGAP3-AS2	3p25.3		100507660	ENSG00000228723		
HGNC:40900	SRGAP3-AS3	3p25.3		100288831	ENSG00000227929		
HGNC:42434	SRGAP3-AS4	3p25.3			ENSG00000235830		
HGNC:48619	SRP14-AS1	15q15.1		100131089	ENSG00000248508		
HGNC:50643	SRP54-AS1	14q13.2		100506157	ENSG00000258704		
HGNC:44162	SRRM2-AS1	16p13.3	AK056063	100128788	ENSG00000205913		NR_027274
HGNC:32328	SSBP3-AS1	1p32.3	AF176918	619518	ENSG00000198711		
HGNC:27384	SSSCA1-AS1	11q13.1		254100	ENSG00000260233		
HGNC:26502	SSTR5-AS1	16p13.3	AK056814	146336	ENSG00000261713		NR_02724
HGNC:48645	ST3GAL4-AS1	11q24.2		399972			
HGNC:51129	ST3GAL5-AS1	2p11.2		101928113	ENSG00000232504		
HGNC:40828	ST3GAL6-AS1	3q12.1			ENSG00000239445		
HGNC:41380	ST6GAL2-IT1	2q12.2			ENSG00000238250		
HGNC:16000	ST7-AS1	7q31.2	AF400039	93653	ENSG00000227199	12213198	NR_002330
HGNC:16044	ST7-AS2	7q31.2	AF400043	93654	ENSG00000226367	12213198	NR_002331
HGNC:16045	ST7-OT3	7q31.3	AF400044	93655		12213198	NR_002332
HGNC:44880	ST8SIA6-AS1	10p12.33		100128098	ENSG00000204832		NR_034129
HGNC:27521	ST20-AS1	15q25.1	AK091107	283687	ENSG00000259642		NR_028330
HGNC:49568	STAM-AS1	10p12.33	AK123677		ENSG00000260589	23382218	
HGNC:44117	STARD4-AS1	5q22.1	CR749489	100505678	ENSG00000246859		NR_040093
HGNC:40827	STARD7-AS1	2q11.2		285033	ENSG00000204685		
HGNC:40873	STARD13-AS	13q12-q13		100874241	ENSG00000236581		NR_046693
HGNC:41453	STARD13-IT1	13q13.1			ENSG00000230300		
HGNC:44101	STAU2-AS1	8q21.11	AK056720	100128126	ENSG00000253302		NR_038406
HGNC:40820	STEAP2-AS1	7q21.13			ENSG00000227646		
HGNC:41053	STEAP3-AS1	2q14.2			ENSG00000229867		
HGNC:43811	STK4-AS1	20q13.12		100505826	ENSG00000227477		NR_038341
HGNC:39935	STK24-AS1	13q32.2			ENSG00000224418		

HGNC:41209	STPG2-AS1	4q22.3	DA719663		ENSG00000251620		
HGNC:44585	STT3A-AS1	11q24.2	DB470444, DB453285		ENSG00000254671		
HGNC:51174	STX17-AS1	9q31.1	BC030123	441461	ENSG00000255145		NR_038853
HGNC:48877	STX18-AS1	4p16.2		100507266	ENSG00000247708	23708191	
HGNC:41415	STX18-IT1	4p16.3			ENSG00000248221		
HGNC:44183	STXBP5-AS1	6q24.3	BC044307	729178	ENSG00000233452		NR_034115
HGNC:39965	SUCLA2-AS1	13q14.2			ENSG00000227848		
HGNC:49643	SUCLG2-AS1	3p14.1		101927111	ENSG00000241316		
HGNC:51219	SVIL-AS1	10p11.23		102724316	ENSG00000224597		NR_110920
HGNC:40793	SYNE1-AS1	6q25.2	CK821189, BI962584, B	100505475	ENSG00000234577		
HGNC:41387	SYNJ2-IT1	6q25.3			ENSG00000233496		
HGNC:40774	SYNPR-AS1	3p14.2			ENSG00000241359		
HGNC:40571	SYP-AS1	Xp11.23			ENSG00000237341		
HGNC:41225	SZT2-AS1	1p34.2			ENSG00000229372		
HGNC:20176	TAB3-AS1	Xp21.2		727682	ENSG00000231542		
HGNC:40013	TAB3-AS2	Xp21.2	BI002962		ENSG00000235512		
HGNC:40573	TAF1A-AS1	1q41		100506161	ENSG00000225265		
HGNC:26832	TAPT1-AS1	4p15.32		202020	ENSG00000263327		NR_027696
HGNC:50506	TARID	6q23.2		100507308	ENSG00000227954	25087872	
HGNC:51369	TAT-AS1	16q22.2	HY017421, BU568247,	100132529	ENSG00000260886		NR_103851
HGNC:51221	TBC1D22A-AS1	22q13.31	AK057318	642757			NR_122047
HGNC:41243	TBL1XR1-AS1	3q26.32			ENSG00000231310		
HGNC:50355	TBX2-AS1	17q23.2	AI927936		ENSG00000267280		
HGNC:27402	TBX5-AS1	12q24.21		255480	ENSG00000255399		NR_038440
HGNC:52256	TBX18-AS1	6q14.3		102724201	ENSG00000228290		NR_125875
HGNC:41191	TCEAL3-AS1	Xq22.2	AV651489		ENSG00000224031		
HGNC:50582	TCEB3-AS1	1p36.11		100506963	ENSG00000236810		
HGNC:49532	TCERG1L-AS1	10q26.3	BQ007413, BQ011025,	101927489	ENSG00000230098		XR_242764
HGNC:51642	TCF4-AS1	18q21.2			ENSG00000267028		
HGNC:51643	TCF4-AS2	18q21.2			ENSG00000267402		
HGNC:41388	TCF7L1-IT1	2p11.2			ENSG00000231134		
HGNC:13463	TCL6	14q32.1	AB035338	27004	ENSG00000187621	10588720, 10851082	NM_012468
HGNC:43642	TDRG1	6p21.2	DQ168992	732253	ENSG00000204091	19403381, 22123530,	NR_024015
HGNC:51142	TESC-AS1	12q24.22		101928244	ENSG00000258285		
HGNC:41125	TET2-AS1	4q24			ENSG00000251586		
HGNC:42784	TEX26-AS1	13q12.3	BC042736	100507064			NR_038288
HGNC:49500	TEX36-AS1	10q26.13		100169752	ENSG00000237675		NR_023362
HGNC:48667	TEX41	2q22.3	BC043549	401014	ENSG00000226674		NR_033870
HGNC:40579	TFAP2A-AS1	6p24.3		100130275	ENSG00000229950		
HGNC:41389	TGFA-IT1	2p13.3			ENSG00000224606		
HGNC:50628	TGFB2-AS1	1q41	BX099841	728463	ENSG00000232480		NR_046268
HGNC:50629	TGFB2-OT1	1q41	AK021874	103611157	ENSG00000281453	24879147, 25437332	NR_125715
HGNC:40495	TH2LCRR	5q31.1		101927761	ENSG00000223442	25903499	NR_132124
HGNC:41013	THAP7-AS1	22q11.21		439931	ENSG00000230513		NR_027051
HGNC:44172	THAP9-AS1	4q21.22	BC063702	100499177	ENSG00000251022		NR_034075
HGNC:41249	THOC7-AS1	3p14.1			ENSG00000240549		
HGNC:44515	THRB-AS1	3p24.2		644990	ENSG00000228791		NR_046244
HGNC:41390	THRB-IT1	3p24.2			ENSG00000224822		
HGNC:49503	THRIL	12q24.31	AK025766	102659353		24371310	NR_110375
HGNC:51420	THSD4-AS1	15q23	DA243226, DA296094	101929196	ENSG00000259964		NR_120348
HGNC:51421	THSD4-AS2	15q23	AK055527	101929173			NR_120346
HGNC:44478	THUMPD3-AS1	3p25.3	BC052961	440944	ENSG00000206573		NR_027007
HGNC:14607	TINCR	19p13.3	BG354568	257000	ENSG00000223573	23201690, 24019000	NR_027064
HGNC:41028	TIPARP-AS1	3q25.31		100287227	ENSG00000243926		NR_027954

HGNC:40720	TLR8-AS1	Xp22.2		349408	ENSG00000233338		
HGNC:37183	TLX1NB	10q24	BC019674	100038246	ENSG00000236311	17303350	NM_00108539
HGNC:40587	TM4SF1-AS1	3q25.1			ENSG00000240541		
HGNC:41085	TM4SF19-AS1	3q29			ENSG00000235897		
HGNC:51424	TMC3-AS1	15q25.1	HY112626, CB134362	101929655	ENSG00000259343		NR_120365
HGNC:49060	TMCC1-AS1	3q22.1		100507032	ENSG00000271270		
HGNC:48910	TMEM5-AS1	12q14.2	DB026756		ENSG00000255850		
HGNC:19230	TMEM9B-AS1	11p15.3	AJ400877	493900	ENSG00000254860	11528127	
HGNC:51209	TMEM26-AS1	10q21.2	BC041470	101928781	ENSG00000237233		NR_120643
HGNC:44272	TMEM44-AS1	3q29	BE071707, BI088778, B	100507297	ENSG00000231770		NR_047573
HGNC:26301	TMEM51-AS1	1p36.21	AK074283	200197	ENSG00000175147		NR_027136
HGNC:27349	TMEM72-AS1	10q11.21		220980	ENSG00000224812		NR_033842
HGNC:50442	TMEM92-AS1	17q21.33	BU687974		ENSG00000251179	24768205	
HGNC:50307	TMEM108-AS1	3q22.1		101927455	ENSG00000251011		
HGNC:51273	TMEM147-AS1	19q13.12	CR627053	100506469	ENSG00000236144		NR_038396
HGNC:43839	TMEM161B-AS1	5q14.3	AK082072	100505894	ENSG00000247828	21890647	NR_039993
HGNC:41939	TMEM212-AS1	3q26.31			ENSG00000234717		
HGNC:41505	TMEM212-IT1	3q26.31			ENSG00000235943		
HGNC:44357	TMEM220-AS1	17p13.1-p12	DA190285	101101775	ENSG00000263400		
HGNC:51191	TMEM246-AS1	9q31.1		101928470	ENSG00000225376		NR_121573
HGNC:27340	TMEM254-AS1	10q22.3		219347	ENSG00000230091		
HGNC:44261	TMLHE-AS1	Xq28	BC042086	100507404	ENSG00000224533		NR_039991
HGNC:44158	TMPO-AS1	12q23.1		100128191	ENSG00000257167		NR_027157
HGNC:44179	TMPRSS4-AS1	11q23.3		100526771	ENSG00000255274		NR_038318
HGNC:49093	TNK2-AS1	3q29	AK127609	100128262	ENSG00000224614		
HGNC:45173	TNKS2-AS1	10q23.32	AA776692	100507633	ENSG00000228701		
HGNC:41391	TNR-IT1	1q25.1			ENSG00000235628		
HGNC:44360	TNRC6C-AS1	17q25.3		100131096	ENSG00000204282		
HGNC:44340	TOB1-AS1	17q21.33	BC039664, BP421960, I	400604	ENSG00000229980		NR_038458
HGNC:27403	TOLLIP-AS1	11p15.5		255512	ENSG00000255153	12477932	
HGNC:51556	TONSL-AS1	8q24.3		100287098	ENSG00000232600		NR_109770
HGNC:31420	TOPORS-AS1	9p21.1		100129250	ENSG00000235453		NR_033991
HGNC:43652	TP53COR1	6p21.2	CD515754	102800311		20673990, 22841487, 24381249	
HGNC:17026	TP53TG1	7q21.1	AB007455	11257	ENSG00000182165	9713990	
HGNC:29052	TP73-AS1	1p36.32		57212	ENSG00000227372	9455484, 20477830, 2	NR_033708
HGNC:42391	TPRG1-AS1	3q28			ENSG00000234076		
HGNC:41062	TPRG1-AS2	3q28			ENSG00000230115		
HGNC:43686	TPT1-AS1	13q14.13	AF318337	100190939	ENSG00000170919		NR_024458
HGNC:40005	TRAF3IP2-AS1	6q21		643749	ENSG00000231889		NR_034108
HGNC:48663	TRAM2-AS1	6p12.2		401264	ENSG00000225791		
HGNC:41046	TRAPPC12-AS1	2p25.3	BG683264		ENSG00000225234		
HGNC:44307	TRERNA1	20q13.13	AI968488, BX118093	100887755	ENSG00000231265	20887892, 23974796	NR_051976
HGNC:48974	TRG-AS1	7p14.1		100506776			NR_040085
HGNC:27471	TRHDE-AS1	12q21.1	BC040230	283392	ENSG00000236333		NR_026836
HGNC:39761	TRIM31-AS1	6p22.1			ENSG00000231226		
HGNC:41422	TRIM36-IT1	5q22.3			ENSG00000250472		
HGNC:49006	TRIM52-AS1	5q35.3		100507602	ENSG00000248275		
HGNC:41116	TRMT2B-AS1	Xq22.1			ENSG00000225839		
HGNC:40936	TRPC7-AS1	5q31.1			ENSG00000248211		
HGNC:40937	TRPC7-AS2	5q31.1			ENSG00000250947		
HGNC:50758	TRPM2-AS	21q22.3	AK057275	101928607	ENSG00000230061	18957938, 24931166	NR_109964
HGNC:43684	TSC22D1-AS1	13q14.11	BC040675	641467			NR_038381
HGNC:12377	TSIX	Xq13.2		9383		10192391	NR_003255
HGNC:41462	TSPAN9-IT1	12p13.33			ENSG00000256197		

HGNC:1271	TSPEAR-AS1	21q22.3		54082	ENSG00000235890		
HGNC:16428	TSPEAR-AS2	21q22.3	AF426270	114043	ENSG00000182912	12036297	NR_026547
HGNC:41392	TSSC1-IT1	2p25.3			ENSG00000224885		
HGNC:40595	TTC3-AS1	21q22.13	BF979681		ENSG00000228677		
HGNC:41115	TTC21B-AS1	2q24.3		100506134	ENSG00000224490		
HGNC:29336	TTC28-AS1	22q12.1		284900	ENSG00000235954	11258795	NR_026962
HGNC:40851	TTC39A-AS1	1p32.3	CB128747		ENSG00000261664		
HGNC:51321	TTC39C-AS1	18q11.2	DB448536, DB513641,	102724246	ENSG00000264745		NR_110796
HGNC:41486	TTLL7-IT1	1p31.1			ENSG00000233061		
HGNC:41159	TTLL10-AS1	1p36.33	AK128833	100506376	ENSG00000205231		
HGNC:24214	TTLL11-IT1	9q34.11		401550	ENSG00000237548		
HGNC:44124	TTN-AS1	2q31.2	BC041357	100506866	ENSG00000237298		NR_038272
HGNC:14022	TTTY1	Yp11.2	AF000990	50858	ENSG00000129845		NR_001538
HGNC:37981	TTTY1B	Yp11.2		100101116	ENSG00000129816		NR_003589
HGNC:14023	TTTY2	Yp11.2	AF000991	60439	ENSG00000212855		NR_001536
HGNC:37982	TTTY2B	Yp11.2		100101117	ENSG00000212856		NR_003590
HGNC:16480	TTTY3	Yq11.23	AF332230	114760	ENSG00000231141		NR_001524
HGNC:31888	TTTY3B	Yq11.23		474148	ENSG00000280961		NR_002176
HGNC:16481	TTTY4	Yq11.2	AF332231	114761	ENSG00000226906		NR_001525
HGNC:31891	TTTY4B	Yq11.223		474149	ENSG00000235412		NR_002178
HGNC:31892	TTTY4C	Yq11.223		474150	ENSG00000228296		NR_002177
HGNC:16482	TTTY5	Yq11.222	AF332236	83863	ENSG00000215560		NR_001541
HGNC:16483	TTTY6	Yq11.223	AF332237	84672	ENSG00000131538		NR_001527
HGNC:31887	TTTY6B	Yq11.223	AY597808	441543	ENSG00000131548		NR_002175
HGNC:18488	TTTY7	Yp11.2	AF332232	246122	ENSG00000147753		NR_001534
HGNC:37463	TTTY7B	Yp11.2	BX089948	100101120	ENSG00000147761		NR_003592
HGNC:18489	TTTY8	Yp11.2	AF332234	84673	ENSG00000183385		NR_001533
HGNC:37622	TTTY8B	Yp11.2		100101118	ENSG00000185700		NR_003591
HGNC:18490	TTTY9A	Yq11.222	AF332238	83864			NR_001530
HGNC:32425	TTTY9B	Yq11.221		425057	ENSG00000131007	12815422	NR_002159
HGNC:18491	TTTY10	Yq11.221	AF332239	246119	ENSG00000229236		NR_001542
HGNC:18492	TTTY11	Yp11.2	AF332240	83866	ENSG00000180910		NR_001548
HGNC:18493	TTTY12	Yp11.2	AF332241	83867	ENSG00000237048		NR_001551
HGNC:18494	TTTY13	Yp11.2	AF332242	83868	ENSG00000184991		NR_001537
HGNC:38596	TTTY13B	Yp11.2		100287735			
HGNC:18495	TTTY14	Yq11.222	AF332243	83869	ENSG00000176728		NR_001543
HGNC:18567	TTTY15	Yq11.1	AL080135	64595	ENSG00000233864		NR_001545
HGNC:18840	TTTY16	Yp11.2		252948	ENSG00000225520		NR_001552
HGNC:18841	TTTY17A	Yq11.2	AF527829	252949	ENSG00000228240		NR_001526
HGNC:31889	TTTY17B	Yq11.223		474151	ENSG00000227439		NR_002180
HGNC:31890	TTTY17C	Yq11.223		474152	ENSG00000223641		NR_002179
HGNC:18842	TTTY18	Yp11.2		252950	ENSG00000233699		NR_001550
HGNC:18843	TTTY19	Yp11.2		252952	ENSG00000232419		NR_001549
HGNC:18844	TTTY20	Yp11.2		252951	ENSG00000232808		NR_001546
HGNC:18845	TTTY21	Yp11.2		252953	ENSG00000228890		NR_001535
HGNC:37980	TTTY21B	Yp11.2		100101115	ENSG00000237563		NR_003588
HGNC:18846	TTTY22	Yp11.2		252954	ENSG00000224075		NR_001539
HGNC:18847	TTTY23	Yp11.2		252955	ENSG00000239225		NR_001540
HGNC:37983	TTTY23B	Yp11.2		100101121	ENSG00000237069		NR_003593
HGNC:51120	TUB-AS1	11p15.4		101927917	ENSG00000248332		
HGNC:26066	TUG1	22q12.2	AK057695	55000	ENSG00000253352	15797018, 22961206,	NR_002323
HGNC:44088	TUNAR	14q32.2	AK023027	100507043	ENSG00000250366	24530304, 22196729,	NR_038861
HGNC:27701	TUSC7	3q13.31	BC035185	285194	ENSG00000243197	20048075, 23680400	NR_015391
HGNC:49111	TUSC8	13q14.11	BC025370	400128	ENSG00000237361	24667250	NR_104174

HGNC:30008	TXNDC12-AS1	1p32.3	AI221303		ENSG00000228369		
HGNC:49083	UBA6-AS1	4q13.2		550112	ENSG00000248049		
HGNC:42502	UBAC2-AS1	13q32.3		100289373	ENSG00000228889		
HGNC:40598	UBE2E1-AS1	3p24.3			ENSG00000223791		
HGNC:44512	UBE2E2-AS1	3p24.3		100505877	ENSG00000233153		
HGNC:40722	UBE2Q1-AS1	1q21.3	AW070285		ENSG00000229780		
HGNC:49911	UBE2R2-AS1	9p13.3	HG501976	101929665			
HGNC:48614	UBL7-AS1	15q24.1		440288	ENSG00000247240		
HGNC:44111	UBOX5-AS1	20p13	AK097516	100134015	ENSG00000235958		NR_038395
HGNC:51661	UBR5-AS1	8q22.3		101927221	ENSG00000246263		NR_125415
HGNC:41227	UBXN7-AS1	3q29			ENSG00000225822		
HGNC:41141	UBXN10-AS1	1p36.12	AI288607	101928017	ENSG00000225986		
HGNC:37126	UCA1	19p13.12	BC005351	652995	ENSG00000214049	18501714, 17416635,	NR_015379
HGNC:40600	UCHL1-AS1	4p13	DB169394, CN277289		ENSG00000251173		
HGNC:31967	UCKL1-AS1	20q13.33	AK128497	100113386			NR_027287
HGNC:41007	UFL1-AS1	6q16.1	BM126999, BM127608		ENSG00000233797		
HGNC:40601	UGDH-AS1	4p14			ENSG00000249348		
HGNC:23821	UMODL1-AS1	21q22.3		150147	ENSG00000184385		NR_027243
HGNC:45096	UNC5B-AS1	10q22.1		728978	ENSG00000237512		NR_038453
HGNC:40603	UPK1A-AS1	19q13.12	BC007817		ENSG00000226510		
HGNC:41467	UPP2-IT1	2q24.1			ENSG00000237327		
HGNC:23128	URB1-AS1	21q22.11	BC007928	84996	ENSG00000256073		NR_026845
HGNC:48673	USP2-AS1	11q23.3		100499227	ENSG00000245248		
HGNC:44140	USP3-AS1	15q22.31	AK093077	100130855	ENSG00000259248		NR_034080
HGNC:39961	USP12-AS1	13q12.13			ENSG00000232162		
HGNC:39962	USP12-AS2	13q12.13			ENSG00000230641		
HGNC:27249	USP27X-AS1	Xp11.23		158572	ENSG00000234390		
HGNC:40909	USP30-AS1	12q24.11		100131733	ENSG00000256262		NR_038996
HGNC:43991	USP46-AS1	4q12	BX354350, CN313378	643783	ENSG00000248866		
HGNC:40802	UST-AS1	6q25.1		100128176	ENSG00000227660		NR_038408
HGNC:49239	UXT-AS1	Xp11.23		100133957	ENSG00000267064		
HGNC:48605	VAC14-AS1	16q22.2	AK095020, DB487352	100130894	ENSG00000214353		NR_034083
HGNC:40608	VAV3-AS1	1p13.3	AA425131, BG193383		ENSG00000230489		
HGNC:40163	VCAN-AS1	5q14.3			ENSG00000249835		
HGNC:44879	VIM-AS1	10p13	BC078172	100507347	ENSG00000229124		
HGNC:40610	VIPR1-AS1	3p22.1			ENSG00000232354		
HGNC:49621	VLDLR-AS1	9p24.2	AK092343, BC004474	401491	ENSG00000236404		NR_015375
HGNC:48915	VPS9D1-AS1	16q24.3		100128881	ENSG00000261373		
HGNC:44167	VPS13A-AS1	9q21.2	AA954471, DB509755	100286938	ENSG00000232998		NR_026668
HGNC:50770	VSTM2A-OT1	7p11.2		285878	ENSG00000224223		
HGNC:44270	VWA8-AS1	13q14.11	AK092199, CA310831	100507240			NR_039974
HGNC:41508	VWC2L-IT1	2q35			ENSG00000224257		
HGNC:27347	WAC-AS1	10p12.1		220906	ENSG00000254635	12477932	
HGNC:41393	WARS2-IT1	1p12			ENSG00000224238		
HGNC:39841	WASF3-AS1	13q12.13			ENSG00000237001		
HGNC:38513	WASIR1	Xq28 and Yq	AJ271736	100128260	ENSG00000185203		
HGNC:38609	WASIR2	16p13.3	BC032901, CR605219	100132169	ENSG00000231439	11157797	XR_078518
HGNC:40935	WDFY3-AS1	4q21.23			ENSG00000251260		
HGNC:21603	WDFY3-AS2	4q21.3	AY250185	404201	ENSG00000180769	15580561	NR_015359
HGNC:45131	WDR7-OT1	18q21.31	DA634713		ENSG00000267225		
HGNC:27437	WDR11-AS1	10q26.12	AK094649	283089	ENSG00000227165		NR_033850
HGNC:41186	WDR86-AS1	7q36.1	AK095986	100131176	ENSG00000243836		NR_034012
HGNC:48669	WEE2-AS1	7q34		285962	ENSG00000228775		
HGNC:49126	WISP1-OT1	8q24.22	BQ009791		ENSG00000270132		

HGNC:40616	WNT5A-AS1	3p14.3			ENSG00000244586		
HGNC:51639	WSPAR	5q31.1	BE270324		ENSG00000249073	25842979	
HGNC:18135	WT1-AS	11p13	BC002734	51352	ENSG00000183242	2173145, 8406502, 17	NR_023920
HGNC:41041	WWC2-AS1	4q35.1			ENSG00000251128		
HGNC:26390	WWC2-AS2	4q35.1	AK054839	152641	ENSG00000251359		
HGNC:41236	WWC3-AS1	Xp22.2			ENSG00000225076		
HGNC:41035	WWTR1-AS1	3q25.1		100128025	ENSG00000241313		
HGNC:41469	WWTR1-IT1	3q25.1			ENSG00000241985		
HGNC:45056	XACT	Xq23	BQ010753	105463123	ENSG00000241743	23334669, 25921272	NR_131204
HGNC:40078	XIAP-AS1	Xq25			ENSG00000237331		
HGNC:40679	XIRP2-AS1	2q24.3			ENSG00000254552		
HGNC:12810	XIST	Xq13.2	M97168	7503	ENSG00000229807	1985261, 2034279	NR_001564
HGNC:41153	XXYLT1-AS1	3q29	BF509707		ENSG00000233303		
HGNC:41154	XXYLT1-AS2	3q29	R83154, AW963078		ENSG00000230266		
HGNC:49034	YAM1	16p11.2				23942234	
HGNC:41101	YEATS2-AS1	3q27.1			ENSG00000233885		
HGNC:48728	YTHDF3-AS1	8q12.3	BM681776, BM967225	101410533	ENSG00000270673		
HGNC:44188	ZBED3-AS1	5q13.3		728723	ENSG00000250802	25482444	NR_024398
HGNC:48646	ZBED5-AS1	11p15.3		729013	ENSG00000247271		
HGNC:48573	ZBTB11-AS1	3q12.3		100009676	ENSG00000256628		
HGNC:40640	ZBTB20-AS1	3q13.31		100131117	ENSG00000241560		
HGNC:42421	ZBTB20-AS2	3q13.31		100506673	ENSG00000241295		
HGNC:42422	ZBTB20-AS3	3q13.31			ENSG00000239946		
HGNC:42423	ZBTB20-AS4	3q13.31			ENSG00000242767		
HGNC:41493	ZBTB40-IT1	1p36.12			ENSG00000237200		
HGNC:40753	ZBTB46-AS1	20q13.33		101928604	ENSG00000231208		
HGNC:39900	ZDHHC20-IT1	13q12.11			ENSG00000236953		
HGNC:42354	ZEB1-AS1	10p11.22		220930	ENSG00000237036		
HGNC:37149	ZEB2-AS1	2q22.3		100303491	ENSG00000238057	18347095, 26152796	
HGNC:33101	ZFAS1	20q13.13	AY513722, AY513723	441951	ENSG00000177410	21460236	NR_003604
HGNC:33992	ZFAT-AS1	8q24.22	AB167742	594840	ENSG00000248492	15294872, 22894909	NR_002438
HGNC:44165	ZFHX4-AS1	8q21.11	BC037827	100192378	ENSG00000253661		NR_024360
HGNC:50698	ZFPM2-AS1	8q23.1	BF244158		ENSG00000251003		
HGNC:40617	ZFX-AS1	Xp22.11			ENSG00000234230		
HGNC:38798	ZFY-AS1	Yp11.31		100506003	ENSG00000233070		
HGNC:40920	ZIC4-AS1	3q24			ENSG00000241202		
HGNC:51304	ZIM2-AS1	19q13.43		101929059	ENSG00000269793	19539734	NR_110744
HGNC:27433	ZMIZ1-AS1	10q22.3		283050	ENSG00000224596		NR_024431
HGNC:40624	ZMYM4-AS1	1p34.3	BG187713, BG208679		ENSG00000227409		
HGNC:40890	ZMYND10-AS1	3p21.31			ENSG00000235058		
HGNC:51179	ZNF30-AS1	19q13.11		102723513	ENSG00000270876		NR_110749
HGNC:23577	ZNF32-AS1	10q11.21			ENSG00000226245		
HGNC:23593	ZNF32-AS2	10q11.21			ENSG00000230565		
HGNC:23583	ZNF32-AS3	10q11.21	BC033403	414201	ENSG00000223910		
HGNC:40620	ZNF197-AS1	3p21.31			ENSG00000233509		
HGNC:28586	ZNF205-AS1	16p13.3		81854	ENSG00000263214	12477932	NR_024166
HGNC:50505	ZNF213-AS1	16p13.3		100507458	ENSG00000263072		
HGNC:27821	ZNF252P-AS1	8q24.3	AK092777	286103	ENSG00000255559	12477932	NR_026974
HGNC:23130	ZNF295-AS1	21q22.3		150142	ENSG00000237232		NR_027273
HGNC:40759	ZNF337-AS1	20p11.1			ENSG00000213742		
HGNC:50736	ZNF341-AS1	20q11.22		101929746	ENSG00000230753		
HGNC:41423	ZNF346-IT1	5q35.2			ENSG00000251666		
HGNC:48598	ZNF350-AS1	19q13.41	BC014606, DA066128	101669766	ENSG00000269235		
HGNC:41136	ZNF385D-AS1	3p24.3			ENSG00000225542		

HGNC:42420	ZNF385D-AS2	3p24.3			ENSG00000223351		
HGNC:25122	ZNF436-AS1	1p36.12	BC028411	148898	ENSG00000249087	12477932	NR_033691
HGNC:27370	ZNF503-AS1	10q22.2		253264	ENSG00000226051		
HGNC:23525	ZNF503-AS2	10q22.3	BC029963	100131213	ENSG00000237149		NR_024422
HGNC:51305	ZNF528-AS1	19q13.41		102724105	ENSG00000269834		NR_125345
HGNC:51275	ZNF529-AS1	19q13.12		101927599	ENSG00000233527		NR_110705
HGNC:27613	ZNF561-AS1	19p13.2		284385	ENSG00000267106		NR_122038
HGNC:44324	ZNF571-AS1	19q13.12	BC042488, BX647800	100507433	ENSG00000267470		NR_038247
HGNC:25213	ZNF582-AS1	19q13.43	AW160702, BC036928	386758		12477932	NR_037159
HGNC:41215	ZNF630-AS1	Xp11.23			ENSG00000277541		
HGNC:41434	ZNF638-IT1	2p13.2	BC044944	100507113			
HGNC:51285	ZNF649-AS1	19q13.41		101928571	ENSG00000268095		NR_110733
HGNC:44321	ZNF667-AS1	19q13.43	AW955601, BC110411	100128252	ENSG00000166770		NR_036521
HGNC:44266	ZNF674-AS1	Xp11.23	BC034616, DA707714	401588	ENSG00000230844		NR_015378
HGNC:27617	ZNF790-AS1	19q13.12		284408			NR_040027
HGNC:51303	ZNF793-AS1	19q13.12		101927720	ENSG00000266916		NR_110723
HGNC:41927	ZNRF3-AS1	22q12.1			ENSG00000177993		
HGNC:41440	ZNRF3-IT1	22q12.1			ENSG00000235786		
HGNC:43594	ZRANB2-AS1	1p31.1	BC070115	100132618	ENSG00000235079		NR_038420
HGNC:43595	ZRANB2-AS2	1p31.1	BC054887	100852410	ENSG00000229956		
HGNC:48982	ZSCAN16-AS1	6p22.1		100129195	ENSG00000269293		
HGNC:45103	ZSWIM8-AS1	10q22.2		100507331	ENSG00000272589		NR_038357

Variables	N	LINC00271		*P* value
		High	Low	
**Age (years)**				0.962
**<45**	99	48 (48.5%)	51 (51.5%)	
**≥45**	86	42 (48.8%)	44 (51.2%)	
**Gender**				***0.006***
**Male**	43	13 (30.2%)	30 (69.8%)	
**Female**	142	77 (54.2%)	65 (45.8%)	
**Maximum size of tumor (cm)**	185	1.10±0.72	1.34±0.86	***0.038***
**Multifocality**				0.375
**Unifocal**	135	63 (46.7%)	72 (53.3%)	
**Multifocal**	50	27 (54.0%)	23 (46.0%)	
**Coexistent HT**				0.370
**Yes**	44	24 (54.5%)	20 (45.5%)	
**No**	141	66 (46.8%)	75 (53.2%)	
**PTMC**				***0.030***
**Yes**	100	56 (56.0%)	44 (44.0%)	
**No**	85	34 (40.0%)	51 (60.0%)	
**ETE**				0.378
**Yes**	17	10 (58.3%)	7 (41.2%)	
**No**	168	80 (47.6%)	88 (52.4%)	
**T Stage**				0.714
**T1-T2**	166	80 (48.2%)	86 (51.8%)	
**T3-T4**	19	10 (52.6%)	9 (47.4%)	
**LNM**				***0.024***
**N0**	85	49 (57.6%)	36 (42.4%)	
**N1**	100	41 (41.0%)	59 (59.0%)	
**TNM Stage**				0.685
**I-II**	152	75 (49.3%)	77 (50.7%)	
**III-IV**	33	15 (45.5%)	18 (54.5%)	
$BRAF^{V600E}$				0.625
**Mutation**	103	52 (50.5%)	51 (49.5%)	
**Wild-type**	79	37 (46.8%)	42 (53.2%)	

Table S2. Clinicopathological correlations of LINC00271 expression in the FUSCC cohort.

Notes: Italic and bold type indicates statistical significance. Abbreviations: PTC, papillary thyroid cancer; PTMC, papillary thyroid microcarcinoma; HT, Hashimoto's thyroiditis; ETE, extrathyroidal extension; LNM, lymph node metastasis; TNM, tumor–node–metastasis

Variables	FUSCC cohort			TCGA cohort		
	***P* value** [a]	**OR**	**95.0% CI for OR**	***P* value** [a]	**OR**	**95.0% CI for OR**
**Age** [b]	0.067	1.034	0.998-1.072	*0.535*	1.008	0.983-1.034
**Gender** [b]						
**Male**		1			1	
**Female**	***0.034***	0.422	0.190-0.935	0.884	1.056	0.507-2.199
**Multifocality** [b]						
**Unifocal**		1			1	
**Multifocal**	0.463	0.767	0.378-1.558	0.087	0.557	0.285-1.088
**Histological subtypes** [b]						
**Classical PTC**		1			1	
**Follicular PTC**		NA		0.704	0.824	0.304-2.234
**Tall-cell PTC**		NA		***0.011***	7.903	1.602-38.994
**Coexistent HT** [b]						
**No**		1			1	
**Yes**	0.700	0.863	0.408-1.825	0.405	0.674	0.266-1.708
**ETE** [b]						
**No**		1			1	
**Yes**	0.205	0.422	0.111-1.601	0.685	1.352	0.314-5.819
**T stage** [b]						
**T1-T2**		1			1	
**T3-T4**	0.385	2.415	0.331-17.622	0.654	0.710	0.159-3.178
**LNM** [b]						

**N0**		1			1	
**N1**	***0.013***	2.646	1.225-5.714	***0.034***	2.158	1.060-4.394
**M** [b]						
**M0**		1			1	
**M1**		NA		0.644	1.705	0.178-16.337
**TNM stage** [b]						
**I-II**		1			1	
**III-IV**	*0.349*	0.592	0.197-1.777	*0.687*	1.225	0.458-3.278
BRAF ^{V600E} **b**						
**Wild-type**		1			1	
**Mutation**	0.400	0.761	0.402-1.439	*0.077*	1.842	0.936-3.626

Table S3. Multivariate analysis of factors that might affect LINC00271 expression in PTC in the TCGA and FUSCC cohorts. Notes: a, Italic and bold type indicates statistical significance; b, adjusted by all factors in multivariate analysisAbbreviations: PTC, papillary thyroid cancer; FUSCC, Fudan University Shanghai Cancer Center; TCGA, The Cancer Genomics Atlas; OR, odds ratio; CI, confidence interval; HT, Hashimoto's thyroiditis; ETE, extrathyroidal extension; LNM, lymph node metastasis; M, metastasis; NA, not available; TNM, tumor-node-metastasis

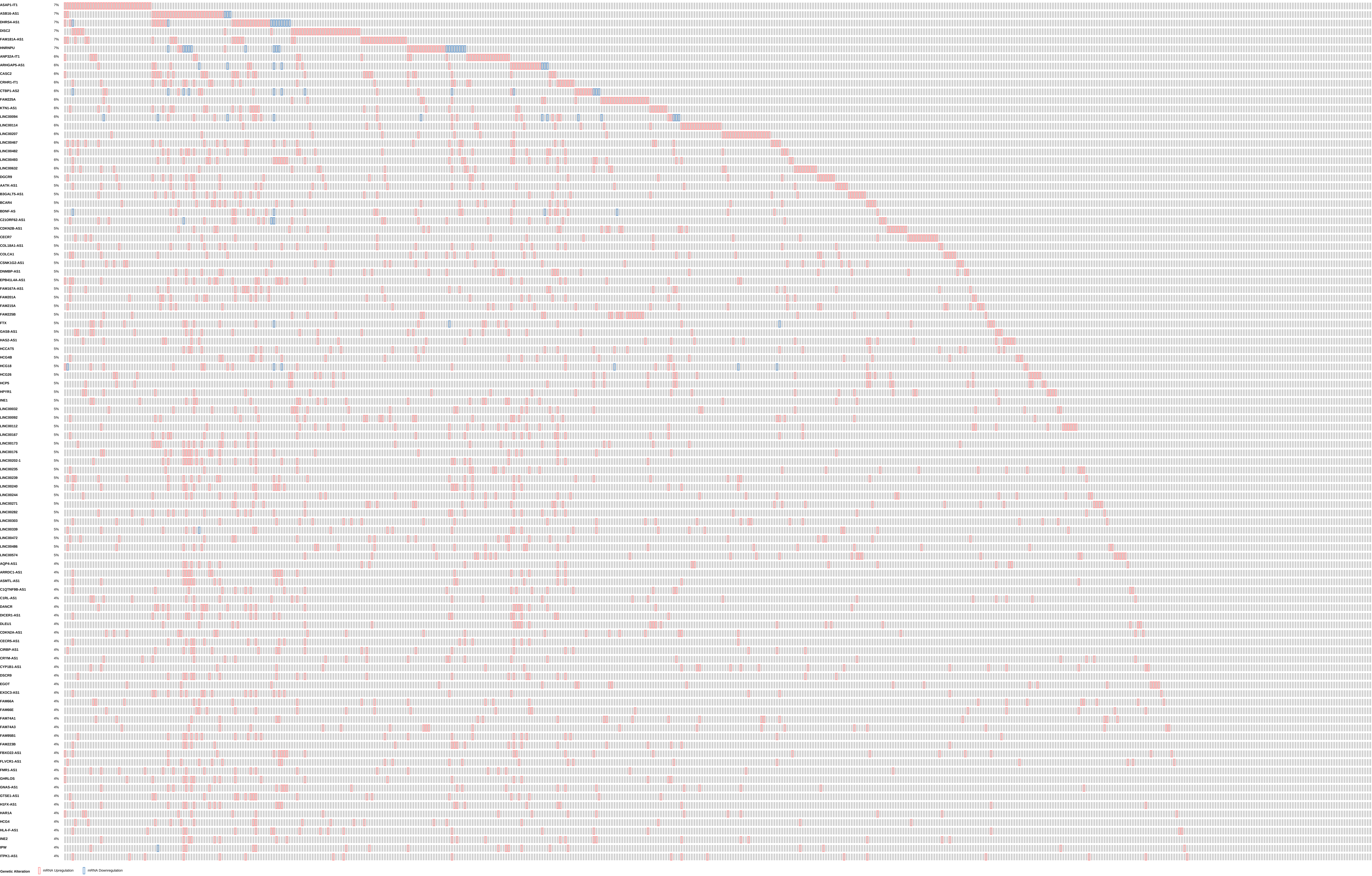
ASAP1-IT1 7%
ASB16-AS1 7%
DHRS4-AS1 7%
DISC2 7%
FAM181A-AS1 7%
HNRNPU 7%
ANP32A-IT1 6%
ARHGAP5-AS1 6%
CASC2 6%
CRHR1-IT1 6%
CTBP1-AS2 6%
FAM225A 6%
KTN1-AS1 6%
LINC00094 6%
LINC00114 6%
LINC00207 6%
LINC00467 6%
LINC00482 6%
LINC00493 6%
LINC00632 6%
DGCR9 5%
AATK-AS1 5%
B3GALT5-AS1 5%
BCAR4 5%
BDNF-AS 5%
C21ORF62-AS1 5%
CDKN2B-AS1 5%
CECR7 5%
COL18A1-AS1 5%
COLCA1 5%
CSNK1G2-AS1 5%
DNMBP-AS1 5%
EPB41L4A-AS1 5%
FAM167A-AS1 5%
FAM201A 5%
FAM215A 5%
FAM225B 5%
FTX 5%
GAS8-AS1 5%
HAS2-AS1 5%
HCCAT5 5%
HCG4B 5%
HCG18 5%
HCG26 5%
HCP5 5%
HPYR1 5%
INE1 5%
LINC00032 5%
LINC00092 5%
LINC00112 5%
LINC00167 5%
LINC00173 5%
LINC00176 5%
LINC00202-1 5%
LINC00235 5%
LINC00239 5%
LINC00240 5%
LINC00244 5%
LINC00271 5%
LINC00282 5%
LINC00303 5%
LINC00339 5%
LINC00472 5%
LINC00486 5%
LINC00574 5%
AQP4-AS1 4%
ARRDC1-AS1 4%
ASMTL-AS1 4%
C1QTNF9B-AS1 4%
C1RL-AS1 4%
DANCR 4%
DICER1-AS1 4%
DLEU1 4%
CDKN2A-AS1 4%
CECR5-AS1 4%
CIRBP-AS1 4%
CRYM-AS1 4%
CYP1B1-AS1 4%
DSCR9 4%
EGOT 4%
EXOC3-AS1 4%
FAM66A 4%
FAM66E 4%
FAM74A1 4%
FAM74A3 4%
FAM95B1 4%
FAM223B 4%
FBXO22-AS1 4%
FLVCR1-AS1 4%
FMR1-AS1 4%
GHRLOS 4%
GNAS-AS1 4%
GTSE1-AS1 4%
H1FX-AS1 4%
HAR1A 4%
HCG4 4%
HLA-F-AS1 4%
INE2 4%
IPW 4%
ITPK1-AS1 4%
Genetic Alteration
mRNA Upregulation
mRNA Downregulation

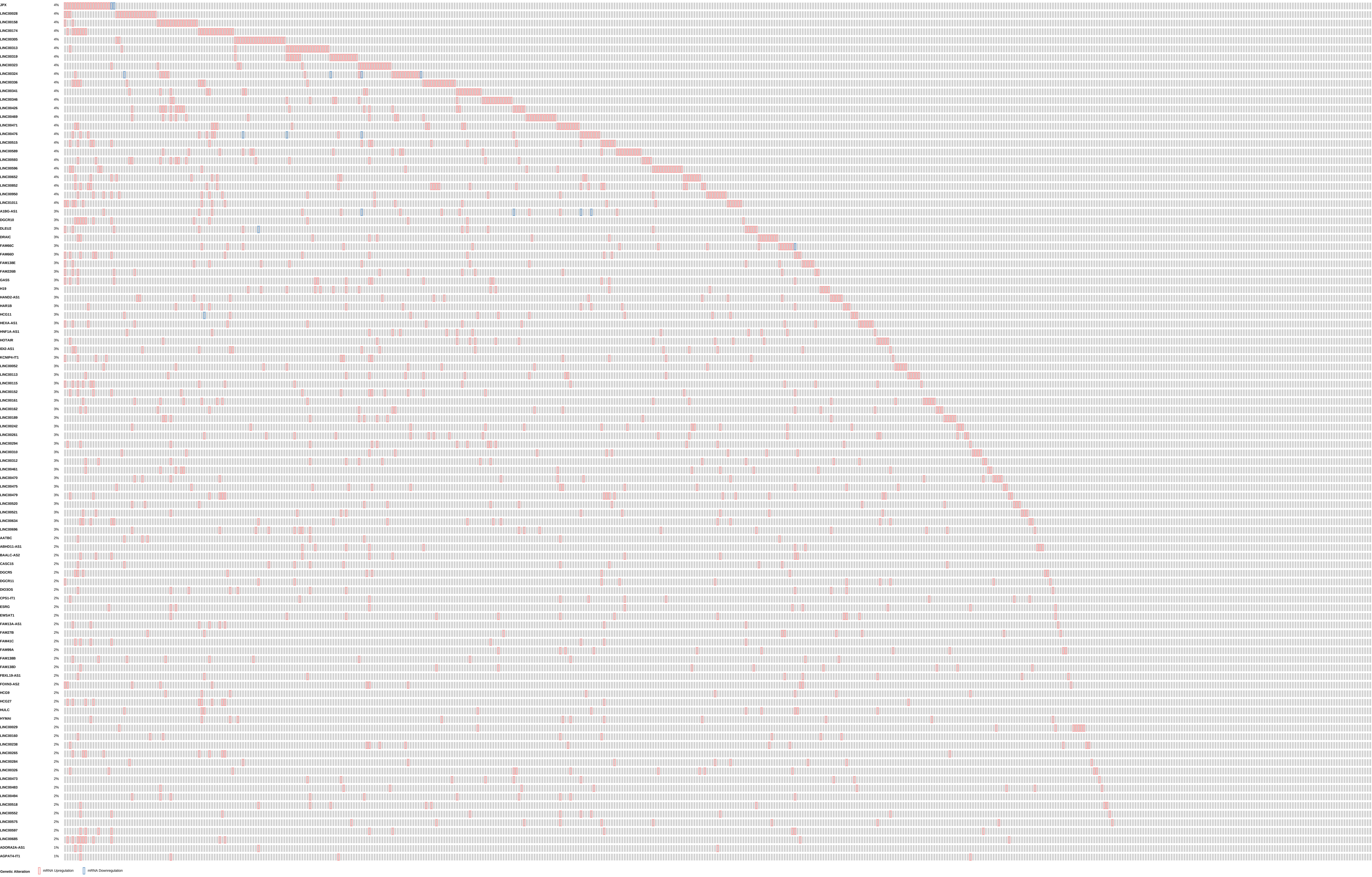
JPX 4%
LINC00028 4%
LINC00158 4%
LINC00174 4%
LINC00305 4%
LINC00313 4%
LINC00319 4%
LINC00323 4%
LINC00324 4%
LINC00336 4%
LINC00341 4%
LINC00346 4%
LINC00426 4%
LINC00469 4%
LINC00471 4%
LINC00476 4%
LINC00515 4%
LINC00589 4%
LINC00593 4%
LINC00596 4%
LINC00652 4%
LINC00852 4%
LINC00950 4%
LINC01011 4%
A1BG-AS1 3%
DGCR10 3%
DLEU2 3%
DRAIC 3%
FAM66C 3%
FAM66D 3%
FAM138E 3%
FAM226B 3%
GAS5 3%
H19 3%
HAND2-AS1 3%
HAR1B 3%
HCG11 3%
HEXA-AS1 3%
HNF1A-AS1 3%
HOTAIR 3%
IDI2-AS1 3%
KCNIP4-IT1 3%
LINC00052 3%
LINC00113 3%
LINC00115 3%
LINC00152 3%
LINC00161 3%
LINC00162 3%
LINC00189 3%
LINC00242 3%
LINC00261 3%
LINC00294 3%
LINC00310 3%
LINC00312 3%
LINC00461 3%
LINC00470 3%
LINC00475 3%
LINC00479 3%
LINC00520 3%
LINC00521 3%
LINC00634 3%
LINC00696 3%
AATBC 2%
ABHD11-AS1 2%
BAALC-AS2 2%
CASC15 2%
DGCR5 2%
DGCR11 2%
DIO3OS 2%
CPS1-IT1 2%
ESRG 2%
EWSAT1 2%
FAM13A-AS1 2%
FAM27B 2%
FAM41C 2%
FAM99A 2%
FAM138B 2%
FAM138D 2%
FBXL19-AS1 2%
FOXN3-AS2 2%
HCG9 2%
HCG27 2%
HULC 2%
HYMAI 2%
LINC00029 2%
LINC00160 2%
LINC00238 2%
LINC00265 2%
LINC00284 2%
LINC00326 2%
LINC00473 2%
LINC00483 2%
LINC00494 2%
LINC00518 2%
LINC00552 2%
LINC00575 2%
LINC00597 2%
LINC00685 2%
ADORA2A-AS1 1%
AGPAT4-IT1 1%
Genetic Alteration
mRNA Upregulation
mRNA Downregulation

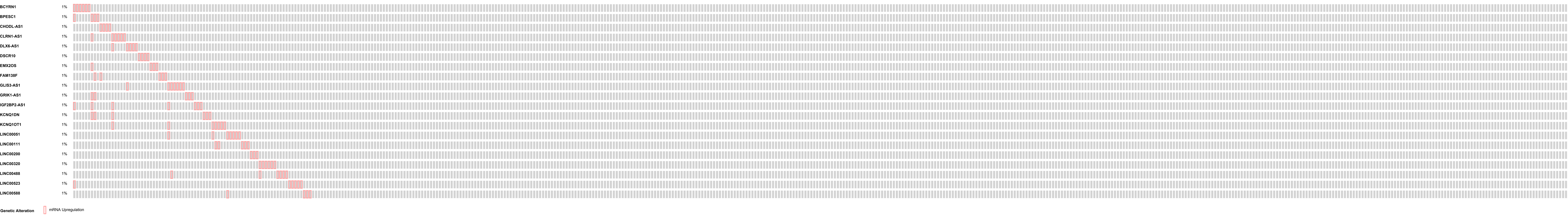

Figure S1. The expression alteration frequency of the 220 lncRNAs ranged from 1% to 7% in 486 PTC patients with RNA sequencing data

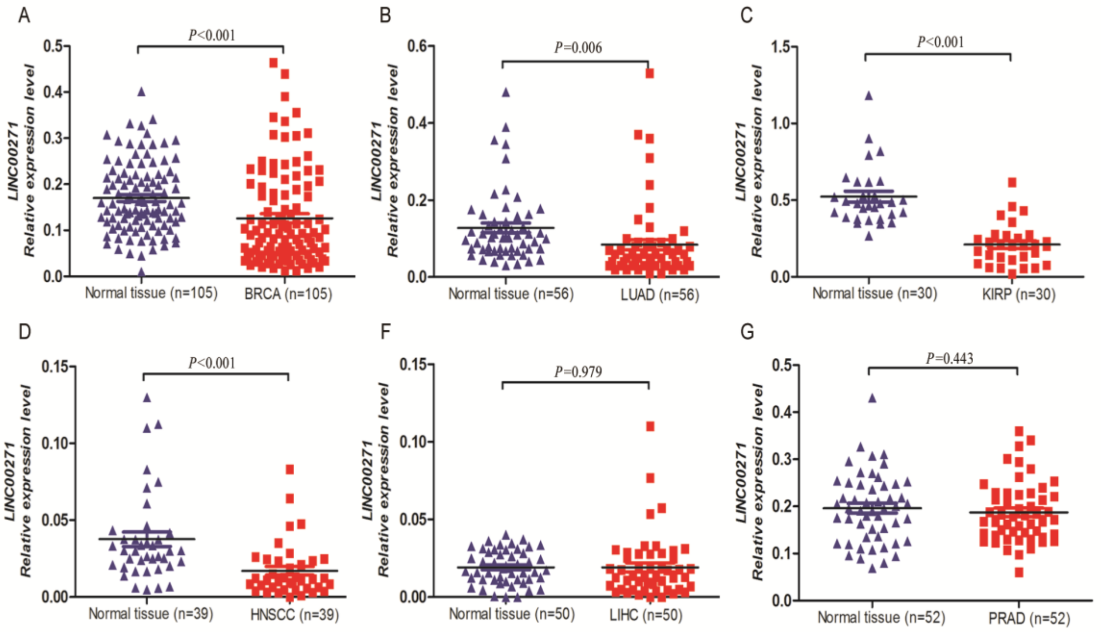

Figure S2. **A comparison of LINC00271 expression between carcinomas and normal tissues in BRCA, LUAD, KIRP, HNSCC, LIHC and PRAD.** A-D showed LIN00271 expression was significantly suppressed in carcinomas compared with normal tissues in BRCA, LUAD, KIRP and HNSCC. F and G indicated no significant difference in LINC00271 expression between carcinomas and normal tissues in LIHC and PRAD.

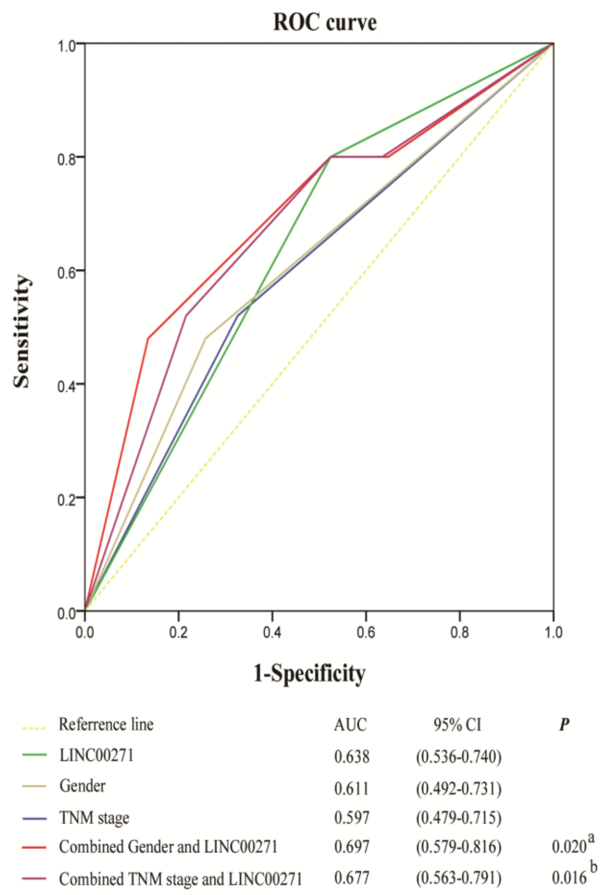

Figure S3. **ROC analysis for the predictive values of LINC00271, gender and TNM stage for PTC recurrence**. a, comparison between Gender and combined gender and LINC00271; b, comparison between TNM stage and combined TNM stage and LINC00271. After combining LINC00271, the AUC values of gender and TNM stage for predicting recurrence were significantly elevated from 0.611 to 0.697 (*P*=0.020) and 0.597 to 0.677 (*P*=0.016), respectively.
